# CMS4 epithelial-intrinsic glycosyltransferase GALNT5 promotes tumor aggressiveness and correlates with poor survival in colorectal cancer

**DOI:** 10.1038/s41598-026-57104-1

**Published:** 2026-06-08

**Authors:** Sohei Hayashishita, Hirokazu Okayama, Shotaro Nakajima, Katsuharu Saito, Chiaki Takiguchi, Dai Mitsui, Hajime Matsuida, Masanori Katagata, Takahiro Sato, Takuro Matsumoto, Daisuke Ujiie, Shun Chida, Zenichiro Saze, Motonobu Saito, Kosaku Mimura, Tomoyuki Momma, Koji Kono

**Affiliations:** 1 Department of Gastrointestinal Tract Surgery, Fukushima Medical University School of Medicine, 1 Hikarigaoka, Fukushima city, 960-1295 Fukushima Japan; 2 Department of Multidisciplinary Treatment of Cancer and Regional Medical Support, Fukushima Medical University School of Medicine, Fukushima, Japan; 3 Department of Blood Transfusion and Transplantation Immunology, Fukushima Medical University School of Medicine, Fukushima, Japan

**Keywords:** Colorectal cancer (CRC), Epithelial-mesenchymal transition (EMT), CMS4, Glycosyltransferase, GALNT5, Biomarkers, Cancer, Computational biology and bioinformatics, Oncology

## Abstract

The “mesenchymal” consensus molecular subtype 4 (CMS4) of colorectal cancer (CRC) has the poorest prognosis and shows stromal activation and bulk epithelial-mesenchymal transition (EMT) signatures that largely reflect cancer-associated fibroblast-derived signals, obscuring tumor cell-intrinsic programs. Aberrant glycosylation drives EMT and metastasis, yet the glycosyltransferase landscape intrinsic to CMS4 tumor epithelium remains poorly defined because stromal signals dominate bulk transcriptomes. To overcome this confounding, we analyzed single-cell RNA-sequencing datasets to distinguish CMS4 tumor epithelial cells from CMS1–3 epithelial cells, normal epithelium, and stromal/immune populations. This identified a nine-gene glycosyltransferase signature uniquely upregulated in CMS4 tumor epithelium. Among these, GALNT5 showed consistent transcriptional induction during EMT in CRC cell lines. GALNT5 depletion suppressed proliferation, invasion, and migration, independent of overt changes in canonical EMT markers or AKT/ERK signaling. Immunohistochemical analysis of 431 resected CRC specimens confirmed that GALNT5 protein expression was restricted to cancer cells, particularly at the invasive front. High GALNT5 expression was associated with deeper invasion and advanced stage, and served as an independent prognostic factor for worse overall survival, notably within the microsatellite-stable subgroup. In conclusion, by leveraging single-cell transcriptomics, this study defines a CMS4 epithelial-intrinsic glycosylation program and highlights GALNT5 as a prognostic biomarker and therapeutic candidate.

**Supplementary Information:**

The online version contains supplementary material available at 10.1038/s41598-026-57104-1.

## Introduction

Colorectal cancer (CRC) remains a leading cause of cancer-related mortality worldwide, driven primarily by metastatic dissemination^1^. Large-scale bulk transcriptomic studies have demonstrated that the tumor microenvironment (TME), particularly TGFβ-driven cancer-associated fibroblast (CAF) activation, contributes to promoting metastasis and correlates with adverse prognosis^2–5^. These efforts led to the establishment of the Consensus Molecular Subtypes (CMS), a bulk transcriptome-based classification that captures gene expression signals originating from malignant epithelial cells as well as the surrounding stromal/immune compartments^6^. Among the four subtypes, “mesenchymal” CMS4 comprises 20–25% of cases and has the poorest prognosis, characterized by prominent TGFβ signaling activation, stromal infiltration, angiogenesis, and epithelial-mesenchymal transition (EMT)-associated gene signatures^6,7^.

EMT is a dynamic, transcriptionally driven program in which epithelial cancer cells lose polarity and cell-cell adhesion and acquire mesenchymal traits, enabling motility, invasiveness, and stem-like properties. This cellular plasticity is commonly induced by TGFβ and is associated with metastasis and therapy resistance. Within the “mesenchymal” CMS4 TME, activated CAFs contribute to the aggressive phenotype, while malignant epithelial cells are hypothesized to undergo EMT^7–9^. However, because CAF and EMT-state cancer cells share highly similar transcriptional programs, bulk EMT signatures largely reflect stromal/CAF abundance rather than epithelial-intrinsic EMT^10–12^. This stromal dominance masks epithelial-intrinsic features of the relatively small fraction of CMS4 cancer cells, complicating molecular profiling by conventional bulk transcriptomics.

Aberrant glycosylation, an enzymatic, post-translational modification of proteins and lipids to form glycan structures, is a hallmark of cancer^13^. During tumor progression, the gene expression of glycosyltransferases, the enzymes responsible for glycan biosynthesis, as well as the resulting cell-surface glycan structures are extensively altered, influencing proliferation, adhesion, invasion, and metastasis. Glycosylation is also linked to EMT, often characterized by specific glycosyltransferases that promote mesenchymal traits. For instance, MGAT5, several fucosyltransferases (FUT3, FUT6, FUT8), and members of the GALNT family are frequently upregulated during EMT and promote invasion and metastasis by modifying key receptors, including TGFβ receptors and other glycoproteins^14–17^. These observations highlight glycosylation as an important regulatory layer of epithelial plasticity within the TME.

Given the high clinical risk of CMS4 and the link between aberrant glycosylation and EMT, the epithelial-intrinsic glycosyltransferase program in CMS4 tumors remains poorly defined, largely due to stromal confounding in bulk transcriptomics. We therefore employed single-cell RNA-sequencing (scRNA-seq) to define a CMS4 epithelial-specific glycosyltransferase signature by comparing CMS4 tumor epithelial cells across normal epithelial, stromal, and immune populations. We identified glycosyltransferase genes uniquely upregulated in CMS4 epithelium, many of which have been linked to EMT, and prioritized GALNT5 for functional characterization and clinical validation by immunohistochemistry (IHC).

## Results

### Identification of nine glycosyltransferase genes specifically upregulated in CMS4 CRC epithelial cells

**Fig. 1:**
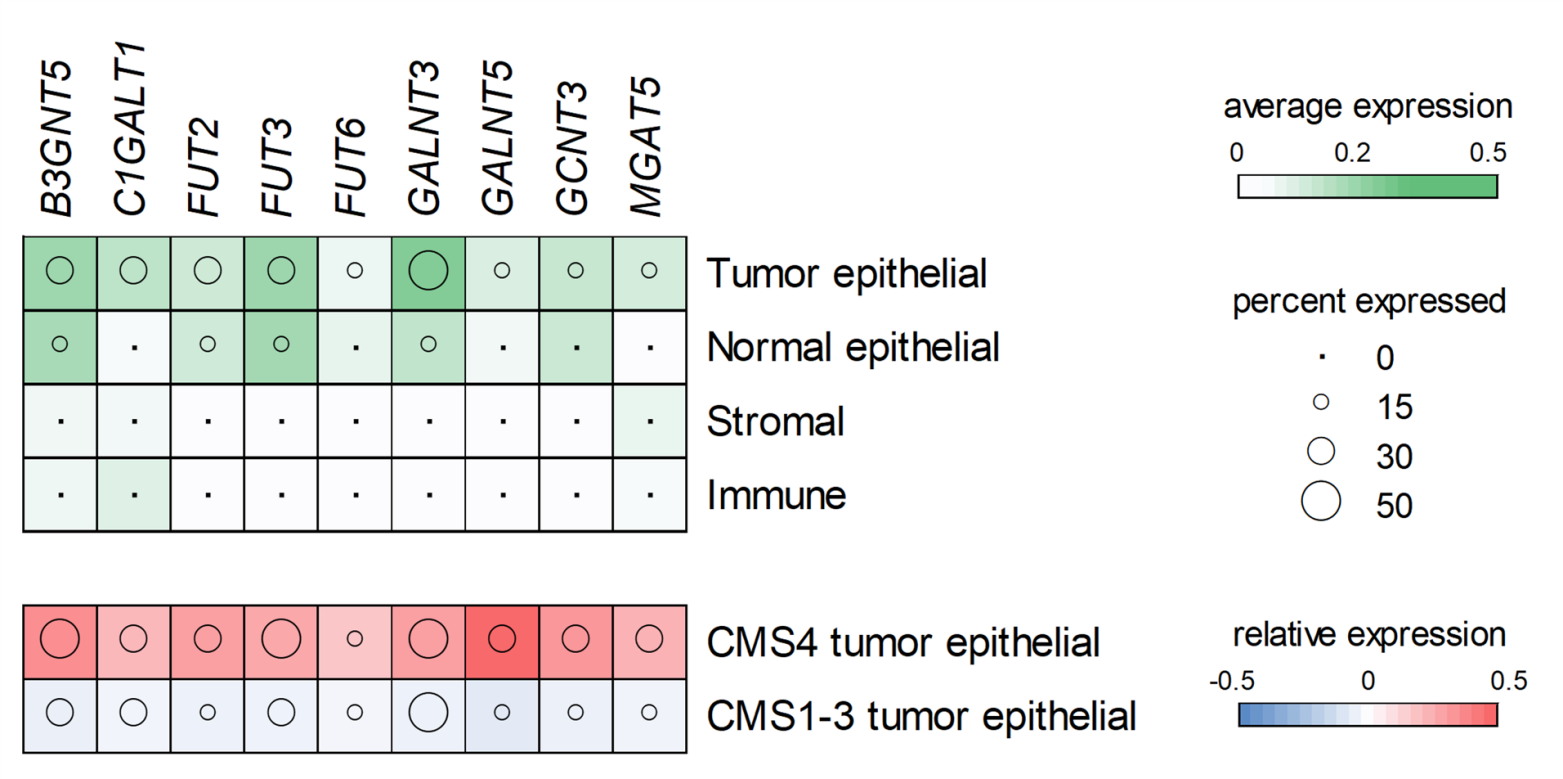
scRNA-seq analysis of 188 glycosyltransferase genes identified nine genes specifically upregulated in CMS4 tumor epithelial cells (GSE132465). Dot plots show average expression (color) and the percentage of expressing cells (dot size) in each cell type. (Upper panel) Expression of the nine genes across four cell populations: tumor epithelial cells (*n* = 17,469), normal epithelial cells (*n* = 1070), stromal cells (*n* = 2736), and immune cells (*n* = 27,080). (Lower panel) Comparison of the nine genes between CMS4 tumor epithelial cells (*n* = 2171) and CMS1–3 tumor epithelial cells (*n* = 15,298).

Utilizing the scRNA-seq dataset GSE132465^18^, we analyzed 48,355 single cells and screened a panel of 188 glycosyltransferase genes (Supplementary Table S1)^19^. We applied a two-step filtering scheme to identify genes enriched in CMS4 tumor epithelium while minimizing non-epithelial signals. Step-1 (epithelial CMS4 enrichment) selected genes (i) expressed in > 15% of all tumor epithelial cells and (ii) upregulated in CMS4 versus CMS1–3 tumor epithelium and CMS4 versus normal epithelium (log_2_FC > 0.4 for each comparison), yielding 19 candidates (Supplementary Table S1 and Supplementary Fig. S1). Step-2 (non-epithelial depletion) retained only genes expressed in < 15% of stromal and immune cells, refining the list to nine glycosyltransferase genes: *B3GNT5*, *C1GALT1*, *FUT2*, *FUT3*, *FUT6*, *GALNT3*, *GALNT5*, *GCNT3*, and *MGAT5* (Supplementary Table S1 and Supplementary Fig. S1). As shown in Fig. 1, these genes were consistently and predominantly expressed in tumor epithelial cells, with only low expression in normal epithelium and minimal expression in stromal or immune cells, and were enriched in CMS4 compared with CMS1–3 epithelial cells. Collectively, they define a CMS4-specific, tumor epithelial-intrinsic glycosylation signature, successfully decoupled from immune or stromal transcriptional signals.

Notably, several genes in this panel, including *MGAT5*, *FUT3*, *FUT6*, and *GCNT3*, as well as additional candidates identified in the initial screen, such as *FUT8* and *CHPF*, have been implicated in promoting EMT and metastatic behavior in CRC and other malignancies, often in the context of TGFβ signaling^14–16,20−27^. These observations support a model in which CMS4 tumor epithelial cells within the CAF-rich microenvironment might actively engage EMT programs, likely contributing to the aggressive phenotype of this subtype.

### Validation of tumor epithelial cell specificity of nine glycosyltransferase genes

**Fig. 2:**
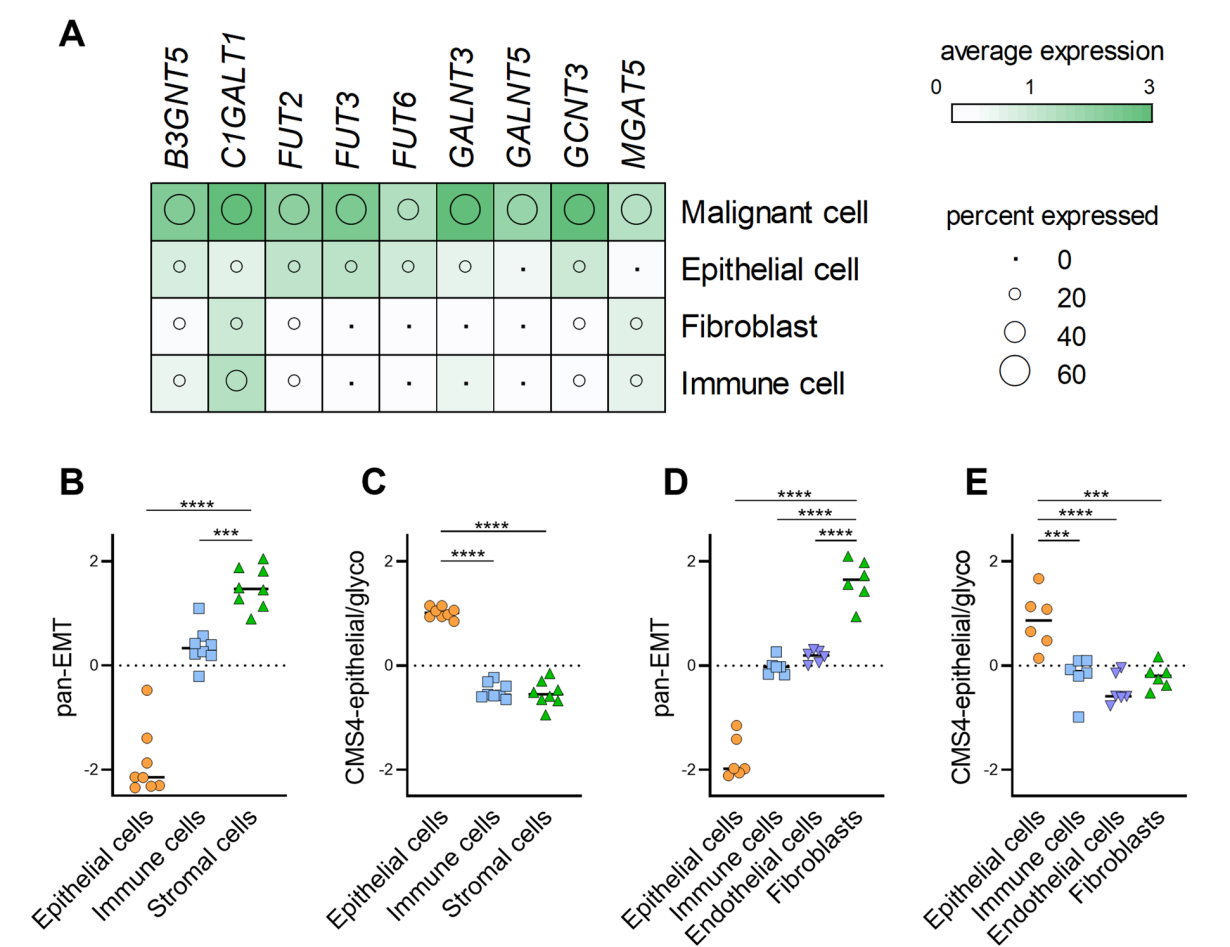
Validation of malignant epithelial cell-specificity of the nine glycosyltransferase genes. (**A**) In an independent scRNA-seq dataset (GSE146771), all nine genes showed the highest expression and the largest expressing fractions in malignant cells (*n* = 269), compared with non-malignant epithelial cells (*n* = 855), fibroblasts (*n* = 138), and immune cells (*n* = 6387). Dot plots represent average expression (color) and the percentage of expressing cells (dot size) in each cell type. (**B**–**E**) Levels of the pan-cancer EMT signature (pan-EMT) (**B**, **D**) and the nine-gene CMS4-epithelial glycosyltransferase signature (CMS4-epithelial/glyco) (**C**, **E**) in FACS-purified cell populations from CRC: three cell types from eight tumors (GSE39395) (**B**, **C**) and four cell types from six tumors (GSE39396) (**D**, **E**). ****P < 0.0001, ***P < 0.001.

Given that bulk EMT signatures often reflect CAF-derived rather than epithelial intrinsic signals^10,12^, we next validated the malignant epithelial cell specificity of our nine genes. We first examined an independent scRNA-seq dataset (GSE146771), in which malignant cells were distinguished by inferred chromosomal copy-number variations (CNVs)^28^. As shown in Fig. 2A, all nine genes exhibited the highest average expression and largest expressing fractions in malignant cells, whereas expression was sparse in fibroblasts and immune cells and only modest in non-malignant epithelial cells. The cell-level expression distributions of the nine genes across the relevant cell populations are additionally shown as violin plots in Supplementary Fig. S2, further supporting the patterns observed in Figs. 1 and 2A. We then constructed a nine-gene CMS4 epithelial glycosyltransferase signature (CMS4-epithelial/glyco signature) and compared it with a previously established pan-cancer EMT signature^29^ using two microarray datasets of FACS-purified cell populations (GSE39395 and GSE39396)^4^. In GSE39395, the pan-EMT signature was enriched in stromal cells while diminished in epithelial cells, whereas the CMS4-epithelial/glyco signature was selectively elevated in epithelial cells with negligible expression in immune and stromal fractions (Fig. 2B, C). In GSE39396, the pan-EMT signature was highest in fibroblasts and lowest in epithelial cells, while the CMS4-epithelial/glyco signature was again highest in epithelial cells and remained near baseline in non-epithelial populations (Fig. 2D, E). Together with the scRNA-seq analysis, these multi-platform data support that the nine-gene panel is genuinely tumor epithelial–intrinsic and largely decoupled from confounding CAF- or immune-derived transcriptional signals. Consistent with the stromal dominance of bulk CMS4 mesenchymal signatures, the CMS4-epithelial/glyco signature was not selectively increased in bulk CMS4 tumors in TCGA or AC-ICAM datasets (Supplementary Fig. S3).

### GALNT5 was transcriptionally upregulated during EMT-like changes in CRC cell lines

**Fig. 3:**
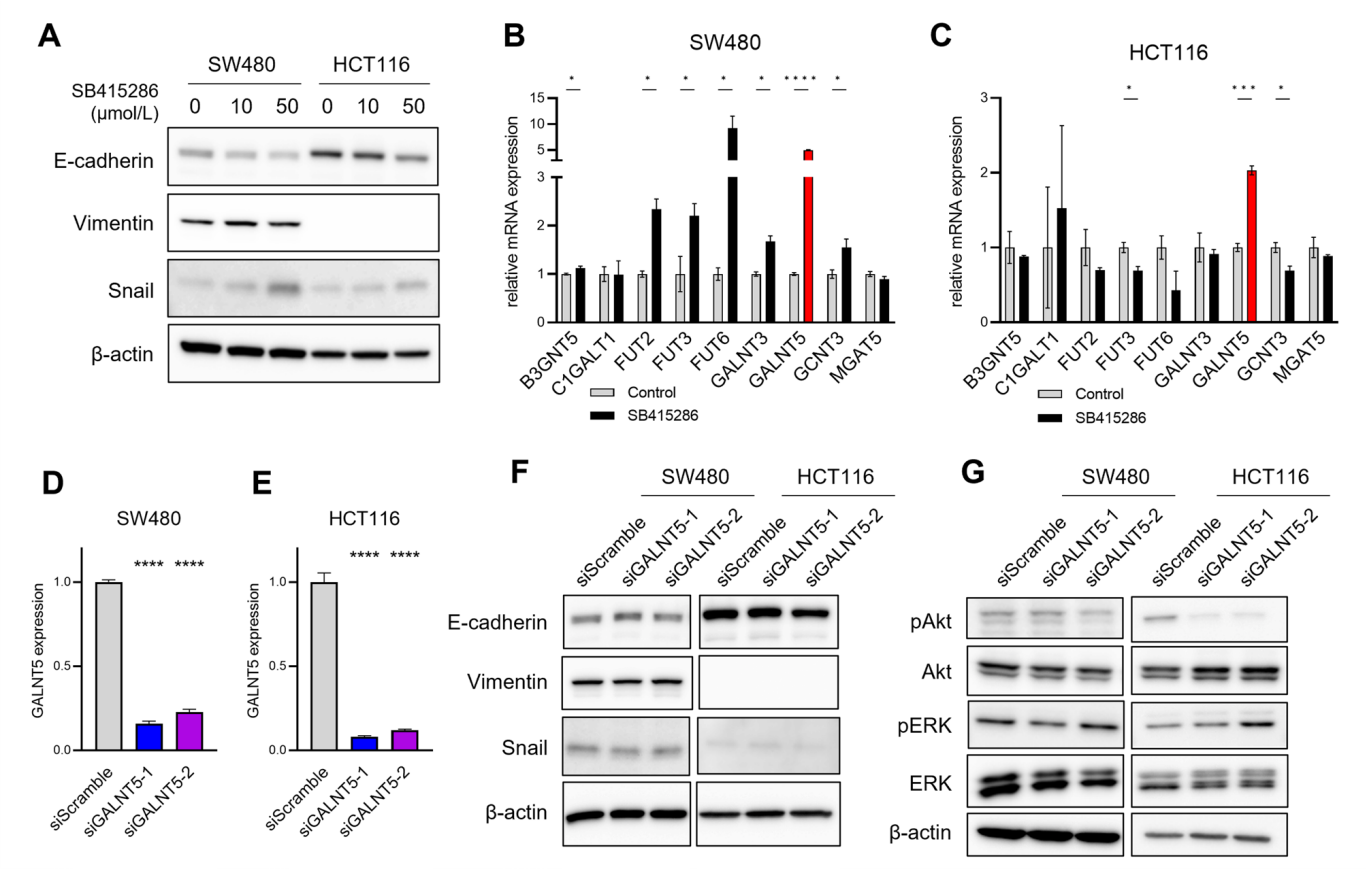
GALNT5 is induced by GSK3 inhibition-mediated EMT-like conditions, and its knockdown shows limited effects on EMT markers and AKT/ERK signaling. (**A**) Western blot analysis of EMT markers in HCT116 and SW480 cells treated with the GSK3 inhibitor SB415286 or vehicle control. SB415286 decreased E-cadherin and increased Snail in both cell lines, consistent with EMT induction. (**B**, **C**) RT-qPCR analysis of the nine glycosyltransferase genes in SW480 (**A**) and HCT116 (**C**) cells after SB415286 treatment (20 µmol/L). Data are expressed as fold change relative to controls. Multiple unpaired *t*-test. (**D**, **E**) RT-qPCR showing reduced GALNT5 mRNA levels after transfection with two independent siRNAs versus non-targeting control in SW480 (**D**) and HCT116 (**E**) cells. One-way ANOVA. (**F**) EMT marker expression after GALNT5 knockdown. (**F**) AKT/ERK signaling after GALNT5 knockdown. ****P < 0.0001, ***P < 0.001, **P < 0.01, *P < 0.05.

We next examined the expression of the nine genes in CRC cell lines under experimental EMT induction. In both HCT116 (*TGFBR2*-mutant) and SW480 (*SMAD4*-deficient) cells, treatment with the GSK3 inhibitor SB415286 effectively induced EMT-like changes, including decreased E-cadherin and increased Snail by western blotting (Fig. 3A and Supplementary Fig. S4), together with morphological changes (Supplementary Fig. S5). RT-qPCR showed a reproducible 2–5-fold upregulation of *GALNT5* in both cell lines after SB415286 treatment (Fig. 3B, C). In SW480 cells, modest increases in *B3GNT5*, *FUT2*, *FUT3*, *FUT6*, *GALNT3*, and *GCNT3* were also observed, but these changes were not reproduced in HCT116 cells. We also treated cells with TGFβ. As reported previously^30^, TGFβ induced only minor changes in EMT markers in SW480 cells and none in HCT116 cells (Supplementary Fig. S6A). Consistent with this, expression of the nine genes did not significantly change in either cell line after TGFβ treatment (Supplementary Fig. S6B, C).

## GALNT5 loss impaired proliferation and migration independent of consistent EMT marker modulation or AKT/ERK activation

**Fig. 4:**
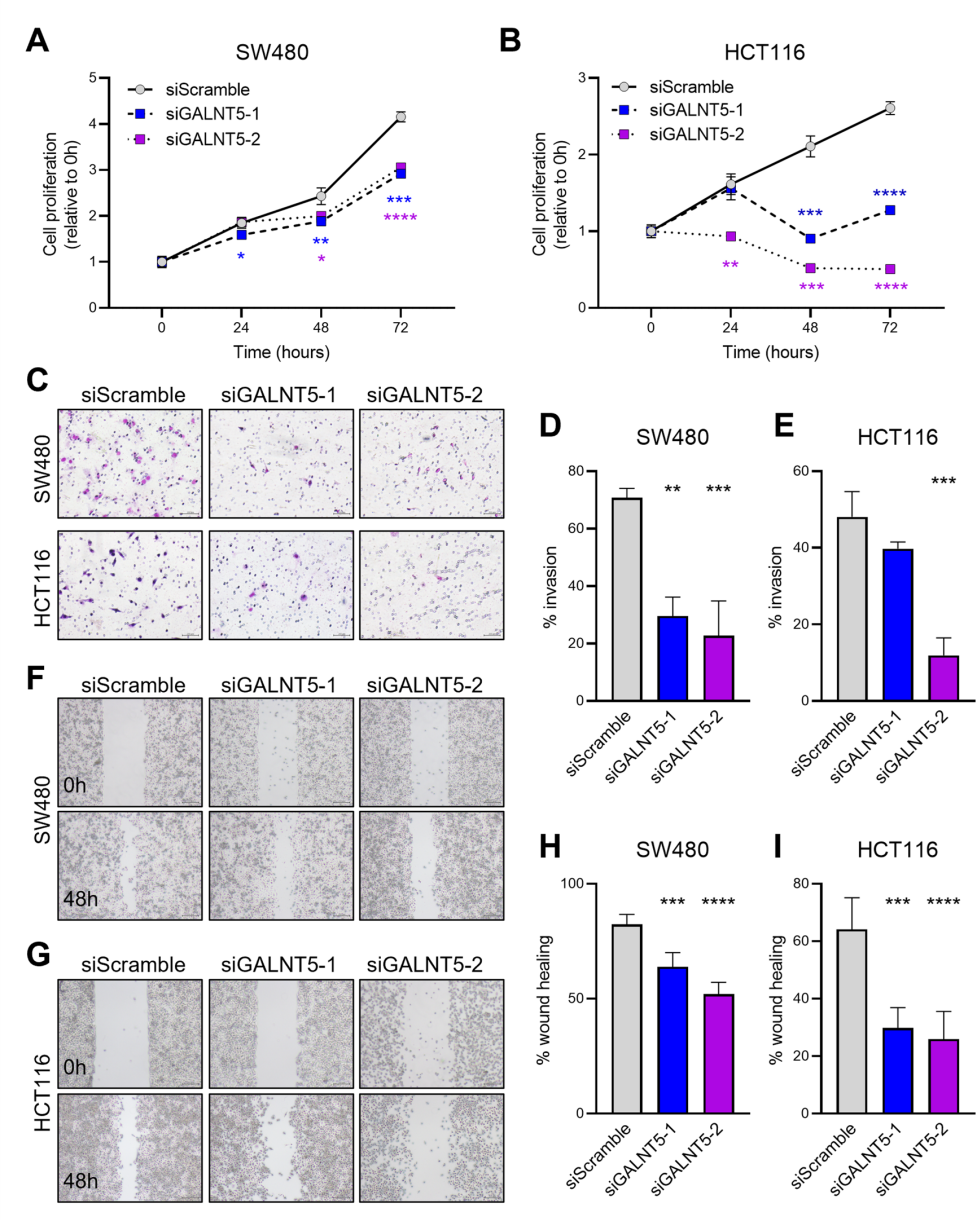
GALNT5 knockdown suppresses proliferation, invasion, and migration of CRC cells. (**A**, **B**) Effects of GALNT5 knockdown on cell proliferation in SW480 (**A**) and HCT116 (**B**) cells by CCK-8 assay. Two-way ANOVA. (**C**–E) Transwell invasion assays with Giemsa staining after GALNT5 knockdown. Representative images of invaded cells (**C**), and quantification in SW480 (**D**) and HCT116 (**E**) cells. One-way ANOVA. (**F**–**I**) Wound-healing assays evaluating cell migration after GALNT5 knockdown. Representative images at the indicated time points are shown for SW480 (**F**) and HCT116 (**G**) cells, with quantification of wound closure in SW480 (**H**) and HCT116 (**I**) cells. One-way ANOVA. Bar graphs show mean ± SD from at least three independent experiments. ****P < 0.0001, ***P < 0.001, **P < 0.01, *P < 0.05.

Because *GALNT5* was consistently upregulated under SB415286-induced EMT-like conditions, we next examined its functional role using siRNA-mediated knockdown in SW480 and HCT116 cells. Efficient depletion of GALNT5 at both mRNA and protein levels was achieved with two independent siRNAs (Fig. 3D, E and Supplementary Fig. S7). At baseline, GALNT5 knockdown did not significantly alter EMT marker expression in neither cell lines (Fig. 3F and Supplementary Fig. S8A). When EMT-like changes were induced by SB415286, concomitant GALNT5 depletion did not attenuate the resulting mesenchymal phenotype, suggesting that GALNT5 does not appear to be required for robust EMT activation in this setting (Supplementary Fig. S9). We then assessed signaling pathways previously linked to GALNT5 in other cancers^31^. In SW480 cells, GALNT5 knockdown did not detectably change phosphorylation of AKT (pAKT) or ERK (pERK). In HCT116 cells, we observed a modest reduction in pAKT, while pERK remained unchanged (Fig. 3G and Supplementary Fig. S8B). Despite these limited and cell line-dependent effects on EMT markers and AKT/ERK signaling, GALNT5 depletion consistently reduced cell proliferation and markedly impaired the invasive and migratory capacities in both cell lines, as shown by CCK-8 (Cell Counting Kit-8), transwell invasion, and wound-healing assays (Fig. 4A–I). Together, these results suggest that GALNT5 promotes proliferation, invasion, and migration of CRC cells, largely independently of major changes in EMT marker expression or AKT/ERK pathway activation.

## GALNT5 protein expression by IHC correlated with poor prognosis

**Fig. 5:**
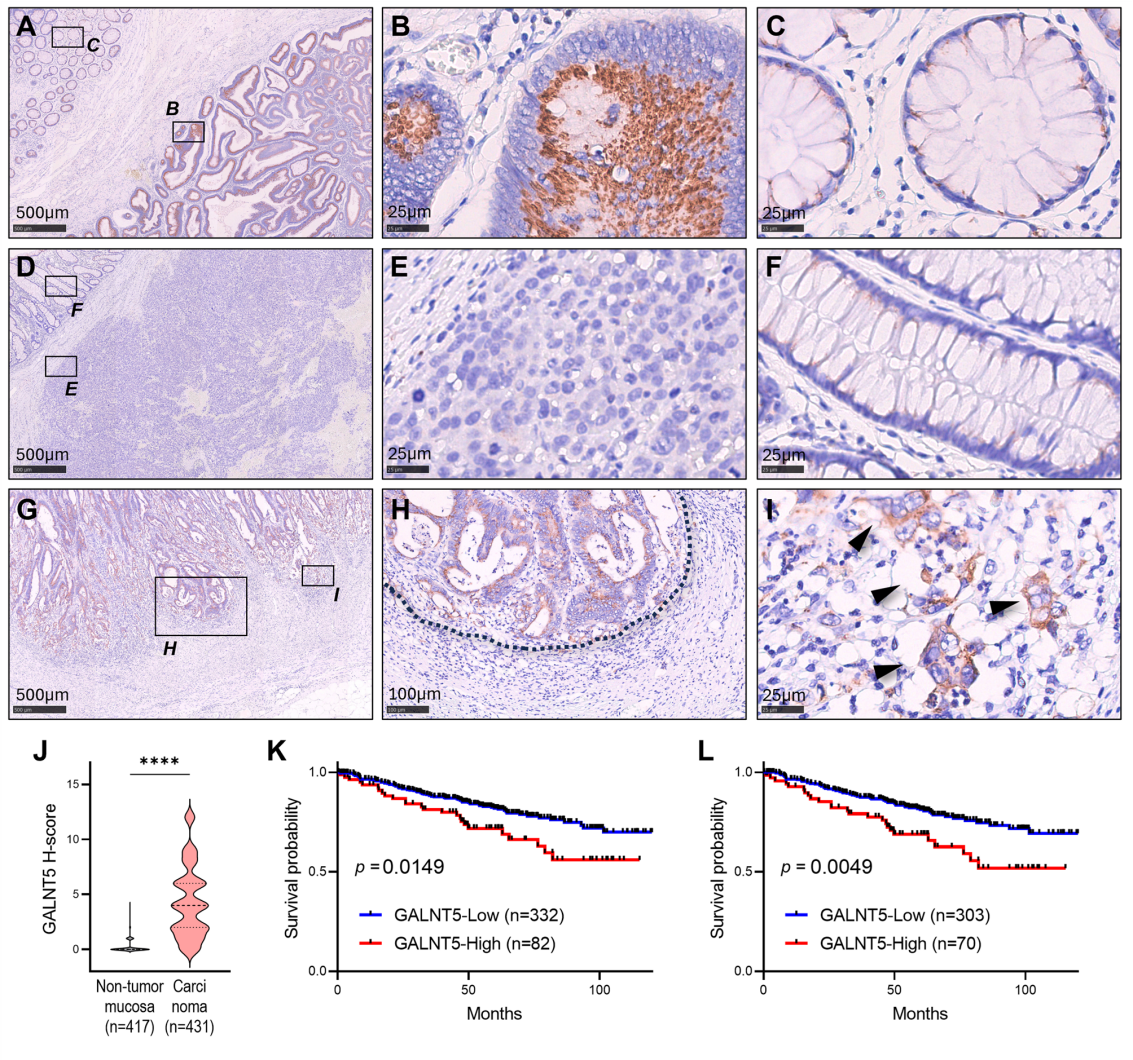
IHC of GALNT5 in resected CRC specimens and association with prognosis. (**A**–**C**) Representative images of a GALNT5-High CRC (**A**), showing intense cytoplasmic staining in malignant epithelial cells (**B**) and faint staining in adjacent non-neoplastic mucosa (**C**). (**D**–**F**) Representative images of a GALNT5-Low CRC (**D**), in which tumor cells lacked detectable GALNT5 staining (**E**), with faint staining in adjacent non-neoplastic mucosa (**F**). (**G**–**I**) Representative images of a GALNT5-High CRC highlighting localization of GALNT5-positive tumor cells at the invasive front (**F**, **H**) and within tumor budding foci (arrowheads) (I). GALNT5 staining was restricted to malignant epithelial cells, with no detectable staining in stromal or immune compartment (**A**–**I**). (**J**) GALNT5 H-score in non-tumor mucosa (*n* = 417) and CRC (*n* = 431). Mann–Whitney U test. ****P < 0.0001. (**K**) Kaplan–Meier analysis of OS in stage I–IV CRC patients stratified by GALNT5 expression (*n* = 414). (**L**) Kaplan–Meier analysis of OS in pMMR/MSS CRC patients stratified by GALNT5 expression (*n* = 373). Log-rank *p*-values are shown.

We then performed IHC for GALNT5 protein in primary CRC specimens to examine its clinicopathological and prognostic relevance. We utilized a cohort of 431 patients with stage 0–IV CRC who underwent surgery without preoperative treatment. GALNT5 staining was consistently restricted to the malignant epithelial compartment and showed variable cytoplasmic intensity among tumors, often with a perinuclear granular pattern consistent with Golgi-localized glycosyltransferases (Fig. 5A–I; Supplementary Fig. S10). GALNT5-positive tumor cells were frequently enriched at the invasive front and within tumor budding foci (small clusters of tumor cells) (Fig. 5G–I), in line with the invasive and migratory phenotypes observed in vitro. In contrast, non-neoplastic epithelial cells showed only faint staining (Fig. 5C, F), and no appreciable staining was observed in stromal or immune compartments (Fig. 5A–I). The GALNT5 H-score was significantly higher in CRC than in adjacent non-tumor mucosa (Fig. 5J), corroborating the malignant epithelial cell-intrinsic expression of GALNT5 suggested by our single-cell and cell-type-specific transcriptomic analyses (Figs. 1 and 2).

Based on H-score, tumors were classified as GALNT5-High (H-score 8–12; *n* = 83; 19.3%) or GALNT5-Low (0–7; *n* = 348; 80.7%). As shown in Table 1, GALNT5-High tumors were significantly associated with greater depth of tumor invasion (*p* = 0.0004) and more advanced pathological stage (*p* = 0.0311), and showed non-significant trends toward venous invasion (*p* = 0.0566) and lymph node metastasis (*p* = 0.0760). No significant associations were observed with age, sex, tumor location, histological type, lymphatic invasion, or MMR/MSI status. Consistent with these clinicopathological correlations, patients with GALNT5-High CRC had significantly poorer OS than those with GALNT5-Low CRC in patients with Stage I–IV CRC (*n* = 414; *p* = 0.0149; Fig. 5K). In multivariable Cox models, GALNT5-High remained independently associated with worse OS (adjusted HR, 1.63; 95%CI, 1.01–2.56) (Table 2). Given the distinct biology and generally favorable prognosis of dMMR/MSI-H tumors, we further evaluated the prognostic impact of GALNT5 expression in pMMR/MSS cases (*n* = 373). In this subgroup, GALNT5-High remained significantly associated with poorer OS (*p* = 0.0049; Fig. 5L).

**Table 1:** Clinicopathological characteristics of patients with stage 0–IV colorectal cancer according to GALNT5 expression by IHC.

	Total *n* = 431	GALNT5-High *n* = 83 (19.3%)	GALNT5-Low *n* = 348 (80.7%)	*P*
Age				0.4645
Mean ± SD	68.9 ± 11.1	69.7 ± 10.5	68.7 ± 11.3	
Sex				0.5383
Male	245 (56.8%)	50 (60.2%)	195 (56.0%)	
Female	186 (43.2%)	33 (39.8%)	153 (44.0%)	
Location				> 0.9999
Right	165 (38.3%)	32 (38.6%)	133 (38.2%)	
Left	266 (61.7%)	51 (61.4%)	215 (61.8%)	
Histological type				0.8285
Well-moderately differentiated	391 (90.7%)	76 (91.6%)	315 (90.5%)	
Poorly differentiated	13 (3.0%)	2 (2.4%)	11 (3.2%)	
Mucinous	24 (5.6%)	5 (6.0%)	19 (5.5%)	
Signet-ring cell	3 (0.7%)	0 (0.0%)	3 (0.8%)	
Tumor invasion				**0.0004**
Tis	17 (3.9%)	1 (1.2%)	16 (4.6%)	
T1	81 (18.8%)	8 (9.6%)	73 (21.0%)	
T2	67 (15.5%)	9 (10.8%)	58 (16.7%)	
T3	148 (34.3%)	34 (41.0%)	114 (32.8%)	
T4	118 (27.4%)	31 (37.3%)	87 (25.0%)	
Lymphatic invasion				0.1757
Absent	243 (56.4%)	41 (49.4%)	202 (58.0%)	
Present	188 (43.6%)	42 (50.6%)	146 (42.0%)	
Venous invasion				0.0566
Absent	121 (28.1%)	16 (19.3%)	105 (30.2%)	
Present	310 (71.9%)	67 (80.7%)	243 (69.8%)	
Lymph node metastasis				0.0760
Absent	272 (63.1%)	45 (54.2%)	227 (65.2%)	
Present	159 (36.9%)	38 (45.8%)	121 (34.8%)	
Stage				**0.0311**
Stage 0	17 (3.9%)	1 (1.2%)	16 (4.6%)	
Stage I	122 (28.3%)	16 (19.3%)	106 (30.5%)	
Stage II	120 (27.8%)	26 (31.3%)	94 (27.0%)	
Stage III	121 (28.1%)	30 (36.1%)	91 (26.1%)	
Stage IV	51 (11.8%)	10 (12.0%)	41 (11.8%)	
MMR/MSI status				0.0968
dMMR/MSI-H	41 (9.5%)	12 (14.5%)	29 (8.3%)	
pMMR/MSS	390 (90.5%)	71 (85.5%)	319 (91.7%)	

Significant values are in bold.

SD, standard deviation; dMMR/MSI-H, mismatch repair-deficient/microsatellite instability–high; pMMR/MSS, mismatch repair-proficient/microsatellite stable.

**Table 2:** Univariable and multivariable Cox proportional hazard models for overall survival in stage I–IV colorectal cancer.

	Univariable			Multivariable		
	HR	95% CI	*P*	HR	95% CI	*P*
Age						
Continuous	1.00	0.98–1.02	0.869			
Sex						
Male versus female	0.87	0.57–1.32	0.522			
Tumor location						
Right versus Left	1.27	0.83–2.01	0.283			
Histological type						
Wel/Mod versus Por/Muc	1.88	0.97–3.32	**0.042**	1.67	0.86–2.95	0.101
Stage						
I–II versus III–IV	3.87	2.49–6.17	**< 0.0001**	3.73	2.40–5.95	**< 0.0001**
MMR/MSI status						
pMMR/MSS versus dMMR/MSI-H	0.42	0.13–1.01	0.092			
GALNT5						
Low versus High	1.76	1.09–2.76	**0.016**	1.63	1.01–2.56	**0.038**

Significant values are in bold.

HR, hazard ratio; CI, confidence interval; Wel/Mod, well and moderately differentiated adenocarcinoma; Muc, mucinous adenocarcinoma; Por, poorly differentiated adenocarcinoma; dMMR/MSI-H, mismatch repair-deficient/microsatellite instability–high; pMMR/MSS, mismatch repair-proficient/microsatellite stable.

## Discussion

The “mesenchymal” CMS4 subtype of CRC has the poorest prognosis and is characterized by a TGFβ-high, stroma-rich transcriptional profile with elevated EMT-associated signatures^6^. However, bulk EMT signals in CMS4 largely reflect CAF/stromal transcription rather than epithelial-intrinsic EMT, which has hindered identification of tumor cell-intrinsic therapeutic targets^10–12^. Here, using single-cell transcriptomic filtering with validation in cell type-specific datasets and IHC for GALNT5, we delineated a CMS4 epithelial-intrinsic glycosyltransferase panel that minimizes CAF/immune confounding. GALNT5 emerged as a core component of this signature and an independent predictor of poor prognosis, providing novel epithelial-focused insights into the aggressive biology of CMS4 CRC.

Aberrant glycosylation is a hallmark of cancer that reshapes cell-surface glycans and regulates signaling, adhesion, and cell-cell interaction, thereby influencing EMT and metastatic potential^13^. Our nine-gene CMS4-epithelial/glyco signature includes enzymes involved in both N-linked and O-linked glycosylation, several of which (e.g., MGAT5, FUT3, FUT6) have established EMT- and tumor-promoting roles^14–16,20,21,23^. Among the nine genes, GALNT5 was upregulated under GSK3 inhibition-associated EMT-like changes in CRC cells. The observed induction of Snail, together with decreased E-cadherin and morphological changes, is more consistent with EMT-like epithelial–mesenchymal plasticity or a partial EMT state than with a complete mesenchymal conversion^32,33^. Because SB415286 is a GSK3 inhibitor, the upregulation of GALNT5 should be interpreted cautiously as an epithelial-intrinsic EMT-like response associated with GSK3 inhibition, potentially including Wnt/β-catenin-related effects, rather than as evidence of definitive canonical EMT induction. Nevertheless, GALNT5 knockdown reduced proliferation, invasion, and migration despite minimal or inconsistent changes in canonical EMT markers or AKT/ERK signaling. Thus, in our CRC models with impaired canonical TGFβ signaling, GALNT5 may contribute to aggressive behavior associated with EMT-like epithelial plasticity without being required for a complete EMT program, and may function as an effector rather than an upstream transcriptional driver of EMT.

Our findings are consistent with prior work showing that loss of the tumor-suppressive microRNA miR-196b-5p derepressed GALNT5 and enhanced CRC migration and metastasis without overt EMT marker changes^34^. This miR-196b-5p/GALNT5 axis correlated with adverse OS, aligning with our finding that IHC-defined GALNT5-High tumors had poorer OS. A tumor-promoting, adverse prognostic role for GALNT5 has also been reported in other gastrointestinal cancers, including pancreatic ductal adenocarcinoma (PDAC) and cholangiocarcinoma (CCA), where GALNT5 has been linked to increased proliferation, invasion, and migration and, in some settings, EMT and EGFR-AKT/ERK activation^31,35–37^. Additionally, in PDAC, GALNT5-driven glycosylation has been associated with NOTCH-mediated chemoresistance, ferroptosis suppression, and immune modulation^35,36^. Collectively, these data suggest that GALNT5 may contribute to malignant phenotypes through multiple mechanisms, including those dependent on, and independent of, EMT program or AKT/ERK signaling. Although we did not observe robust activation of the AKT/ERK pathways in our CRC models, GALNT5 may promote invasion by O-glycosylating cell-surface receptors. Because GALNT enzymes initiate mucin-type O-glycosylation on proteins governing cell-matrix interactions, such as integrins and CD44^13,17,38^, GALNT5-mediated modification may alter receptor conformation or clustering to enhance cellular motility without a complete transcriptional EMT switch. Defining GALNT5 substrates in CMS4 CRC will require glycoproteomic approaches and may clarify whether GALNT5 supports a “partial EMT” phenotype in which invasion/migration capacity is accompanied by retained epithelial features.

Beyond GALNT5, enzymes in the CMS4-epithelial/glyco signature may promote malignancy through distinct, partly parallel mechanisms. FUT3 and FUT6 can foster TGFβ-driven EMT by fucosylating the TGFβ type I receptor, thereby promoting invasion and migration^15,16,23^. They also increase sialyl-Lewis antigens (sLe^x^ and sLe^a^/CA19-9), which facilitate tumor cell adhesion to endothelial E-selectin and metastatic dissemination. MGAT5 promotes EMT by stabilizing receptors, including TGFβ receptors I/II and EGFR, while its N-glycosylation can destabilize and mislocalize E-cadherin, facilitating detachment and migration. MGAT5 can also promote N-glycosylation of the tight junction protein ZO-1, leading to its enhanced ubiquitination and subsequent degradation^14,16,20^. Together, these examples highlight that glycosyltransferases in our signature can coordinately modulate adhesion, signaling, and microenvironmental interactions; their concerted upregulation in CMS4 tumor cells may drive epithelial plasticity, progression, and metastasis, contributing to poor prognosis.

From a translational perspective, epithelial-intrinsic glycosylation programs may aid prognostic stratification and therapeutic targeting in CRC. The association of high GALNT5 protein expression with adverse clinicopathological features and worse OS suggests that GALNT5 could serve as a biomarker to identify a subgroup of aggressive CRCs, particularly within pMMR/MSS tumors. Accordingly, IHC assessment of GALNT5 in resected specimens may help guide postoperative risk-adapted therapy and surveillance. GALNT5 also represents a potential therapeutic target. Defining GALNT5 substrates and downstream signaling pathways will be important for developing targeted interventions. Future therapeutic strategies may include inhibition of GALNT5 enzymatic activity, blockade of GALNT5-associated pathways, or immunotherapies directed against GALNT5-mediated glycan structures. Because GALNT5 expression was relatively enriched in malignant epithelium in our paired whole-section analysis, it may offer a potentially selective therapeutic target. Although the Human Protein Atlas shows GALNT5 staining in normal colonic epithelium, this apparent difference may reflect variation in IHC protocols and analytical dynamic range.

This study has several limitations. First, our functional analyses relied on two CRC cell lines and therefore do not capture stromal/immune interactions that may shape glycosylation-dependent phenotypes in vivo. Moreover, most established CRC cell lines, including the two used here, harbor inactivating alterations in the TGFβ pathway^2^, thereby limiting direct in vitro modeling of canonical TGFβ-driven processes such as EMT. Indeed, canonical TGFβ responsiveness of CRC epithelial cells is often attenuated by loss-of-function alterations in TGFβ pathway components, and even CRC models with an intact pathway may show heterogeneous responses without overt EMT^2^. Alternative strategies, including patient-derived organoids and/or xenograft models will be needed to test whether GALNT5 targeting suppresses metastatic dissemination. Second, the molecular mechanisms remain unsolved, as we did not identify the glycoprotein substrates through which GALNT5 exerts tumor-promoting effects. Glycoproteomic approaches will be required to define GALNT5-dependent O-glycosylation targets and their functional consequences. Third, because the prognostic association of GALNT5 was derived from a single-institution cohort, validation in independent cohorts is warranted.

In conclusion, we defined a CMS4 epithelial-intrinsic glycosyltransferase signature by leveraging single-cell transcriptomics to minimize stromal confounding. Within this panel, GALNT5 was induced during EMT-like changes and contributed to proliferation, invasion, and migration in CRC cells. High GALNT5 protein expression was independently associated with poorer survival, including in pMMR/MSS tumors. These findings implicate GALNT5 or its downstream glycosylation events as candidate targets for therapeutic intervention in CMS4 CRC and support IHC-based GALNT5 assessment for prognostic risk stratification.

## Methods

### Transcriptomic data analysis

All transcriptomic datasets utilized in this study are publicly available, and preprocessed expression values were used as provided. If a gene was represented by multiple probes, only the probe with the highest mean expression was used. Two scRNA-seq datasets of CRC (GSE132465 and GSE146771), as well as two microarray datasets of FACS-purified cell populations (GSE39395 and GSE39396), were downloaded from the Gene Expression Omnibus (GEO) database (https://www.ncbi.nlm.nih.gov/geo/)^4,18,28,39^. For GSE132465, we used the processed files GSE132465_GEO_processed_CRC_10X_cell_annotation.txt and GSE132465_GEO_processed_CRC_10X_natural_log_TPM_matrix.txt. For GSE146771, we used CRC.Leukocyte.Smart-seq2.TPM.txt and CRC.Leukocyte.Smart-seq2.Metadata.txt. Published cell annotations or metadata were used as provided. No additional processing of raw count data, reclustering, or reannotation was performed. We also obtained two bulk RNA-seq datasets, including TCGA and AC-ICAM (an Atlas and Compass of Immune-Cancer-Microbiome interactions), through cBioPortal (https://www.cbioportal.org/)^40–42^. For calculation of multi-gene signature scores for each sample or cell fraction, expression values of individual genes were first standardized as Z-scores within each dataset. The pan-cancer EMT signature was calculated as the mean expression of the 52 mesenchymal genes minus the mean expression of the 25 epithelial genes from the published 77-gene signature^29^. The CMS4-epithelial/glyco signature was calculated as the mean expression of the nine genes identified in this study.

### Selection of epithelial CMS4-enriched glycosylation genes from scRNA-seq data

We utilized a panel of 188 glycosyltransferase genes derived from 10 KEGG gene sets^43^ in the Molecular Signature Database (MSigDB v2023.2.Hs), as defined in our recent work^19^. To identify gene candidates specifically enriched in CMS4 tumor epithelium, we applied a two-step filtering scheme to the GSE132465 scRNA-seq dataset^18,39^, using the published cell annotation file and natural log TPM matrix described above. Cell populations were partitioned into tumor epithelial cells (*n* = 17,469), immune cells (*n* = 27,080), stromal cells (*n* = 2736), and normal colonic epithelial cells (*n* = 1070). Screening metrics for the predefined 188-gene glycosyltransferase panel were calculated directly from the processed expression matrix. “Expressing cells” were defined as cells with non-zero expression values. Step-1 (epithelial CMS4 enrichment): genes (i) expressed in > 15% of all tumor epithelial cells, (ii) with log_2_ fold-change (log_2_FC) > 0.4 for CMS4 versus CMS1–3 tumor epithelial cells, and (iii) with log_2_FC > 0.4 for CMS4 tumor epithelial versus normal epithelial cells. Step-2 (non-epithelial depletion): among Step-1 genes, we retained those expressed in < 15% of all immune cells and < 15% of all stromal cells. The two-step procedure yielded an initial set of 19 genes at Step-1 and a refined set of 9 genes at Step-2. These thresholds were used as screening thresholds to capture recurrent glycosyltransferase signals enriched in CMS4 tumor epithelium while limiting genes driven by sparse expression in scRNA-seq data. The two-step procedure yielded an initial set of 19 genes at Step-1 and a refined set of 9 genes at Step-2. The complete results for all 188 glycosyltransferase genes are provided in Supplementary Table S1.

### Cell culture and reagents

CRC cell lines, SW480 and HCT116, were purchased from the American Type Culture Collection (Manassas, VA) and RIKEN BioResource Center (Ibaraki, Japan), respectively. Cells were cultured in Dulbecco’s Modified Eagle’s Medium (DMEM) supplemented with 10% fetal bovine serum (FBS) (Biological Industries, Kibbutz Beit Haemek, Israel) and penicillin–streptomycin solution (Thermo Fisher Scientific, Waltham, MA). EMT induction was performed using GSK3 inhibitor, SB415286 (S3567_SIGMA; Sigma-Aldrich, St. Louis, MO), as described previously^44^. Briefly, cells were treated with 10 to 50 µmol/L of SB415286 for 72 h and were used for subsequent experiments. For TGFβ stimulation, cells were treated with recombinant human TGF-β1 (7754-BH; R&D Systems), reconstituted with Reconstitution Buffer 4 (RB04; R&D Systems), at a final concentration of 10 or 50 ng/mL for 72 h. For knockdown experiments, siRNA oligonucleotides against GALNT5 or a scramble control (Ambion Silencer^®^ Select, s22157, s22158 and Negative Control #1; Thermo Fisher Scientific) were transfected using Lipofectamine RNAiMAX Reagent according to the manufacturer’s instructions.

### Reverse transcription-quantitative PCR (RT-qPCR)

Total RNA isolation and RT-qPCR were performed as described previously^45^, with specific primers and probes for B3GNT5 (Assay ID: Hs.PT.58.25390148), *C1GALT1* (Hs.PT.58.4490602), *FUT2* (Hs.PT.58.38692902), *FUT3* (Hs.PT.58.27790104), *FUT6* (Hs.PT.58.40906728), *GALNT3* (Hs.PT.27773679), *GALNT5* (Hs.PT.58.2594239), *GCNT3* (Hs.PT.58.2760328), *MGAT5* (Hs.PT.58.4758371), and *GAPDH* (Hs.PT.58.22214836) (Integrated DNA Technologies, Coralville, IA).

### Western blot analysis

Western blot analysis was performed as previously described^19^, using antibodies against E-Cadherin (24E10) (#3195; Cell Signaling Technology; 1:1000), Vimentin (D21H3) (#5741; Cell Signaling Technology; 1:1000), Snail (C15D3) (#3879; Cell Signaling Technology; 1:1000), phospho-Akt (Ser473) (D9E) (#4060; Cell Signaling Technology; 1:2000), Akt (C67E7) (#4691; Cell Signaling Technology; 1:1000), phosphor-p4/42 MAPK (ERK1/2) (Thr202/Tyr202) (D13.12.4E) (#4370; Cell Signaling Technology; 1:2000), p44/42 MAPK (ERK1/2) (137F5) (#4695; Cell Signaling Technology; 1:1000), and β-actin (sc-69879; Santa Cruz Biotechnology, Dallas, TX; 1:5000). Immunoreactive proteins were detected using ImageQuant LAS 4000 mini (FUJIFILM, Tokyo, Japan) with ECL prime Western Blotting Detection Reagent (Cytiva, Tokyo, Japan), and quantified using Image J software. The original, unprocessed blot images are shown in Supplementary Fig. S11.

### Cell proliferation assay

Cellular proliferation was measured using the Cell Counting Kit-8 (CCK-8, DOJINDO, Kumamoto, Japan) according to the manufacturer’s instructions. After 24 h of siRNA transfection, cells were seeded in 96-well plates at 5.0 × 10³ cells/well, and incubated for 24, 48 and 72 h.

### Wound healing assay

The migratory capacity was investigated using the Culture-Insert 2 Well (ibidi, Munich, Germany) according to the manufacturer’s instructions. Briefly, after 24 h of siRNA treatment, 70 µL of 7 × 10⁵ cells/mL suspensions were incubated for 24 h. After removing the culture insert, cells were incubated for 48 h.

### Transwell invasion assay

Invasion assays were performed using 8-µm pore size transwell chambers pre-coated with Matrigel (354480, Corning, NY). Cells were harvested 24 h after siRNA transfection, resuspended in serum-free medium, and seeded into the upper chamber at a density of 2.0 × 10⁵ cells/well. The lower chamber was filled with culture medium supplemented with 10% FBS as a chemoattractant. After incubation for 24 h, cells that invaded through the Matrigel to the lower surface were fixed with 70% methanol and stained with Giemsa solution. The number of invaded cells was quantified by counting five randomly selected fields.

### Patient samples

We enrolled consecutive patients with primary colorectal adenocarcinoma who underwent surgical resection at Fukushima Medical University Hospital between 2014 and 2021. After excluding patients who received preoperative chemotherapy or radiotherapy, we used formalin-fixed paraffin-embedded (FFPE) whole tissue sections obtained from 431 patients with stage 0–IV CRC. Tumors were classified according to the Japanese Classification of Colorectal, Appendiceal, and Anal Carcinoma (9th Edition)^46^. Adjacent normal mucosae from 417 sections were also available for evaluation. Clinical information was retrospectively obtained by reviewing medical records, with the last follow-up in August 2025. Overall survival (OS) was defined as the time from the date of surgery to the date of death from any cause. For all cases, mismatch-repair or microsatellite instability (MMR/MSI) status was available based on either or both MSI testing and IHC for MMR proteins (MLH1, MSH2, MSH6 and PMS2), as determined and described in our recent studies^19,47^. This study was conducted in compliance with the declaration of Helsinki. The study was approved by the Institutional Review Board of Fukushima Medical University (REC2024-041), and samples were obtained with the patients’ informed consent.

### IHC and assessment of staining

IHC for GALNT5 was performed on 4-µm FFPE sections as previously described^19^, using primary rabbit polyclonal anti-GALNT5 antibody (HPA009035; Prestige Antibodies Powered by Atlas Antibodies, Sigma-Aldrich; 1:500). The sections were evaluated independently by two observers who were blinded to all clinical and pathological data. GALNT5 staining in the cytoplasm of epithelial cells was evaluated for H-score. The intensity of staining was scored as 0 (negative), 1 (weak), 2 (moderate), or 3 (strong), and the percentage of positive cells was scored as 1 (0–25%), 2 (26–50%), 3 (51–75%), or 4 (76–100%). The intensity and positivity scores were then multiplied to obtain the GALNT5 H-score (min 0, max 12). Digital images were acquired using a digital slide scanner (NanoZoomer-SQ; Hamamatsu Photonics, Shizuoka, Japan).

### Statistical analysis

Differences in gene expression between the control and treated groups in cell lines (RT-qPCR) were analyzed using multiple unpaired *t*-tests, and the False Discovery Rate (FDR) was controlled using the two-stage step-up procedure of Benjamini, Krieger and Yekutieli, with a Q-value cutoff of 0.05. For comparisons among multiple groups, one-way or two-way analysis of variance (ANOVA) followed by Dunnett’s test for multiple comparisons was performed. Differences in clinicopathological variables between GALNT5-High and -Low groups were analyzed using Fisher’s exact test, the χ^2^ test, the χ^2^ test for trend, or the unpaired *t*-test with Welch’s correction, where appropriate. Cumulative survival was estimated by the Kaplan–Meier method, and differences were analyzed by the log-rank test. Cox proportional hazards regression was used to compute univariable and multivariable hazard ratios (HR) and 95% confidence intervals (CI). Statistical analyses were performed using GraphPad Prism 9 (GraphPad Software, San Diego, CA). Data are represented as mean ± SD, and *p* < 0.05 was considered statistically significant.

## Supplementary Information

Below is the link to the electronic supplementary material.

Supplementary Material 1

Supplementary Material 2

## Data Availability

The public datasets used in this study are available from the GEO database (http://www.ncbi.nlm.nih.gov/geo), or cBioPortal (http://www.cbioportal.org/).
