# Supplementary material for: CMS4 epithelial-intrinsic glycosyltransferase GALNT5 promotes tumor aggressiveness and correlates with poor survival in colorectal cancer
Source: Sci Rep. 2026 Jun 8;16:25985. doi: 10.1038/s41598-026-57104-1 (PMC13486498; doi:10.1038/s41598-026-57104-1)

Supplementary Figure S1

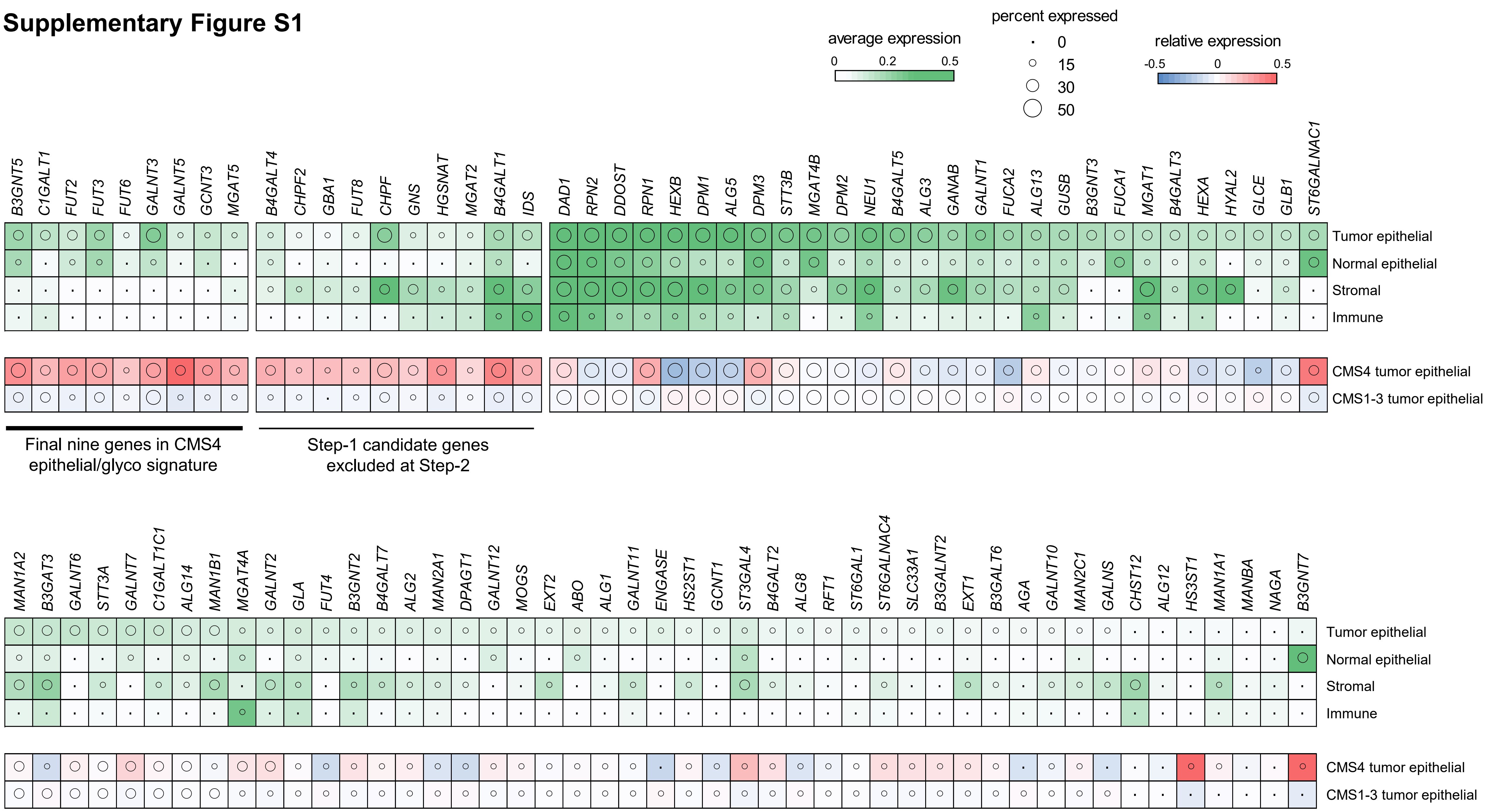

Supplementary Figure S1 Continued

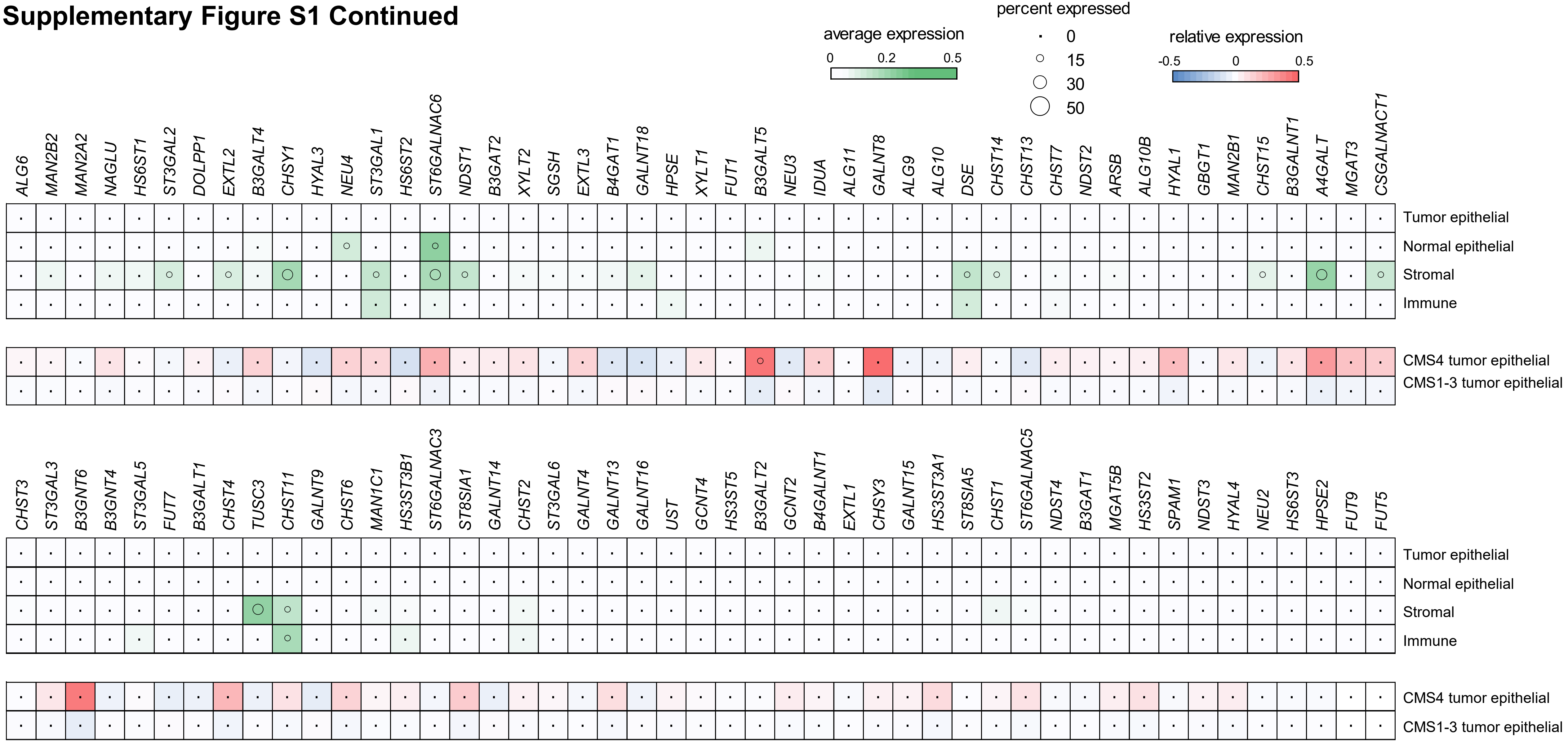

Supplementary Figure S1. scRNA-seq overview of 188 glycosyltransferase genes. Dot plots showing all glycosyltransferase genes analyzed in the GSE132465 scRNA-seq dataset. The final nine genes included in the CMS4 epithelial/glyco signature and 10 Step-1 candidate genes excluded at Step-2 are indicated. Dot color represents average expression level, and dot size represents the percentage of expressing cells. Upper panels show expression across four cell populations: tumor epithelial cells (n = 17,469), normal epithelial cells (n = 1,070), stromal cells (n = 2,736), and immune cells (n = 27,080). Lower panels show relative expression in CMS4 tumor epithelial cells (n = 2,171) versus CMS1–3 tumor epithelial cells (n = 15,298).

Supplementary Figure S2

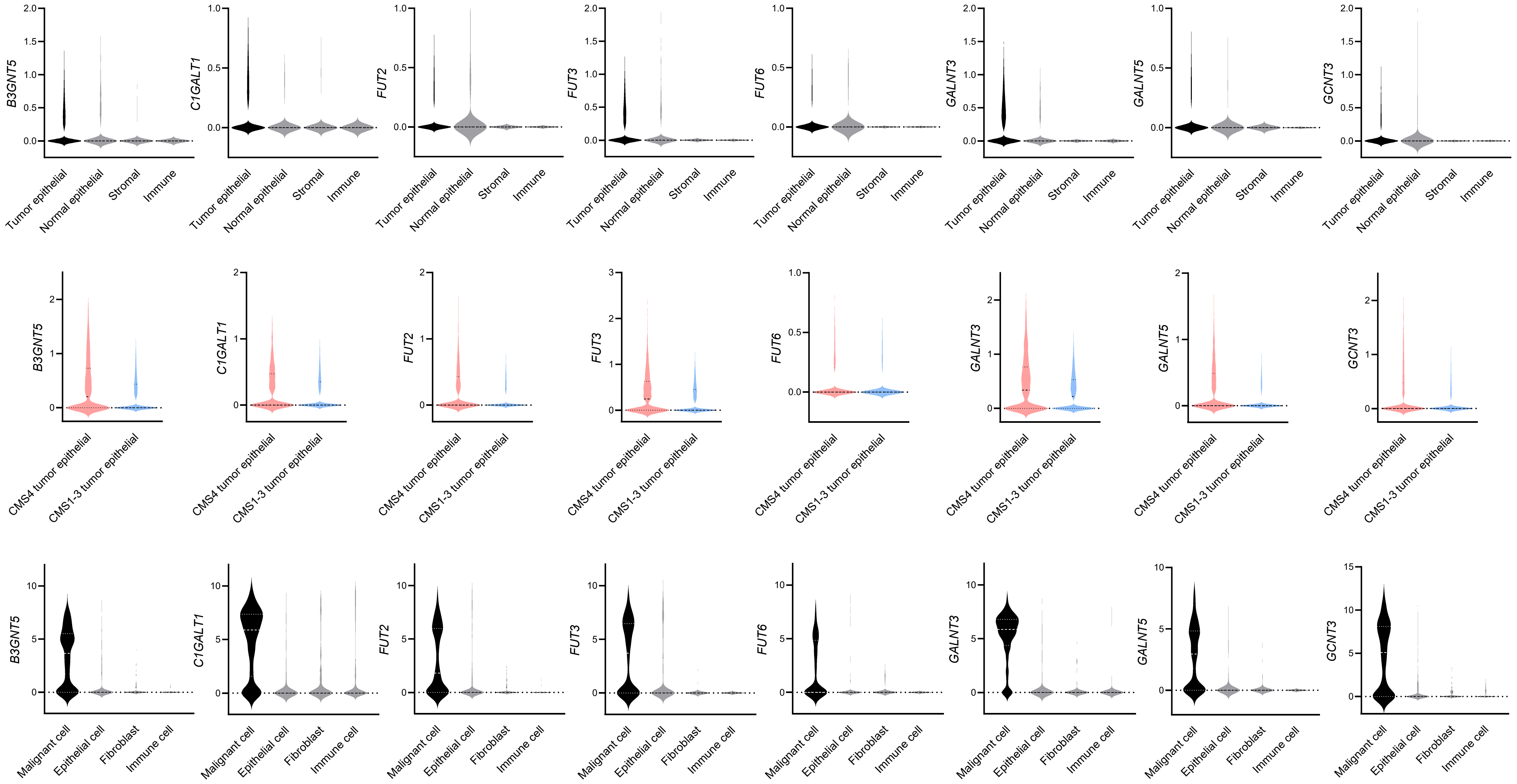

Supplementary Figure S2. Violin plots showing cell-level expression distributions of the nine genes included in the final CMS4 epithelial/glyco signature. Top row, corresponding to Fig. 1 (upper panel), shows expression across tumor epithelial, normal epithelial, stromal, and immune cells in GSE132465. Middle row, corresponding to Fig. 1 (lower panel), shows expression in CMS4 versus CMS1–3 tumor epithelial cells in GSE132465. Bottom row, corresponding to Fig. 2A, shows expression across malignant cells, non-malignant epithelial cells, fibroblasts, and immune cells in the independent scRNA-seq dataset GSE146771.

Supplementary Figure S3

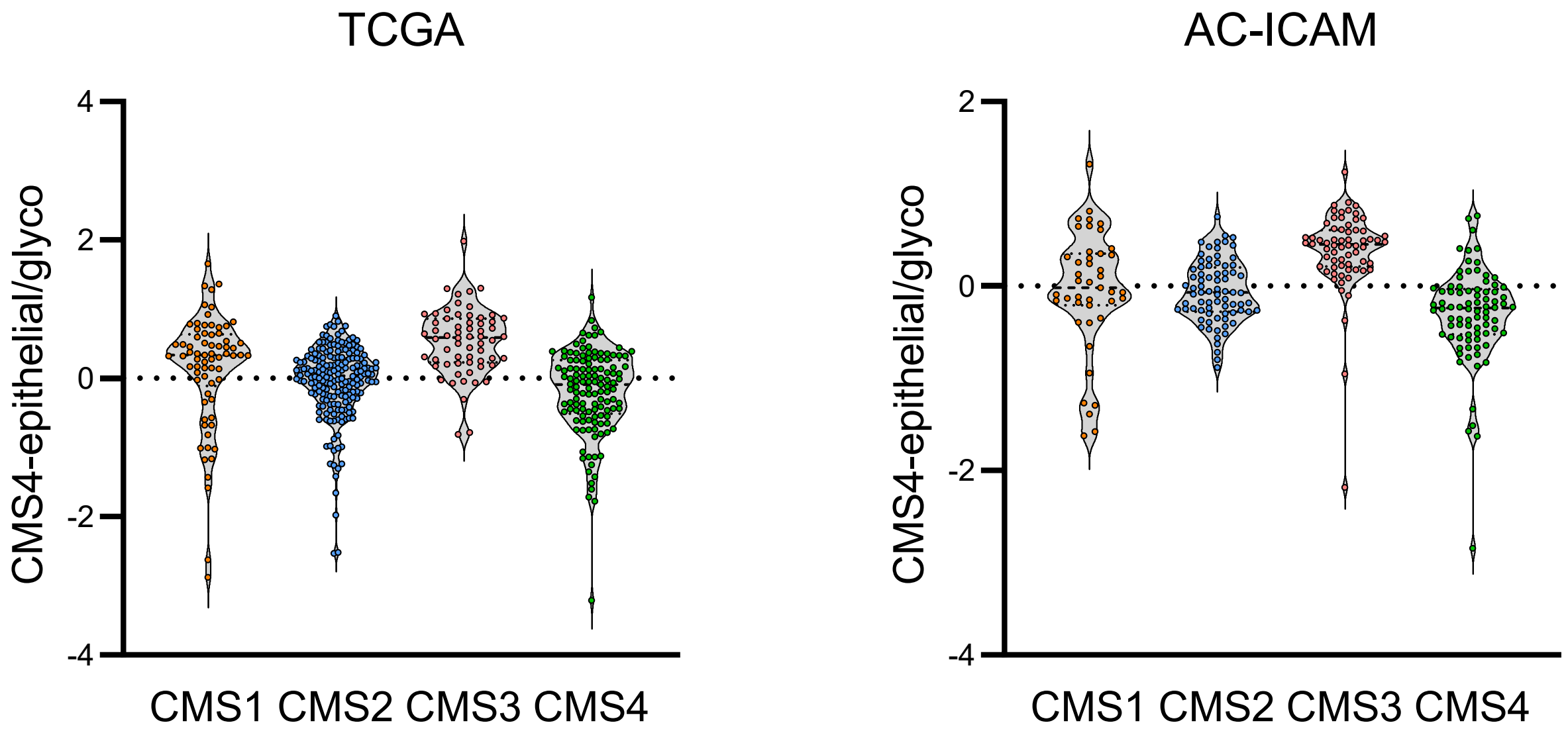

Supplementary Figure S3. Levels of the nine-gene CMS4-epithelial glycosyltransferase signature (CMS4-epithelial/glyco) in bulk transcriptomic datasets (TCGA and AC-ICAM). The signature identified based on single-cell analyses was not selectively increased in bulk CMS4 tumors.

Supplementary Figure S4

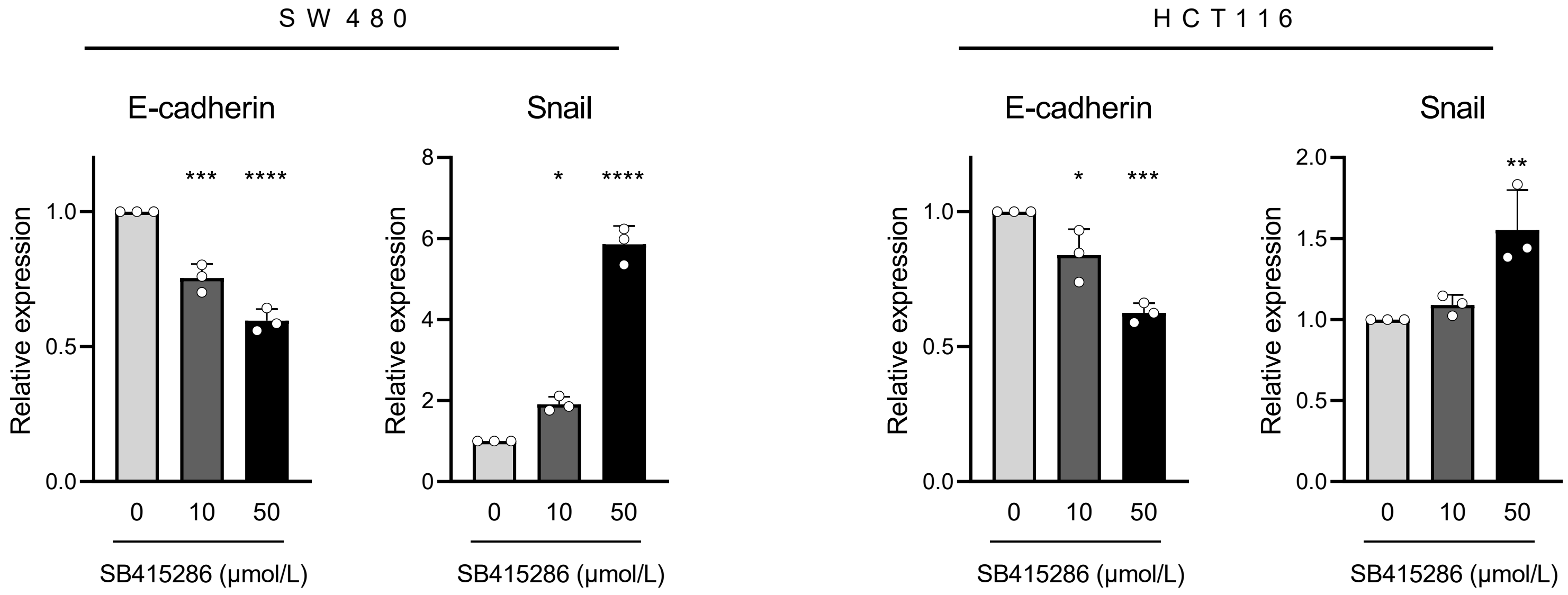

Supplementary Figure S4. Densitometric quantification of E-cadherin and Snail western blot analysis in SW480 and HCT116 cells treated with the GSK3 inhibitor SB415286 or vehicle control. One-way ANOVA followed by Dunnett's multiple-comparisons test. ****P < 0.0001, ***P < 0.001, **P < 0.01, *P < 0.05. versus vehicle control.

Supplementary Figure S5

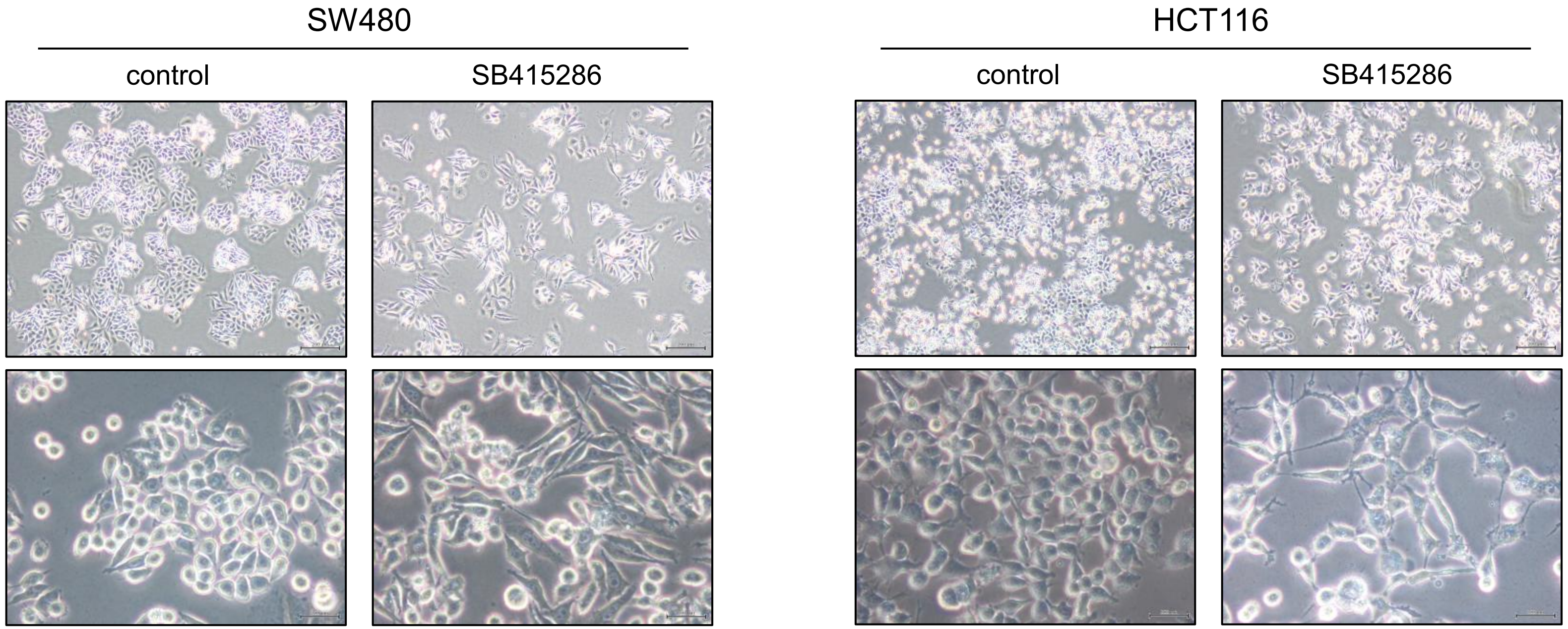

Supplementary Figure S5. Representative low- and high-magnification images showing spindle-like morphological changes following SB415286 treatment in SW480 and HCT116 cells. Images were acquired at ×4 (Upper panels) and ×20 (Lower panels) magnification.

Supplementary Figure S6

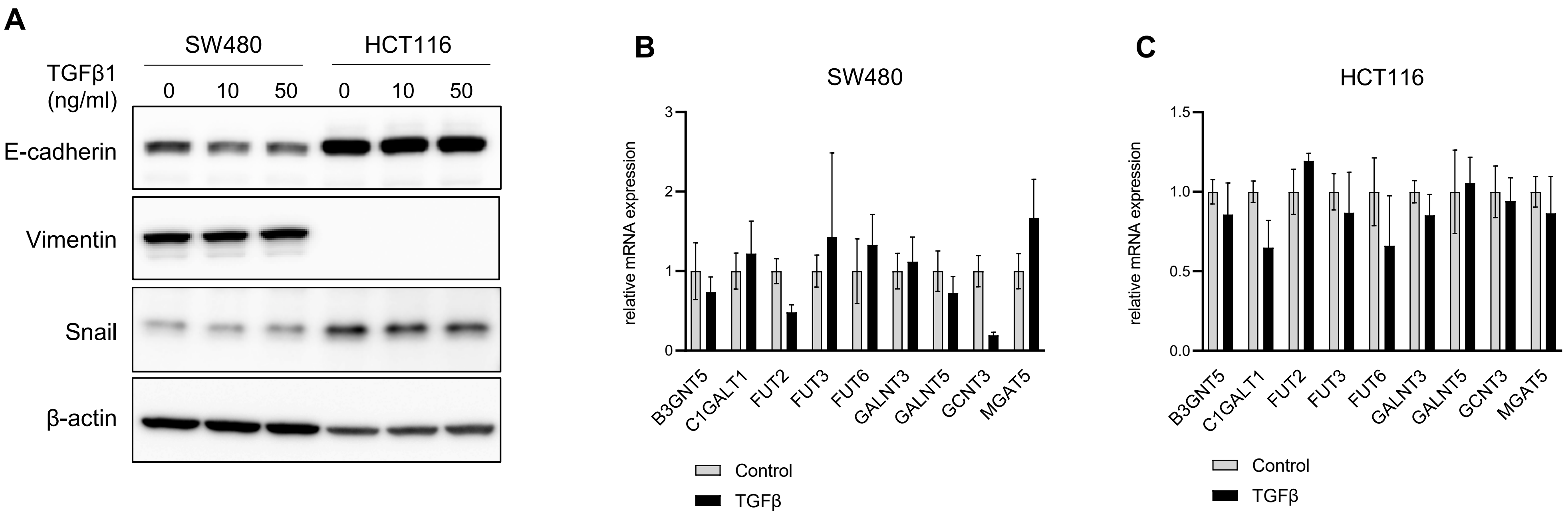

Supplementary Figure S6. HCT116 and SW480 cells treated with TGFβ. (A) Western blot analysis of EMT markers in HCT116 and SW480 cells treated with TGFβ1 or vehicle control. (B,C) RT-qPCR analysis of the nine CMS4-associated glycosyltransferase genes in SW480 (B) and HCT116 (C) cells after TGFβ treatment (10 μmol/L). Data are expressed as fold change relative to controls. Multiple unpaired *t*-test.

Supplementary Figure S7

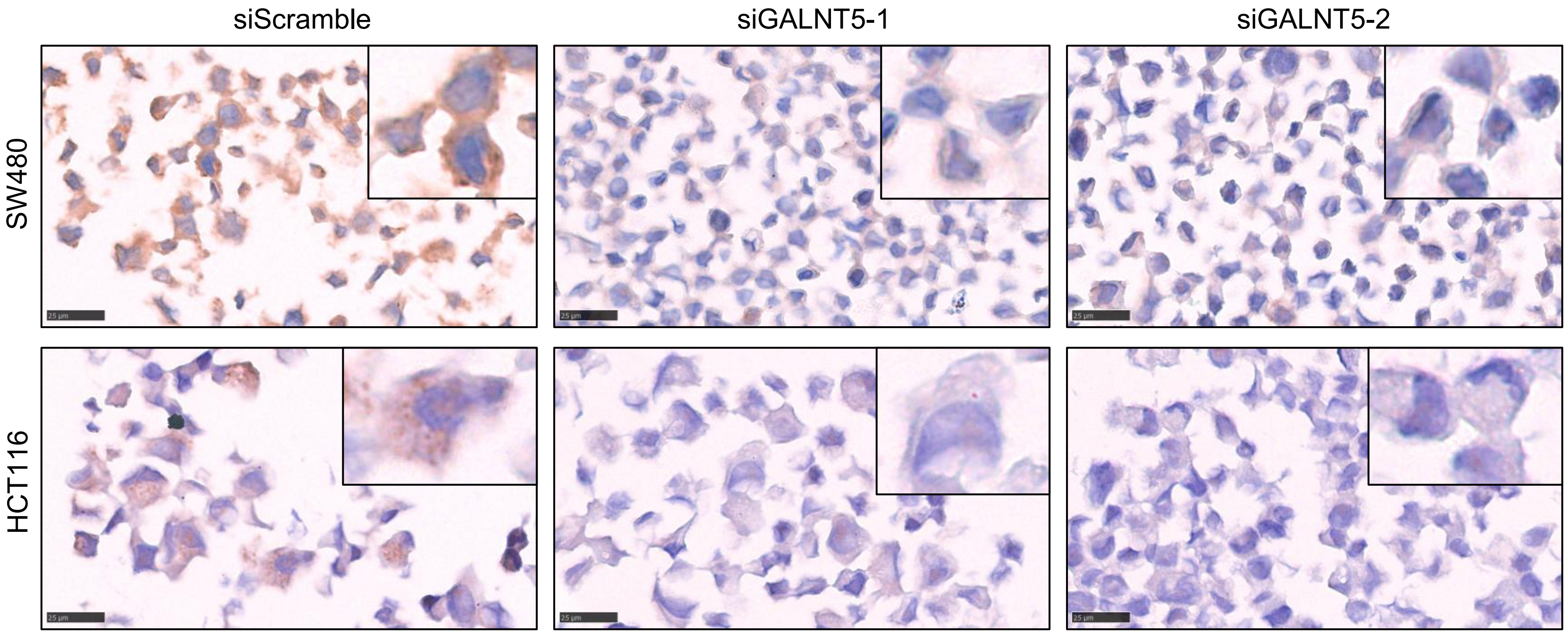

Supplementary Figure S7. IHC showing reduced cytoplasmic GALNT5 protein expression after transfection with two independent siRNAs versus non-targeting control in SW480 and HCT116 cells. Bar = 25μm. Cells were harvested and fixed in 10% formalin and embedded in paraffin, and then used for IHC.

Supplementary Figure S8

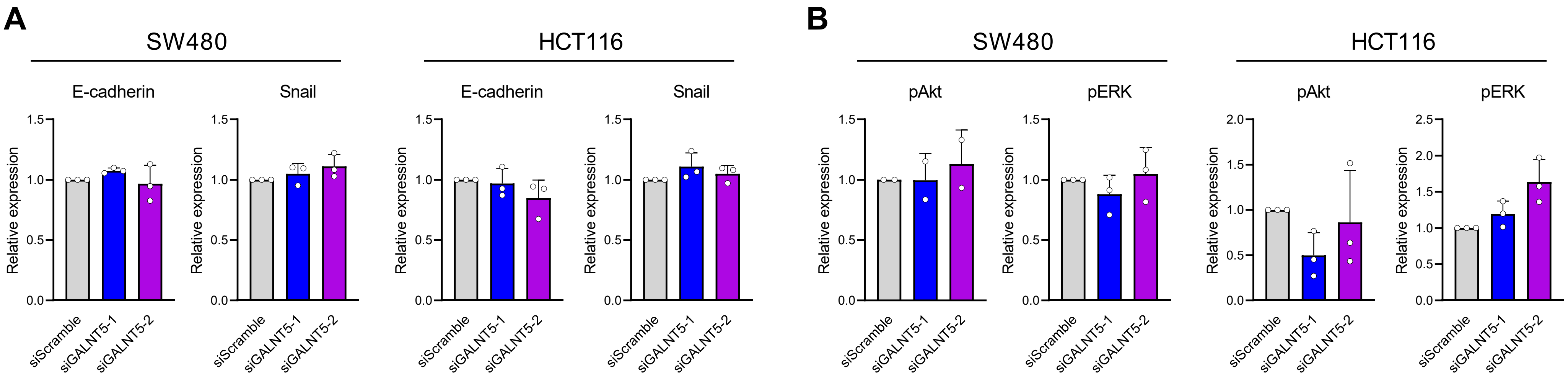

Supplementary Figure S8. Densitometric quantification of western blot analyses for (A) E-cadherin and Snail and (B) pAKT and pERK in SW480 and HCT116 cells after GALNT5 knockdown.

Supplementary Figure S9

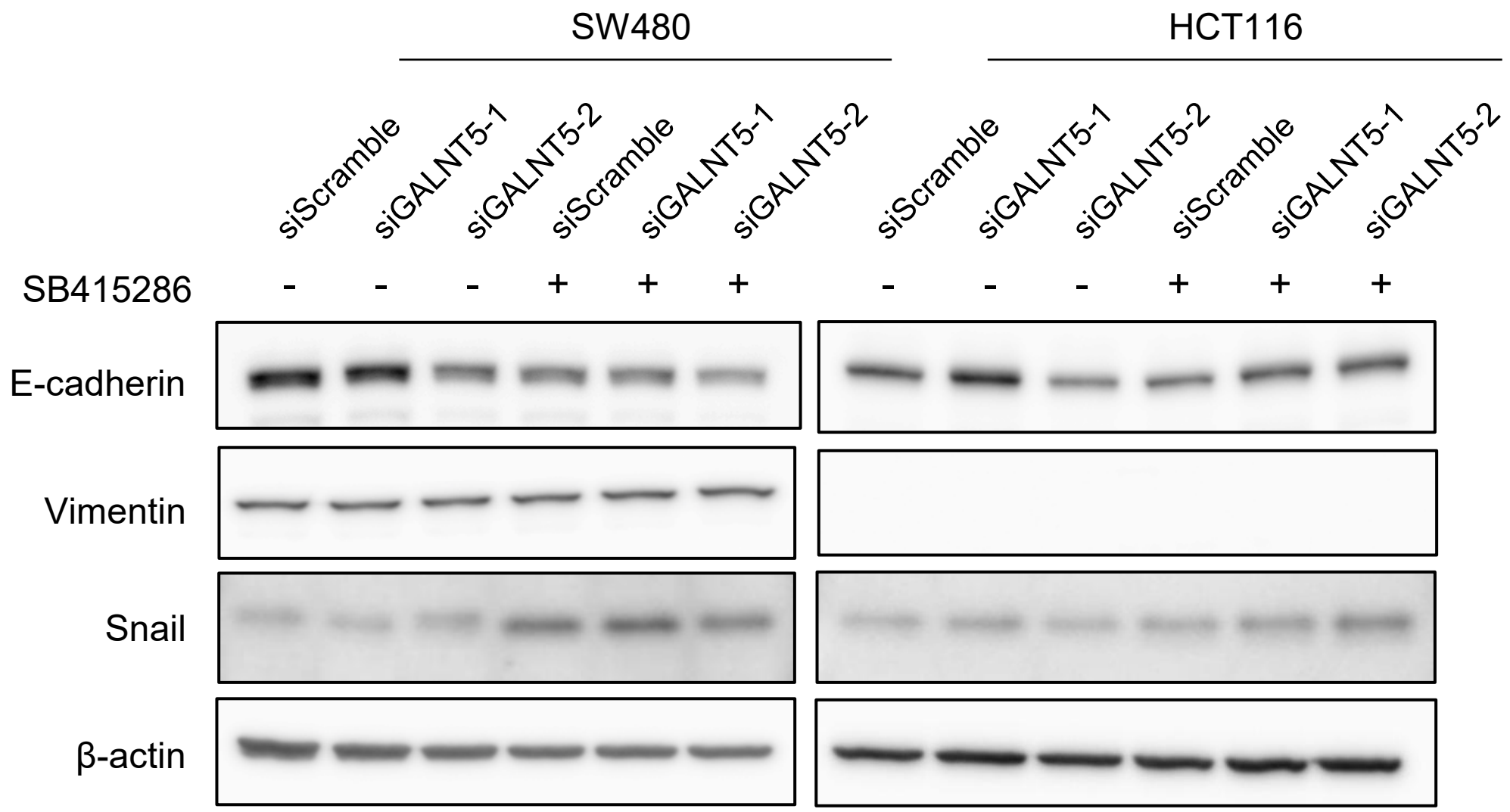

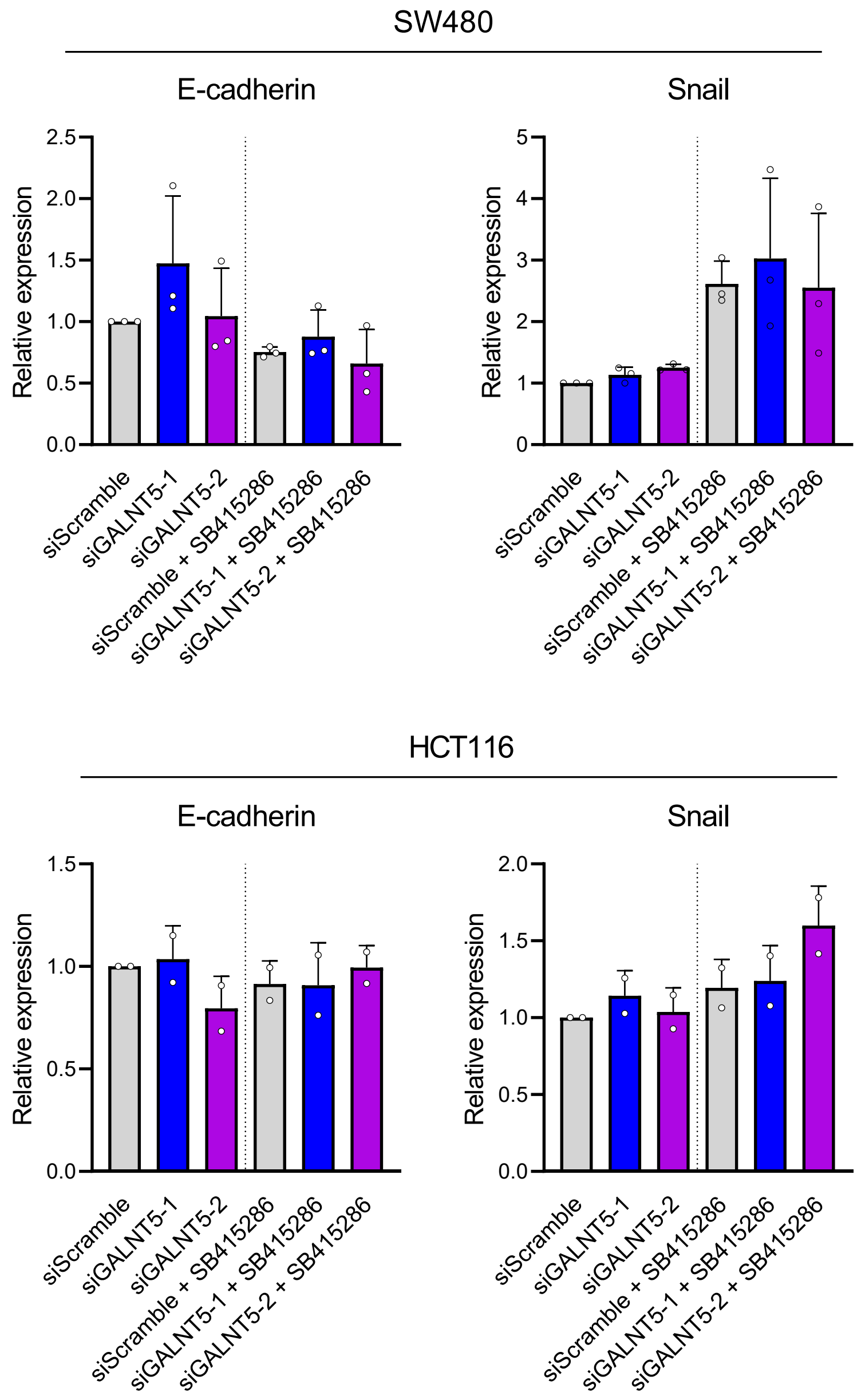

Supplementary Figure S9. Effect of GALNT5 knockdown on EMT marker expression in HCT116 and SW480 cells treated with the GSK3 inhibitor SB415286 or vehicle control.

Supplementary Figure S10

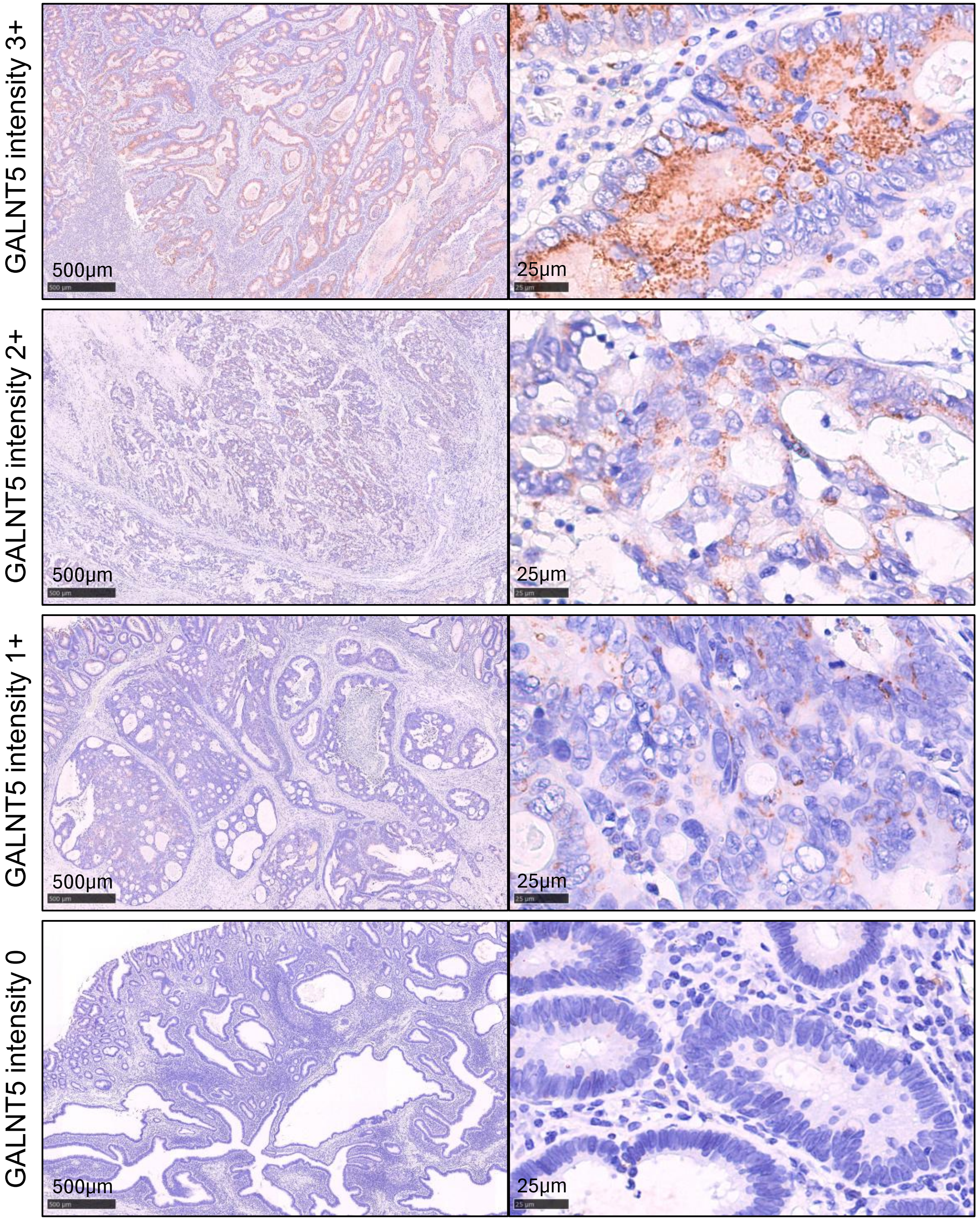

Supplementary Figure S10. IHC for GALNT5 protein expression in CRC. Representative intensities of GALNT5 staining in CRC.

Supplementary Figure S11

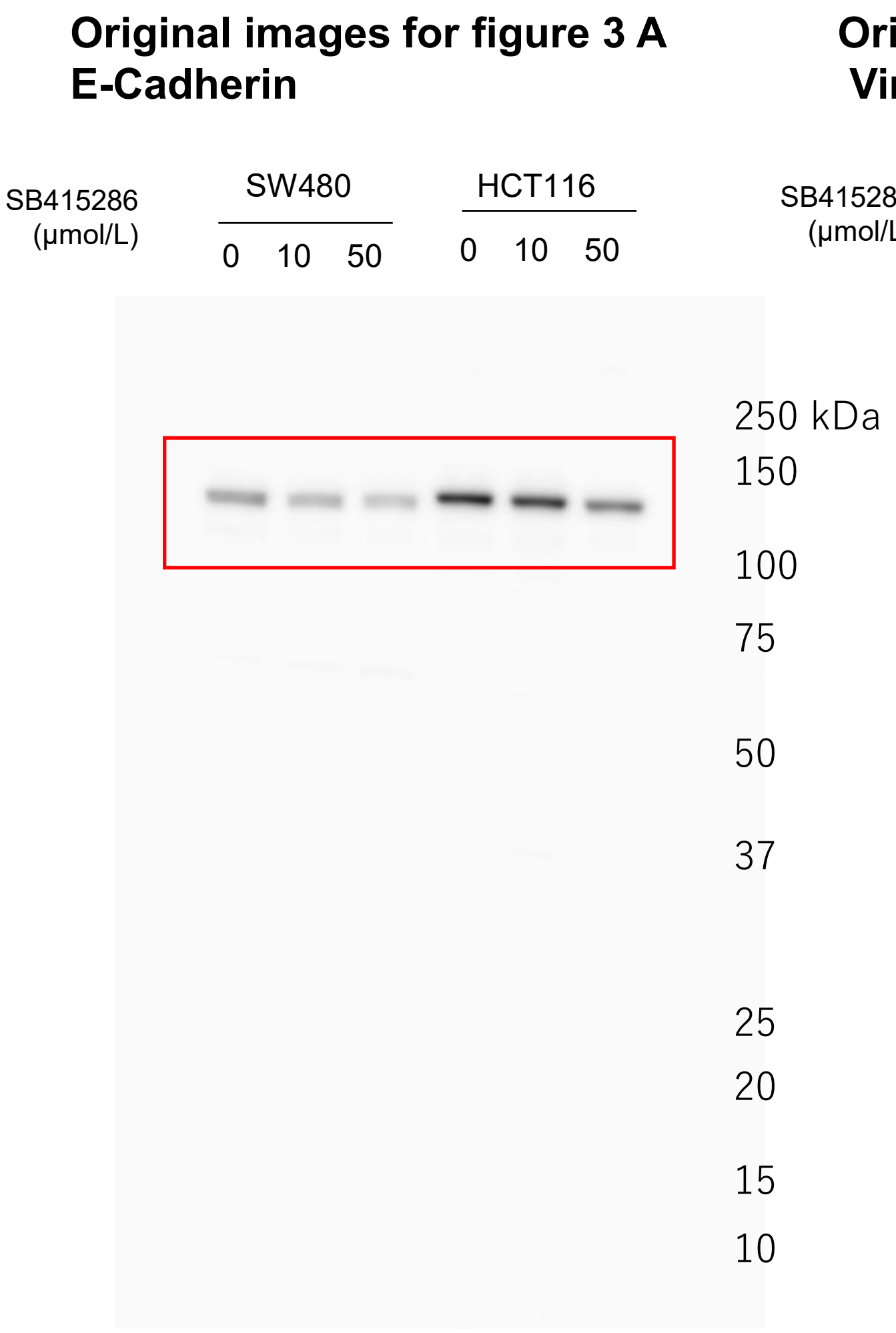

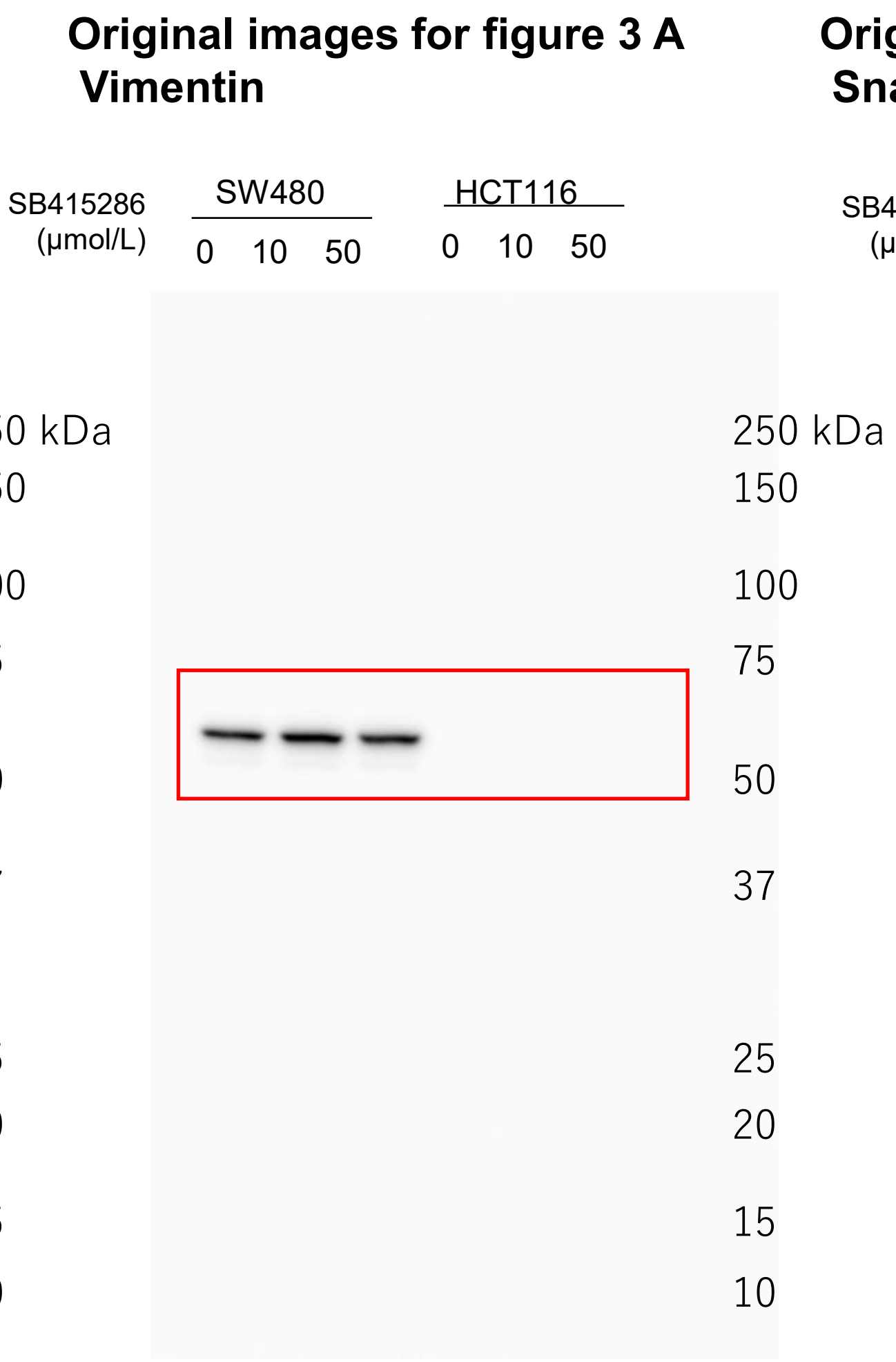

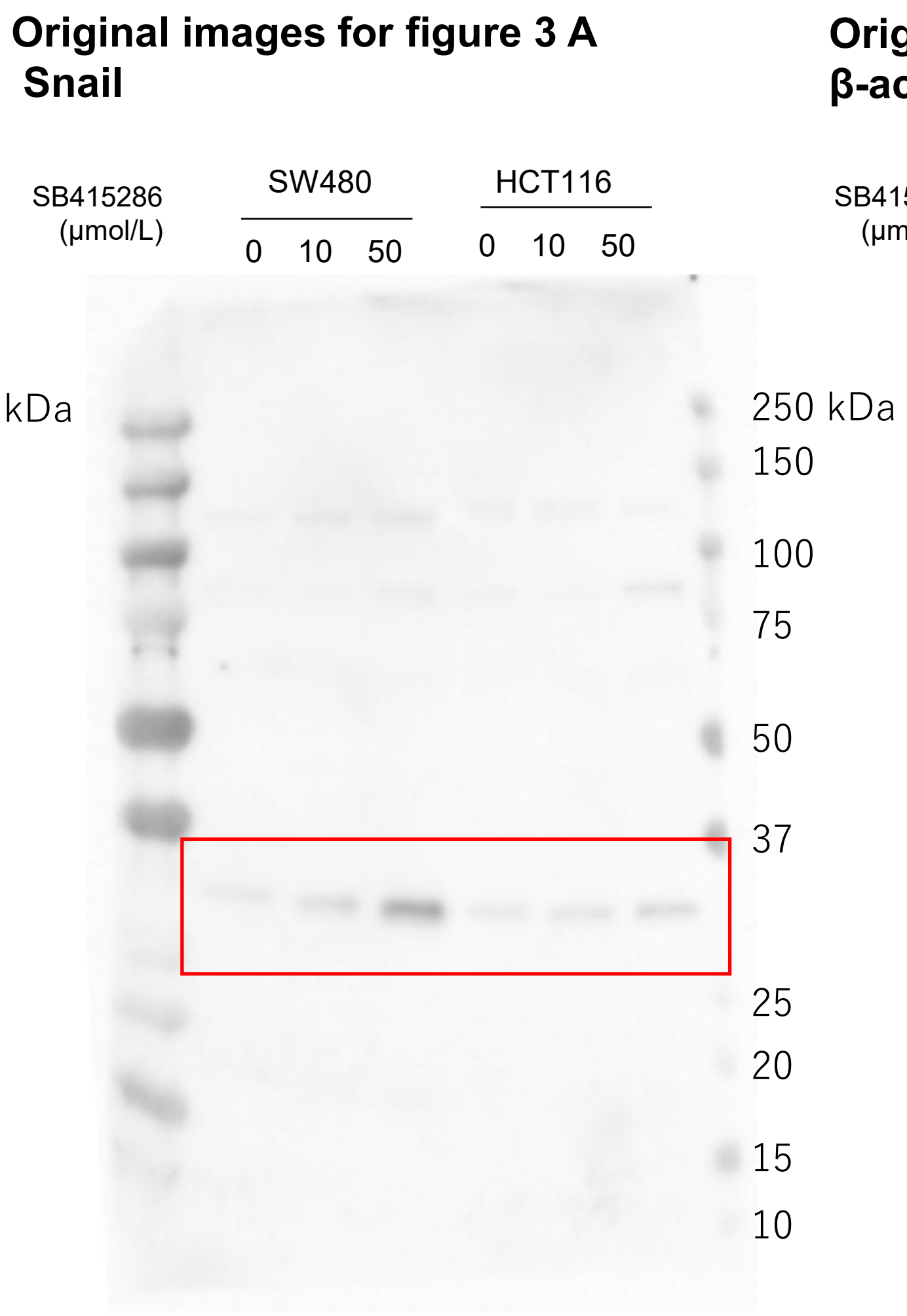

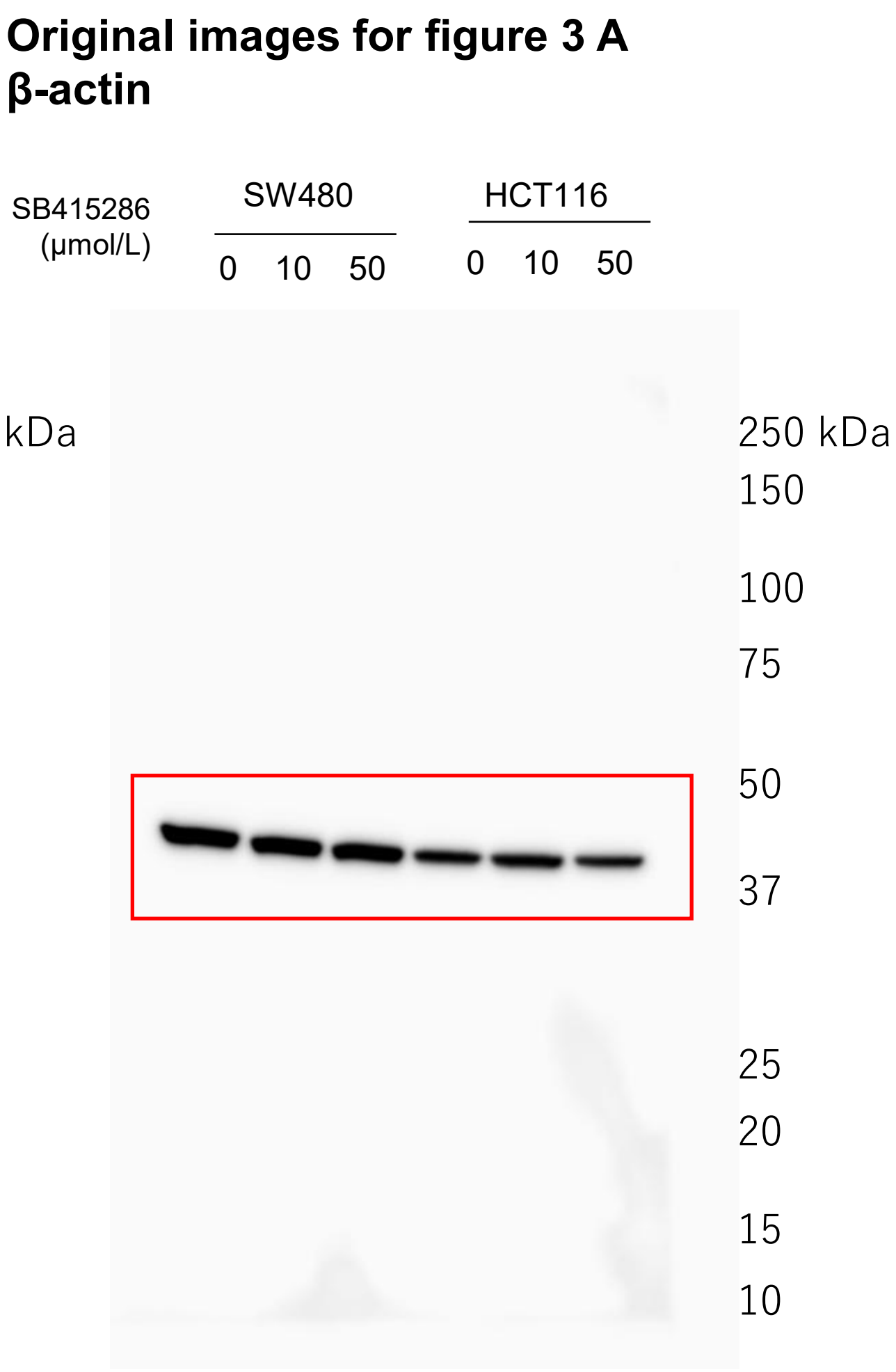

Supplementary Figure S11 Continued

Original images for figure 3 F
SW480 E-Cadherin

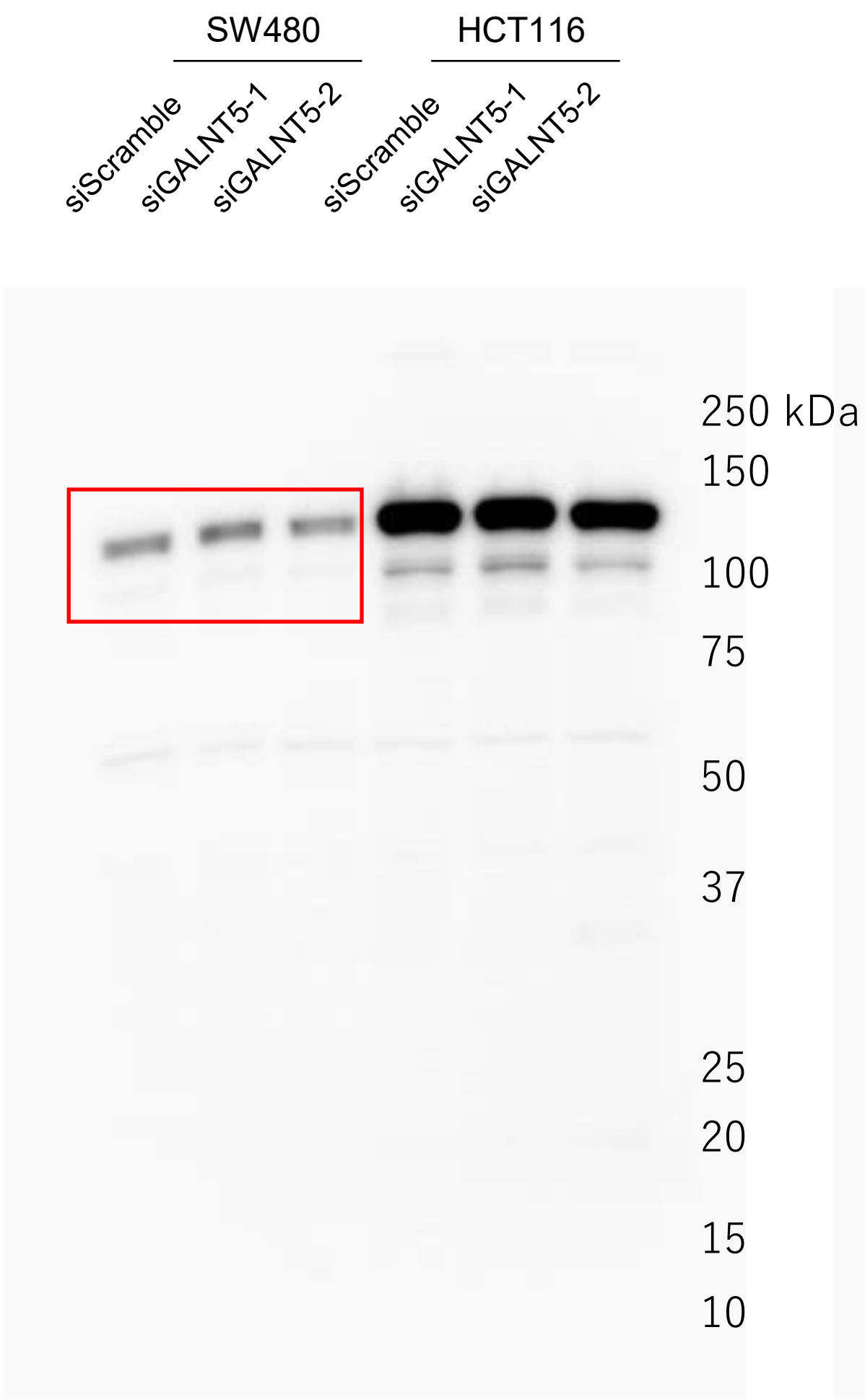

Original images for figure 3 F
HCT116 E-Cadherin

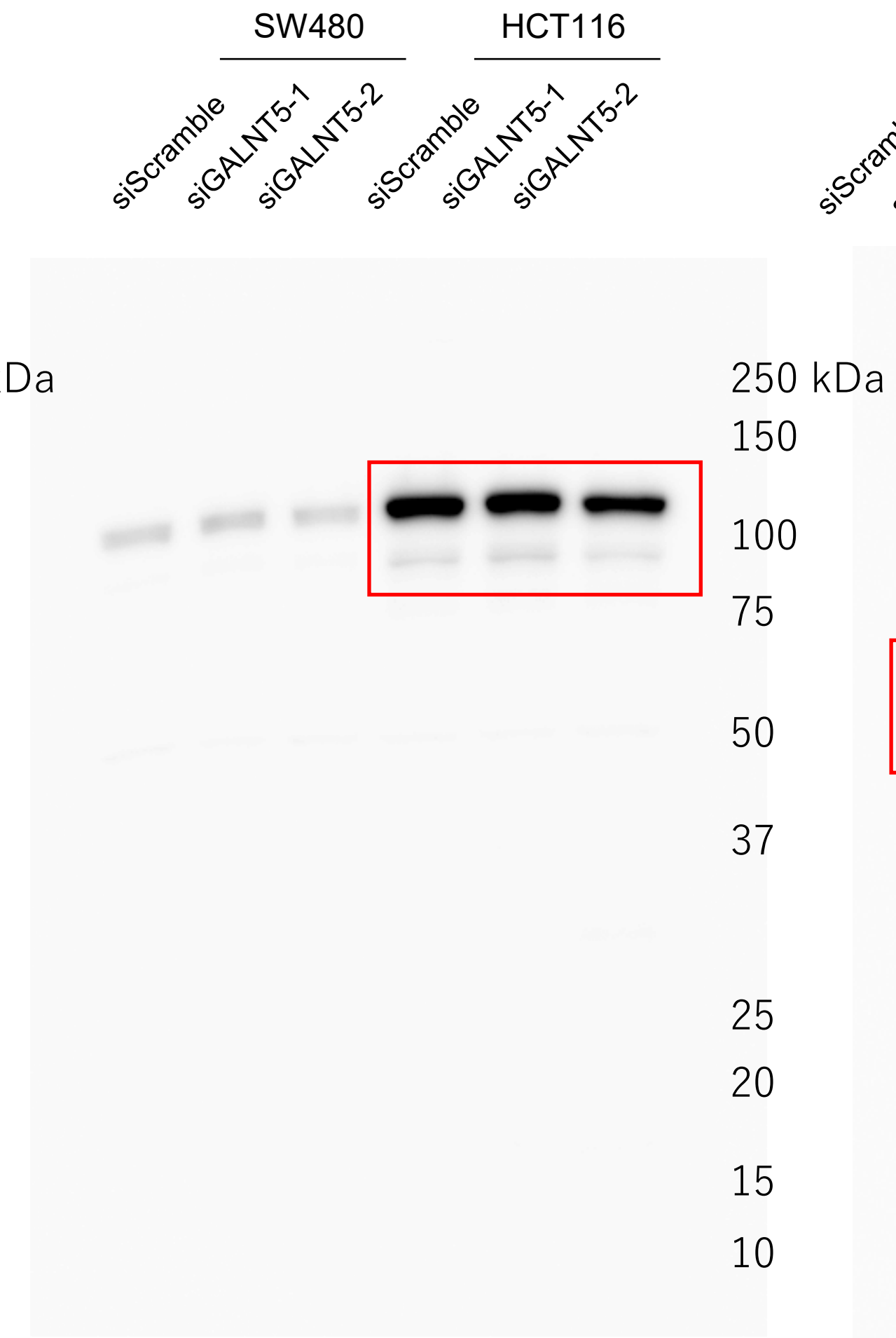

Original images for figure 3 F
SW480 HCT116 Vimentin

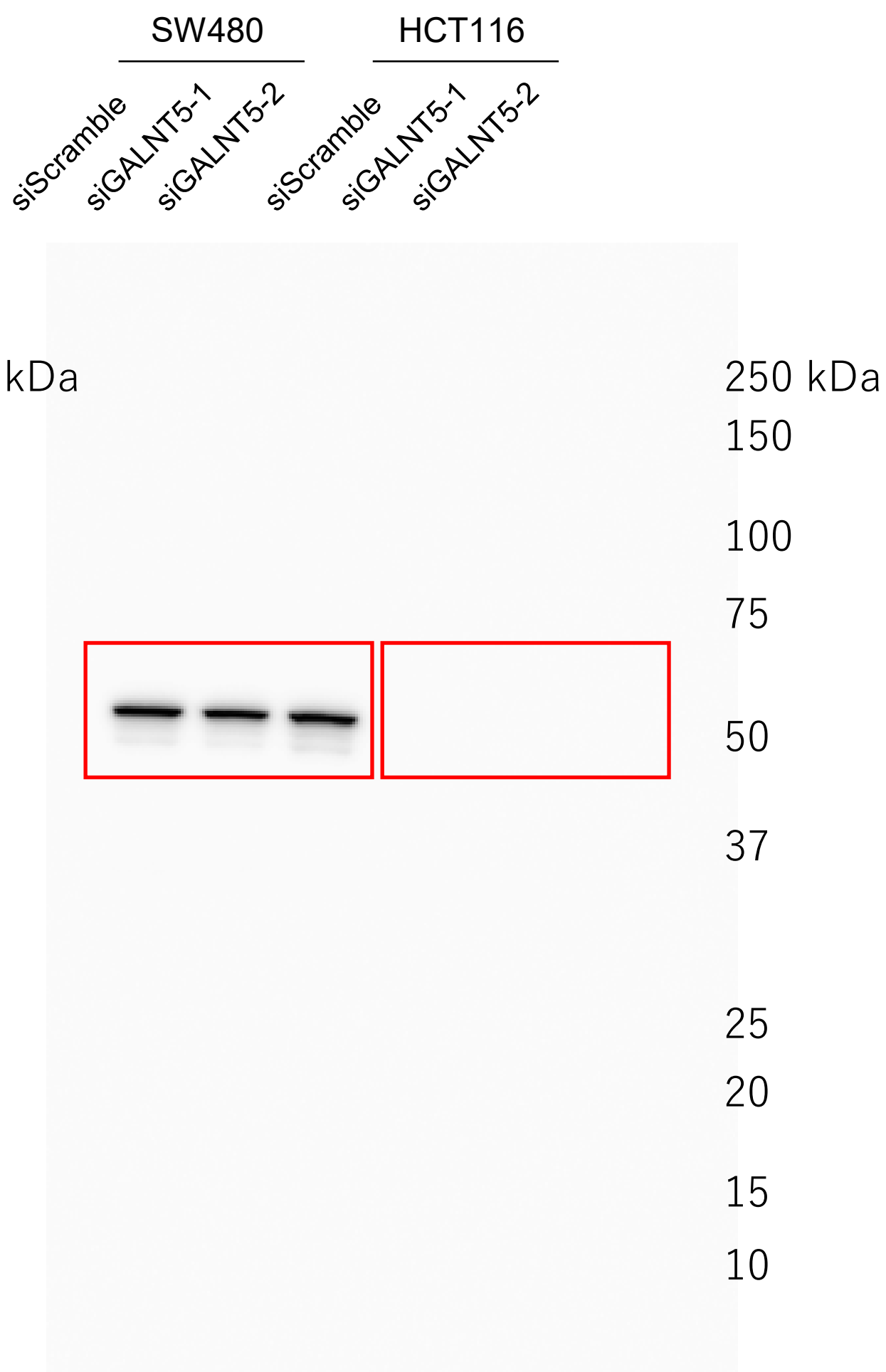

Supplementary Figure S11 Continued

Original images for figure 3 F
SW480 Snail

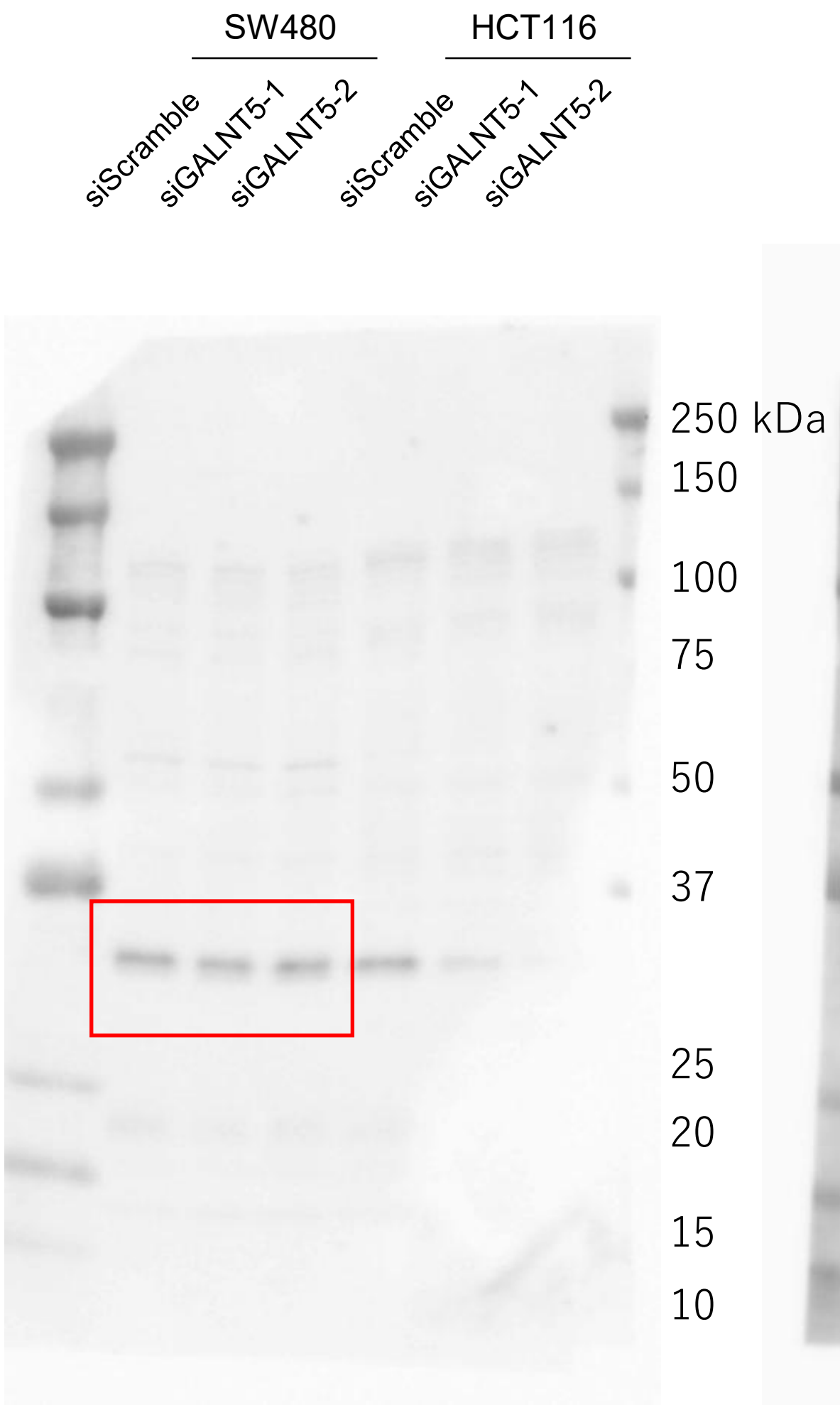

Original images for figure 3 F
HCT116 Snail

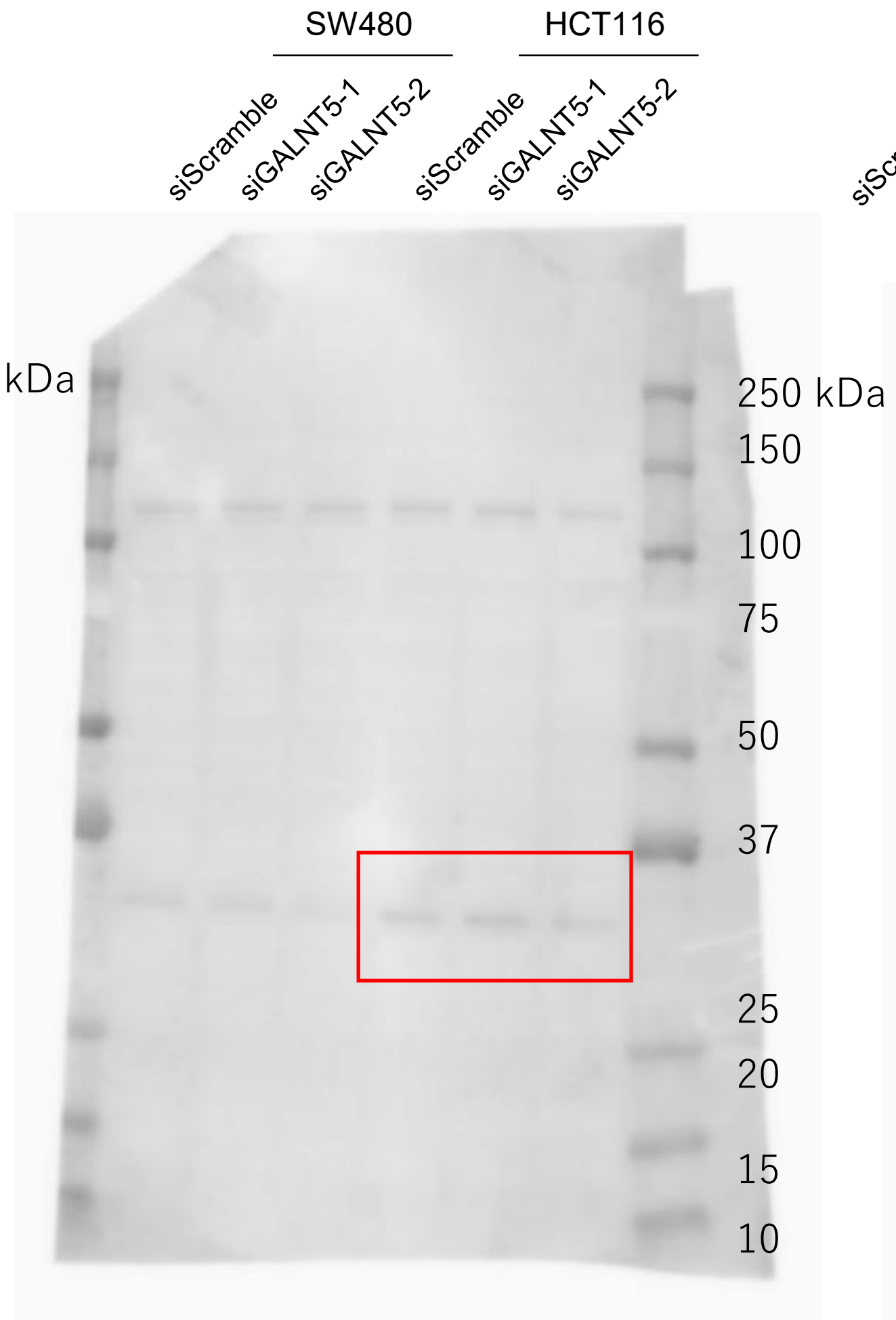

Original images for figure 3 F
SW480 HCT116 β-actin

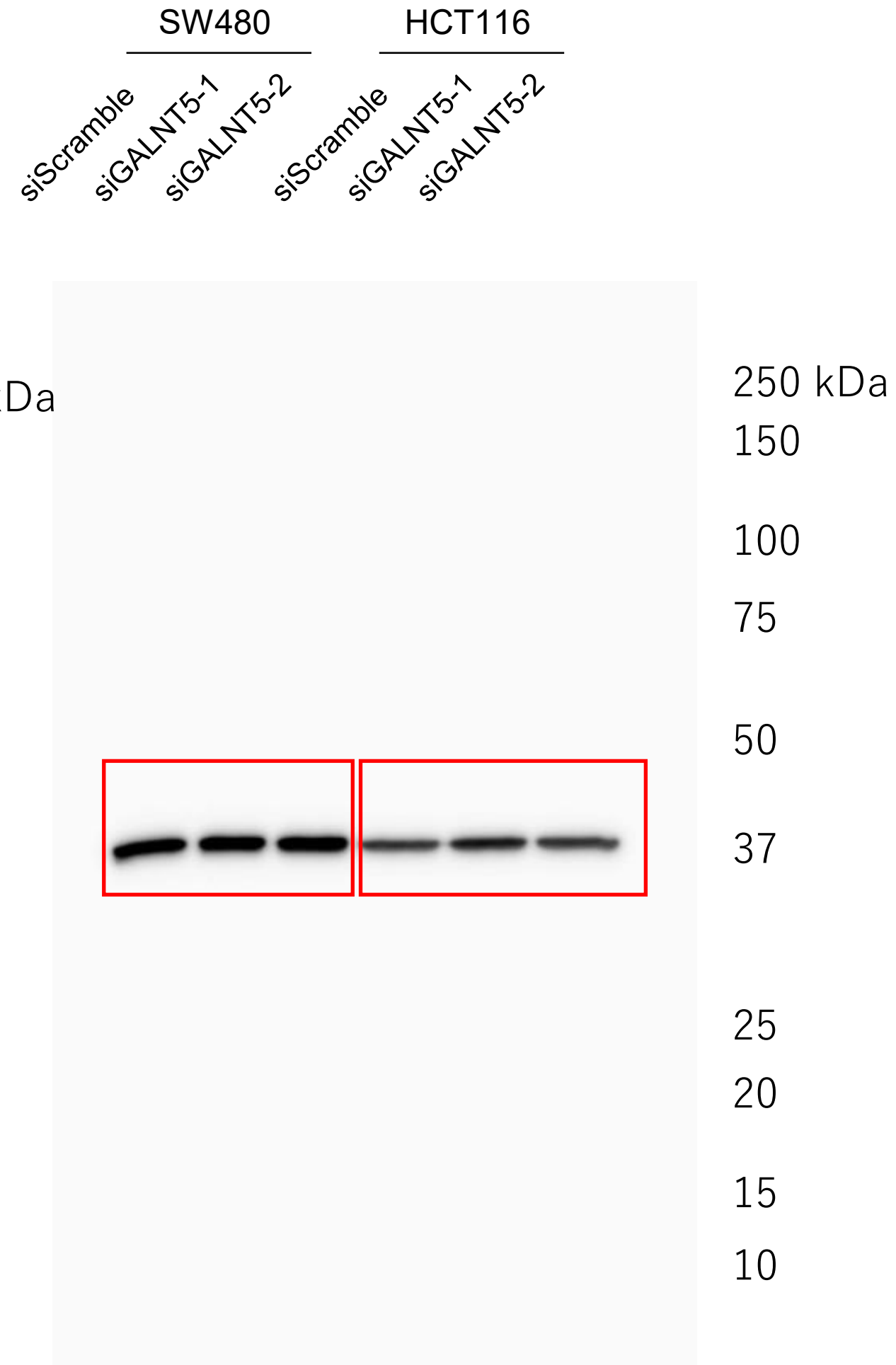

Supplementary Figure S11 Continued

Original images for figure 3 G
SW480 pAkt

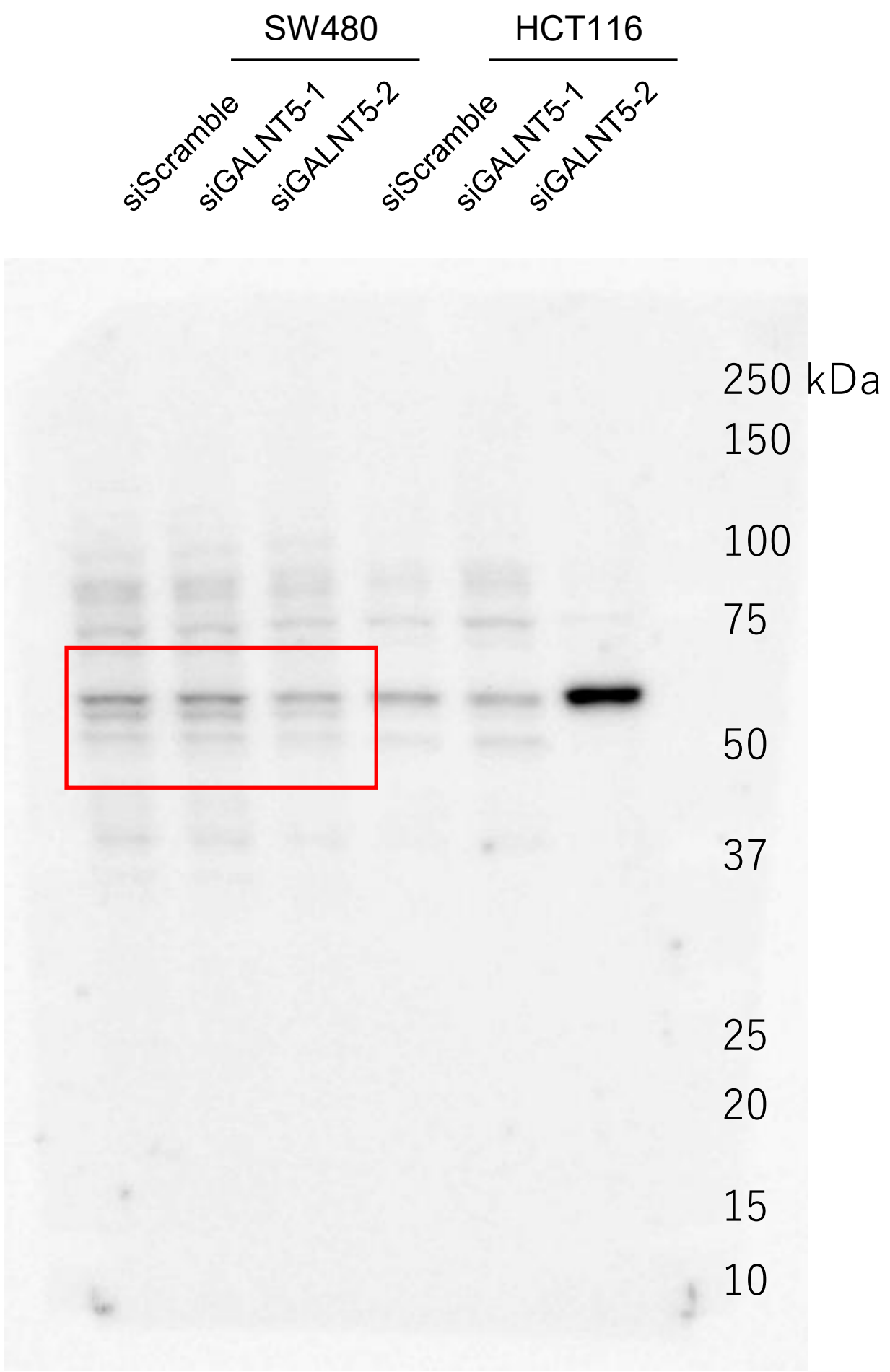

Original images for figure 3 G
HCT116 pAkt

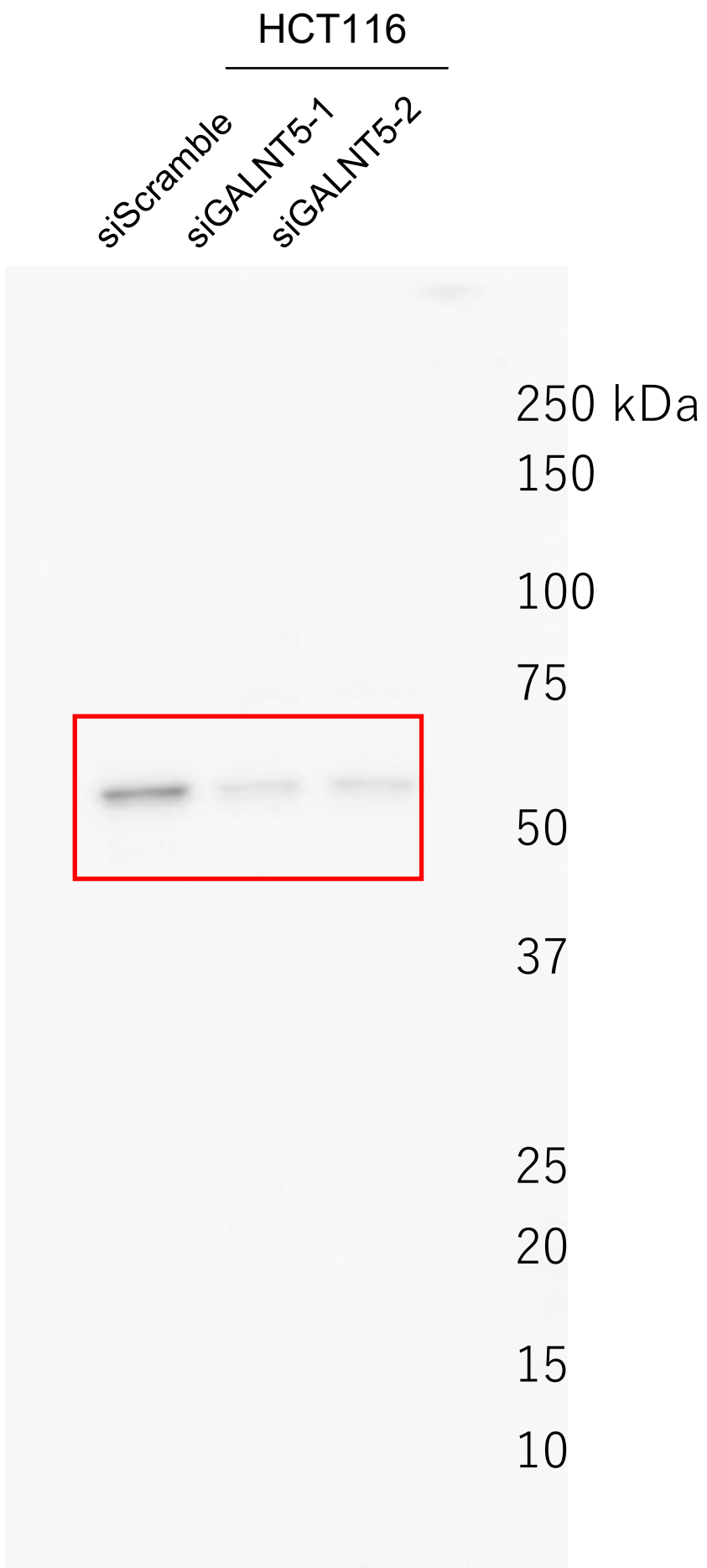

Original images for figure 3 G
SW480 Akt

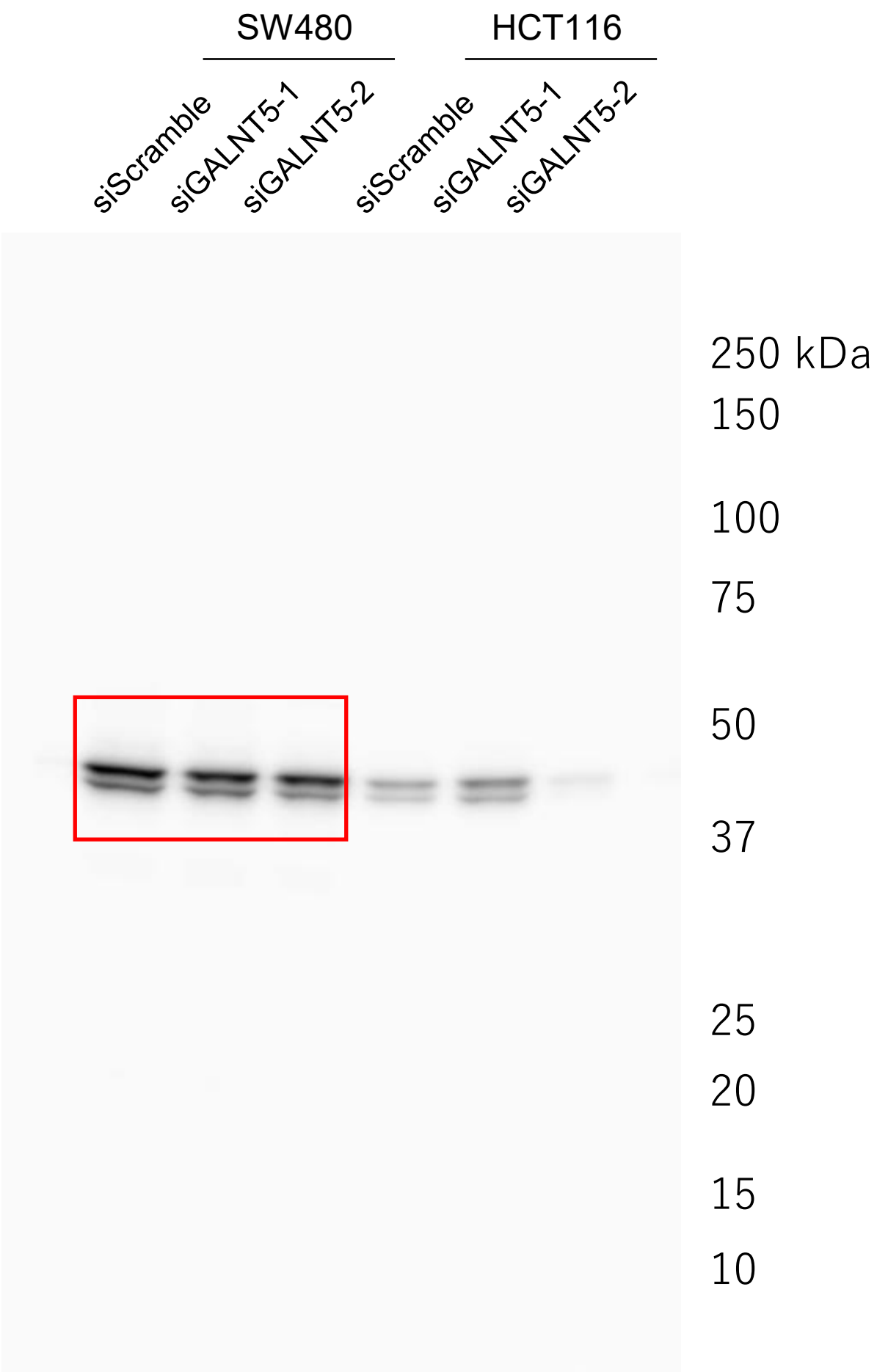

Original images for figure 3 G
HCT116 Akt

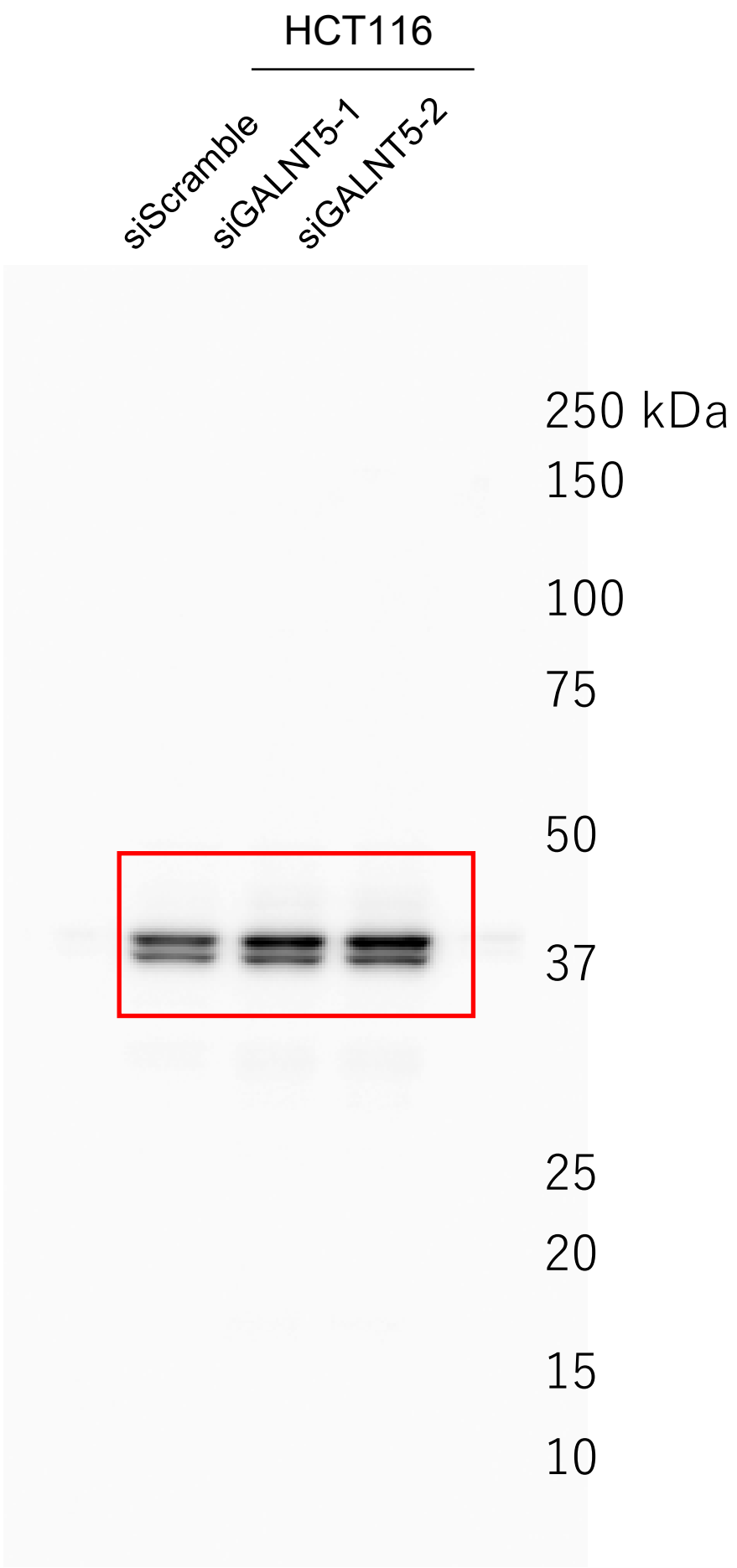

Supplementary Figure S11 Continued

**Original images for figure 3 G
SW480 pERK**

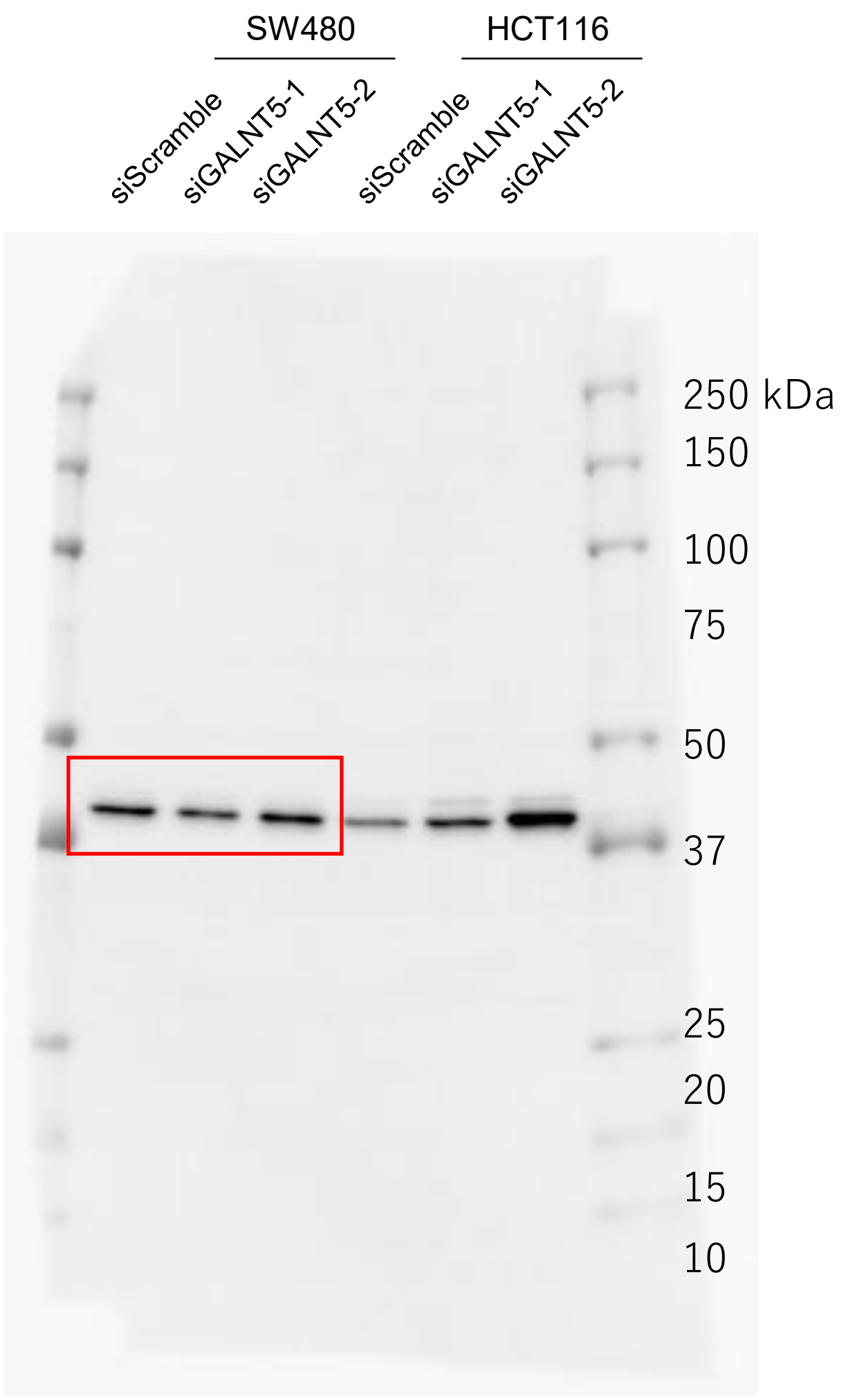

**Original images for figure 3 G
HCT116 pERK**

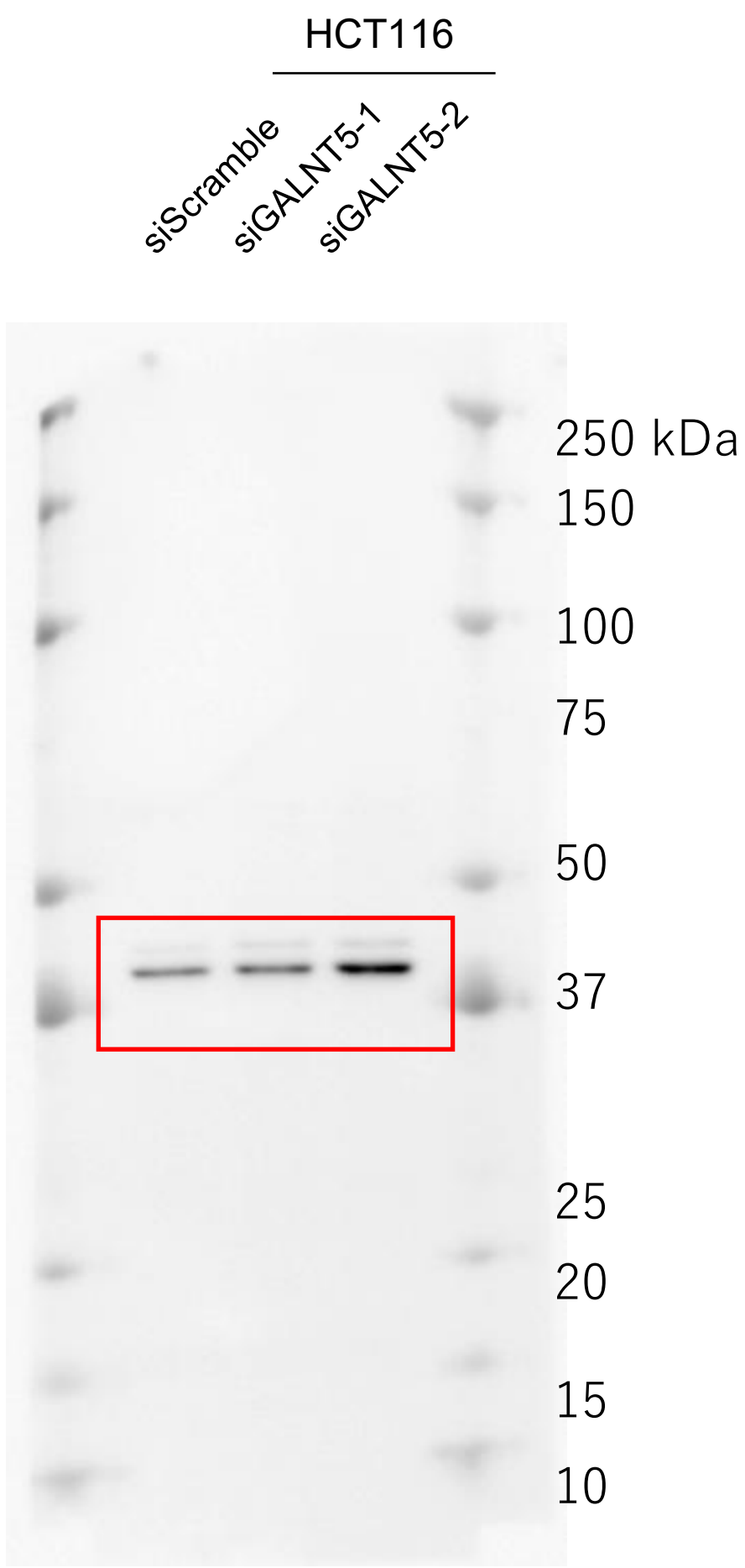

**Original images for figure 3 G
SW480 ERK**

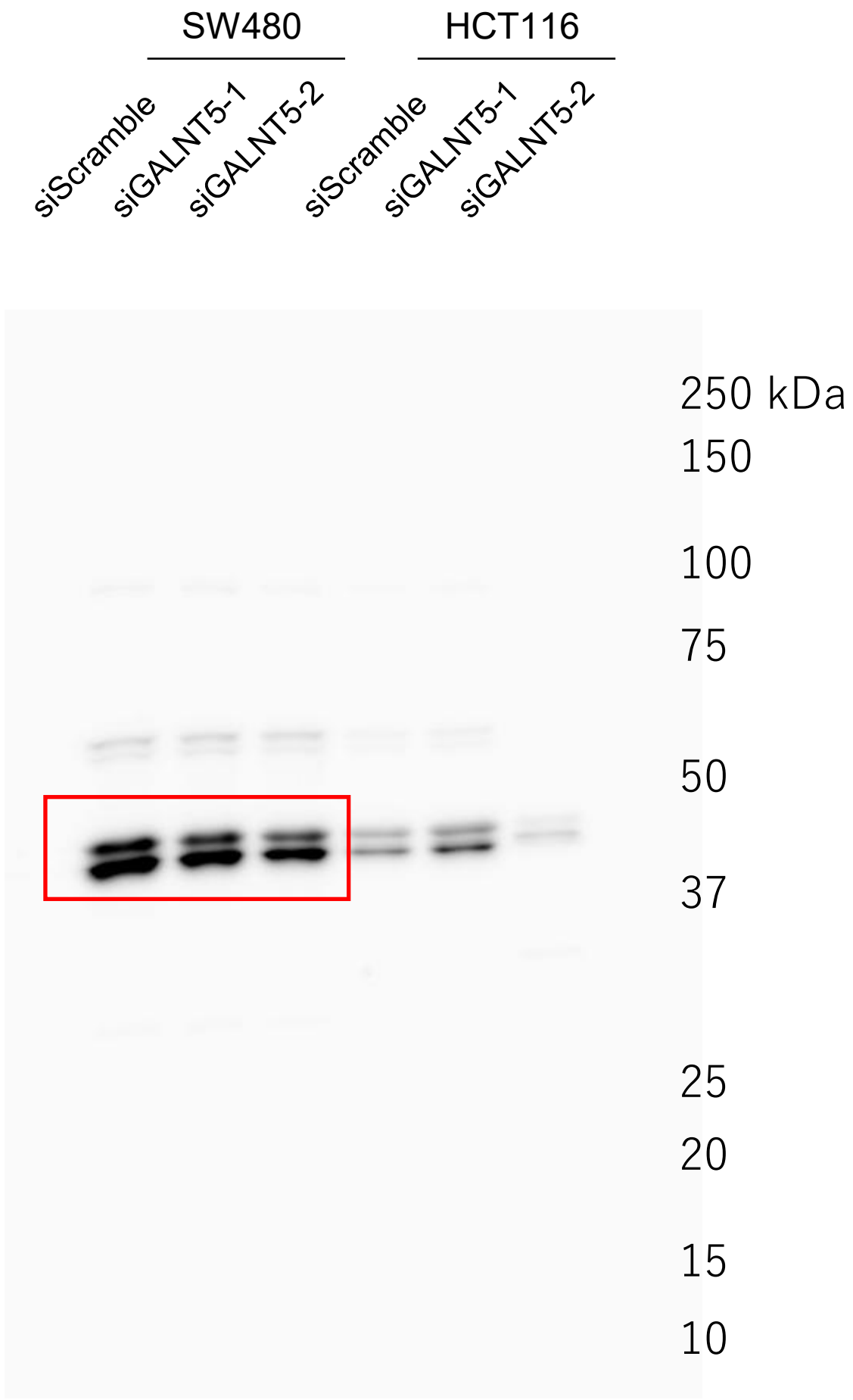

**Original images for figure 3 G
HCT116 Akt**

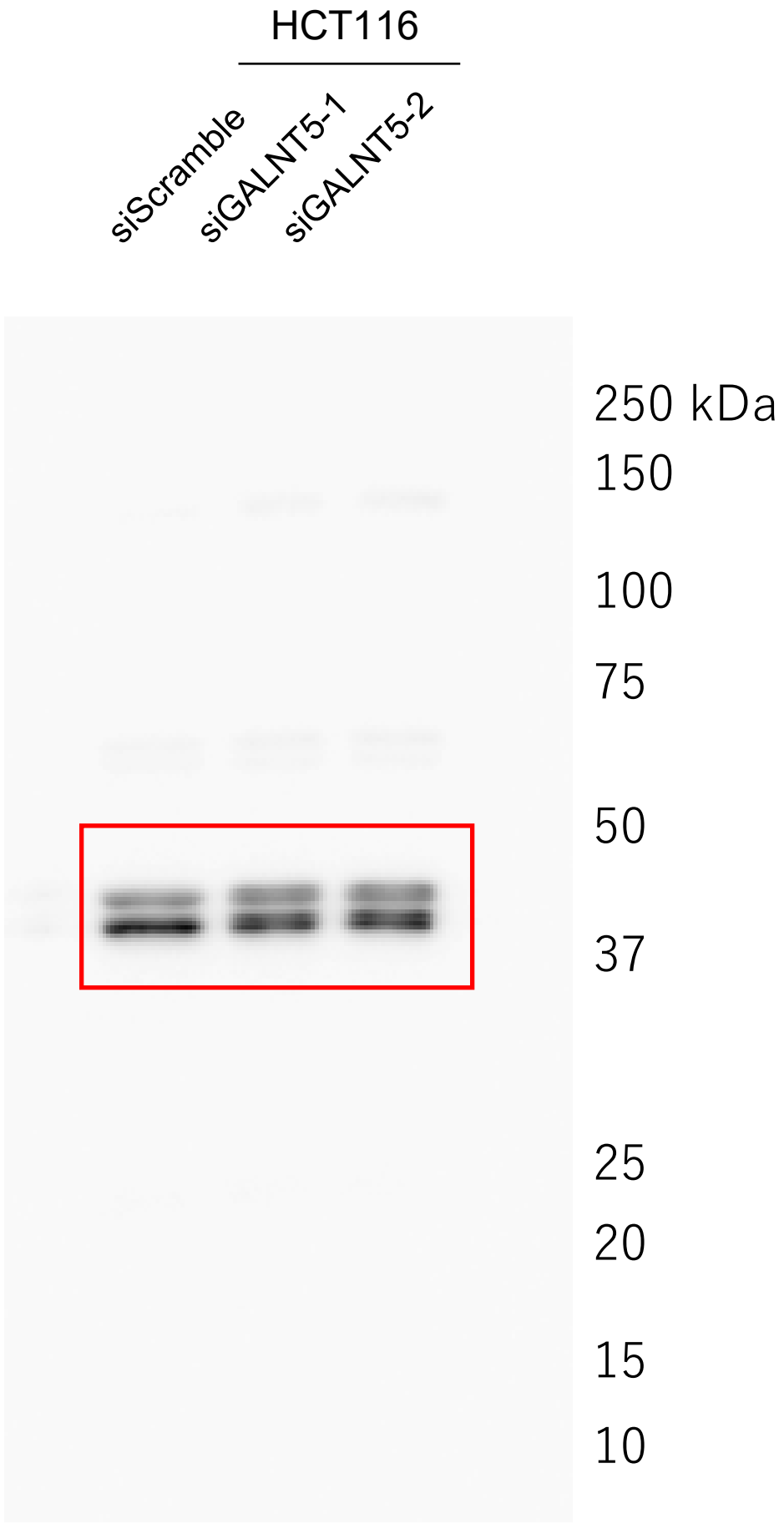

Supplementary Figure S11 Continued

Original images for figure 3 G
SW480 β-actin

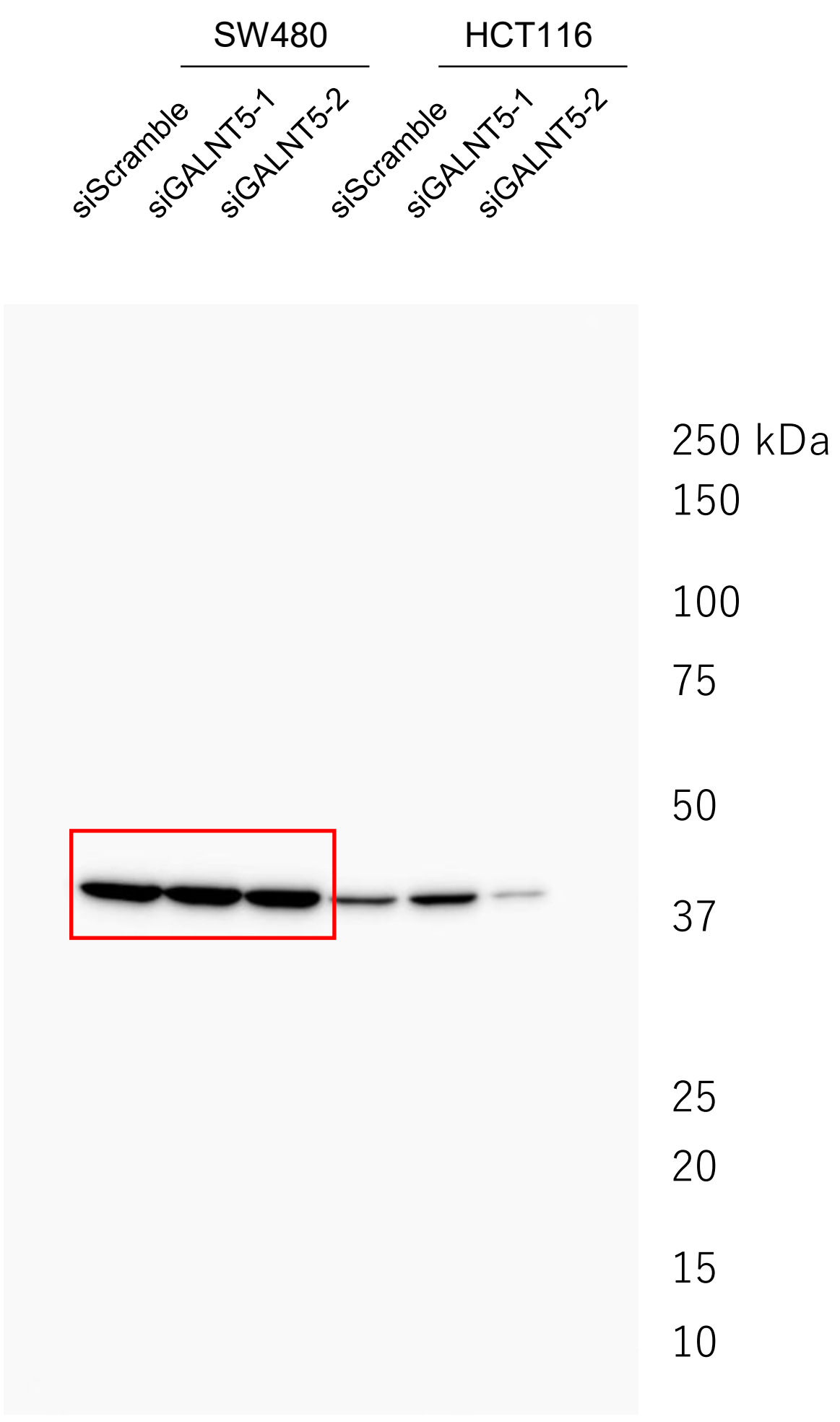

Original images for figure 3 G
HCT116 β-actin

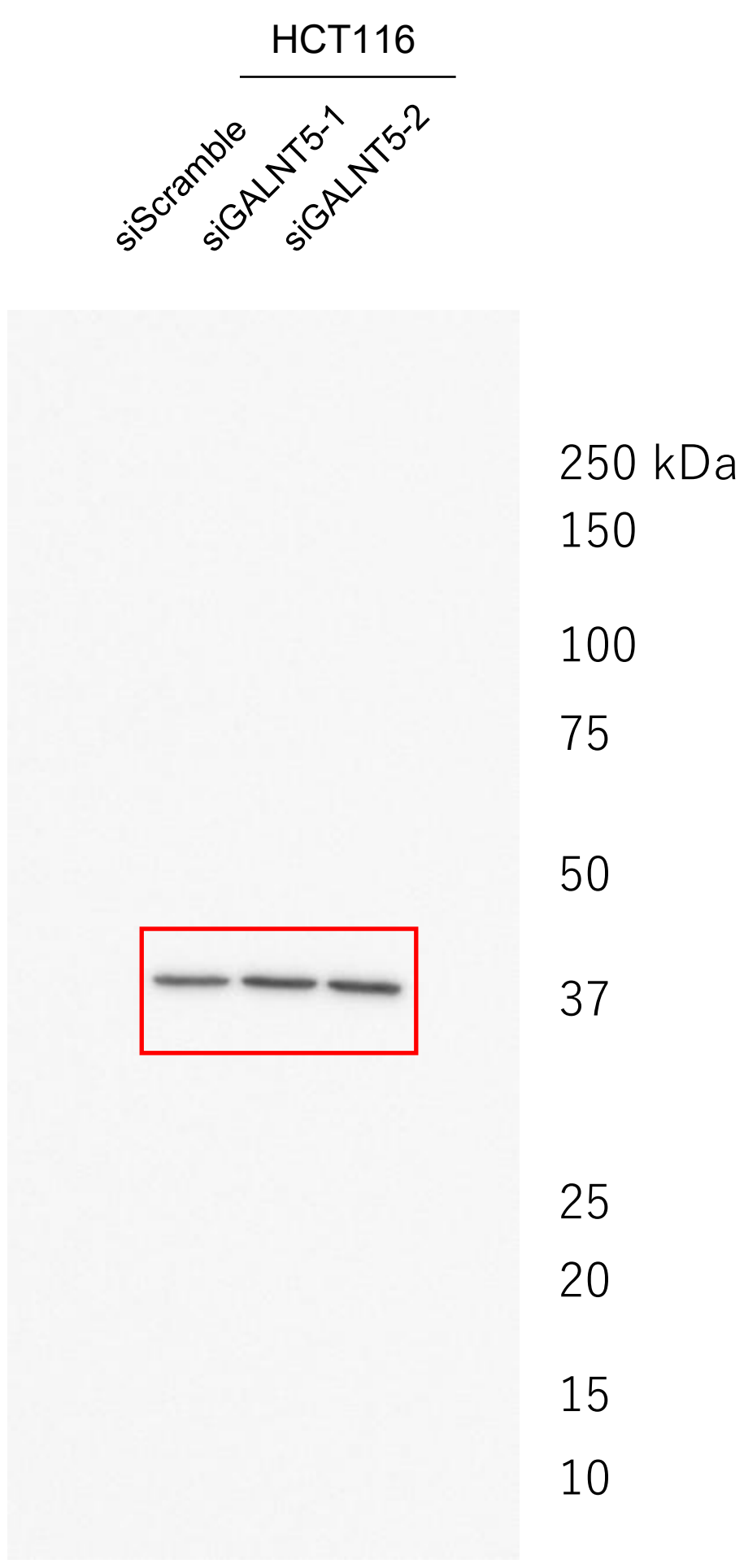

Supplementary Figure S11 Continued

Original images for figure S3 A E-Cadherin

Original images for figure S3 A Vimentin

Original images for figure S3 A Snail

Original images for figure S3 A β-actin

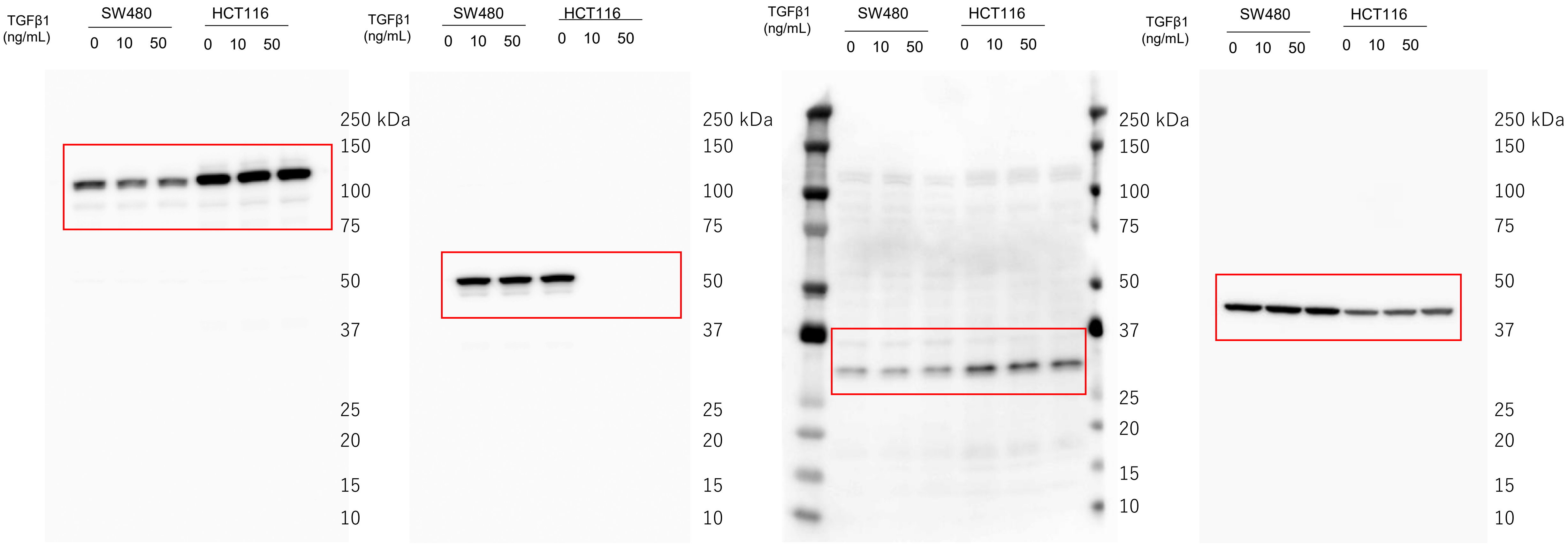

Supplementary Figure S11 Continued

**Original images for figure S5
SW480 E-Cadherin**

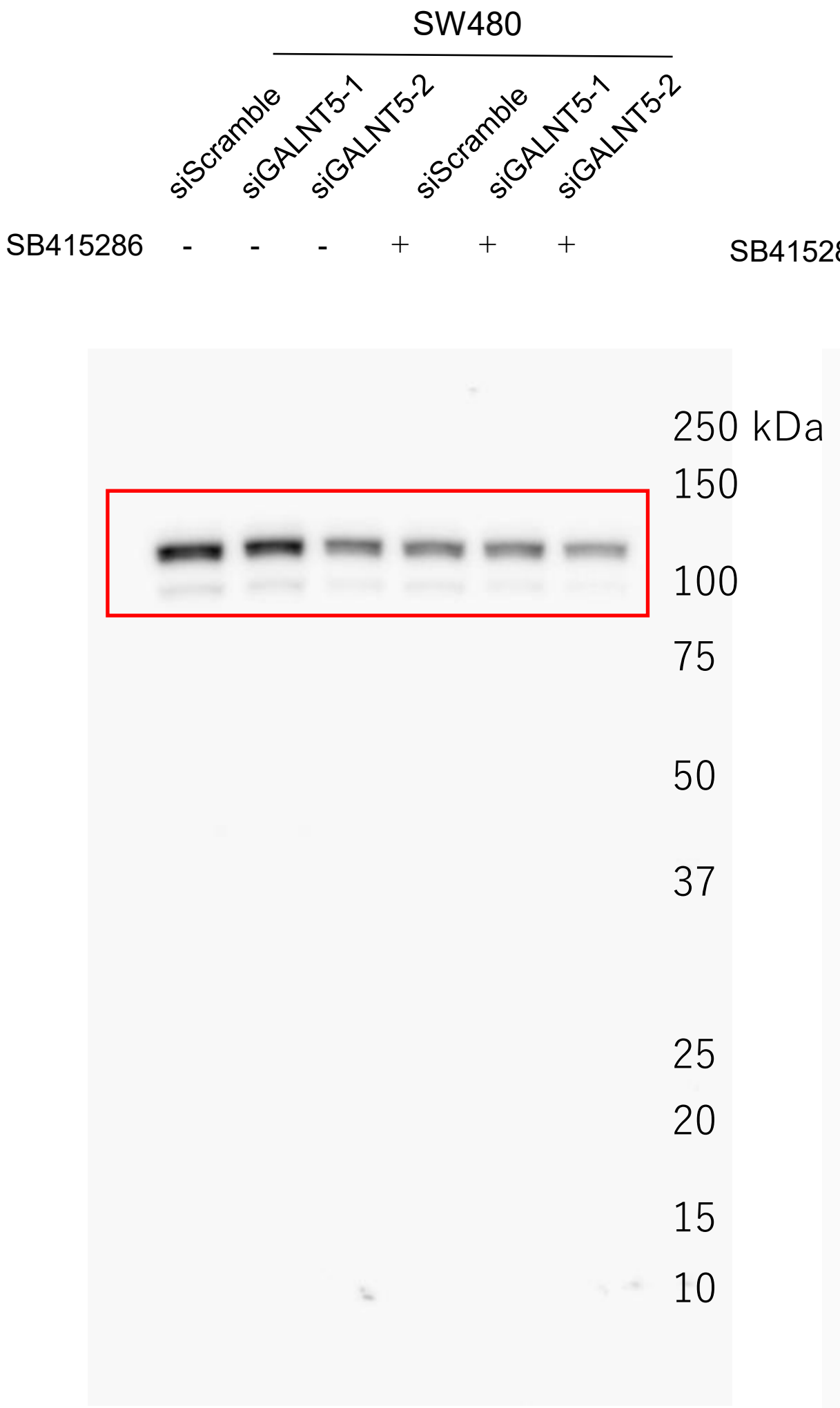

**Original images for figure S5
HCT116 E-Cadherin**

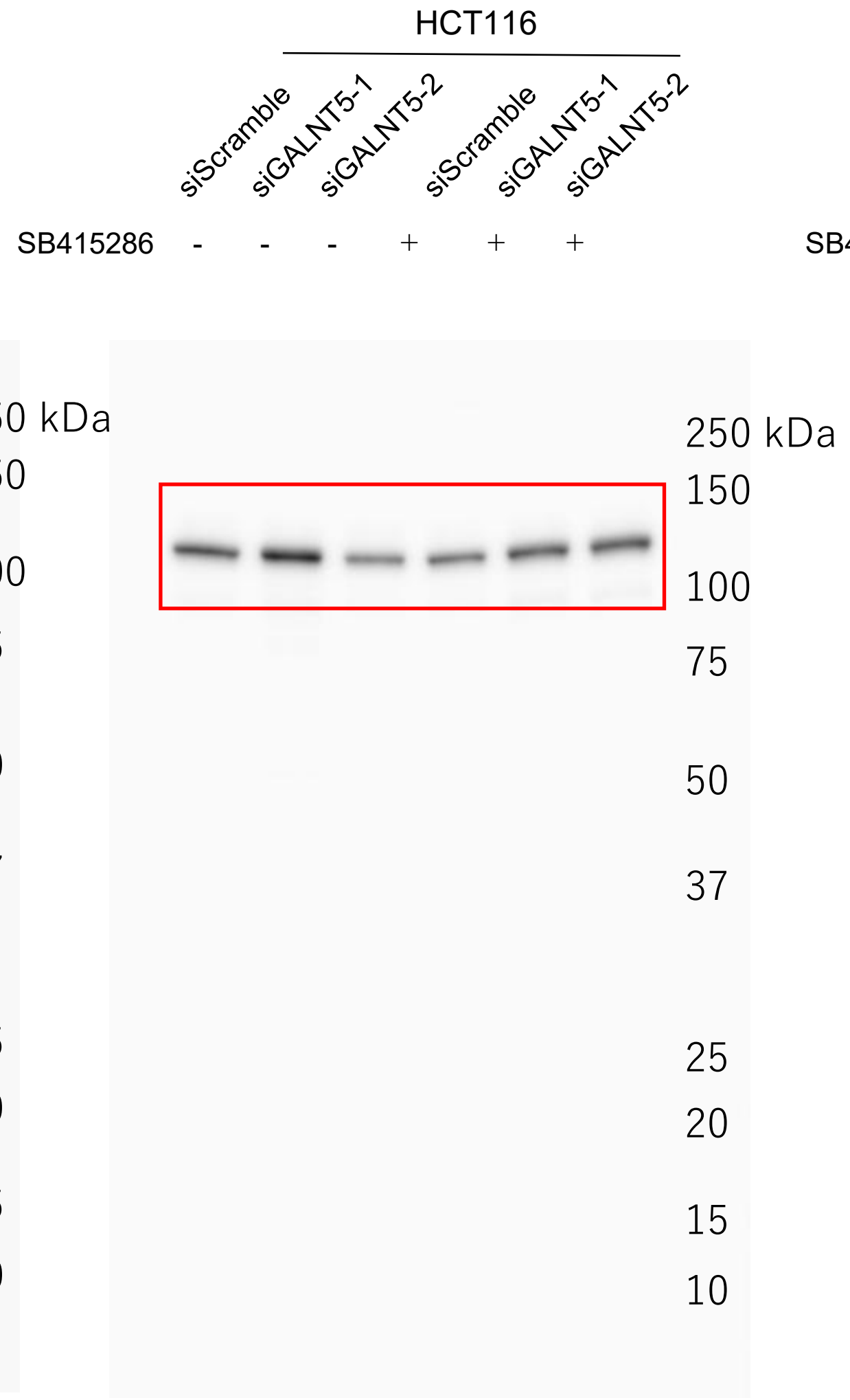

**Original images for figure S5
SW480 Vimentin**

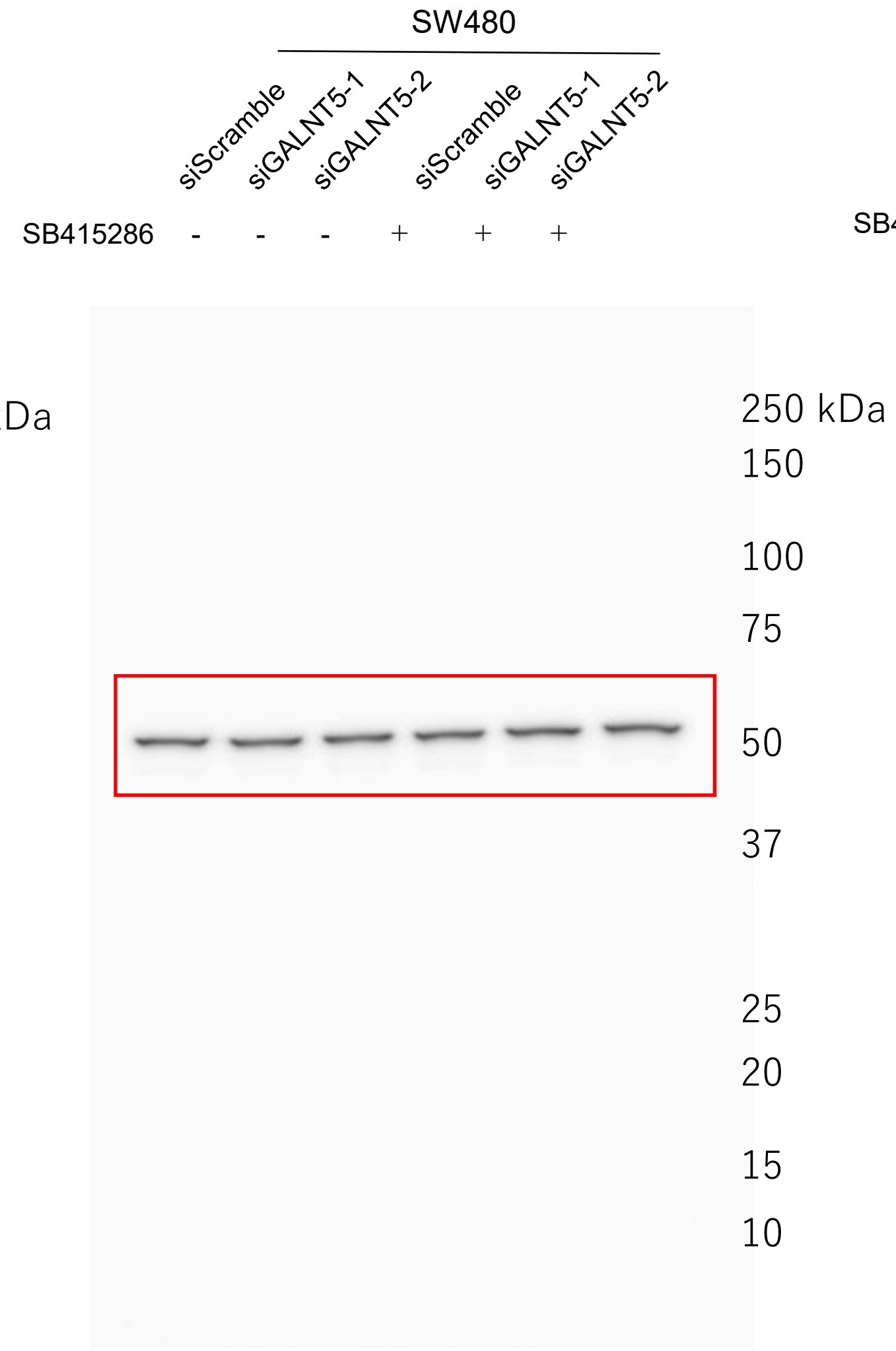

**Original images for figure S5
HCT116 Vimentin**

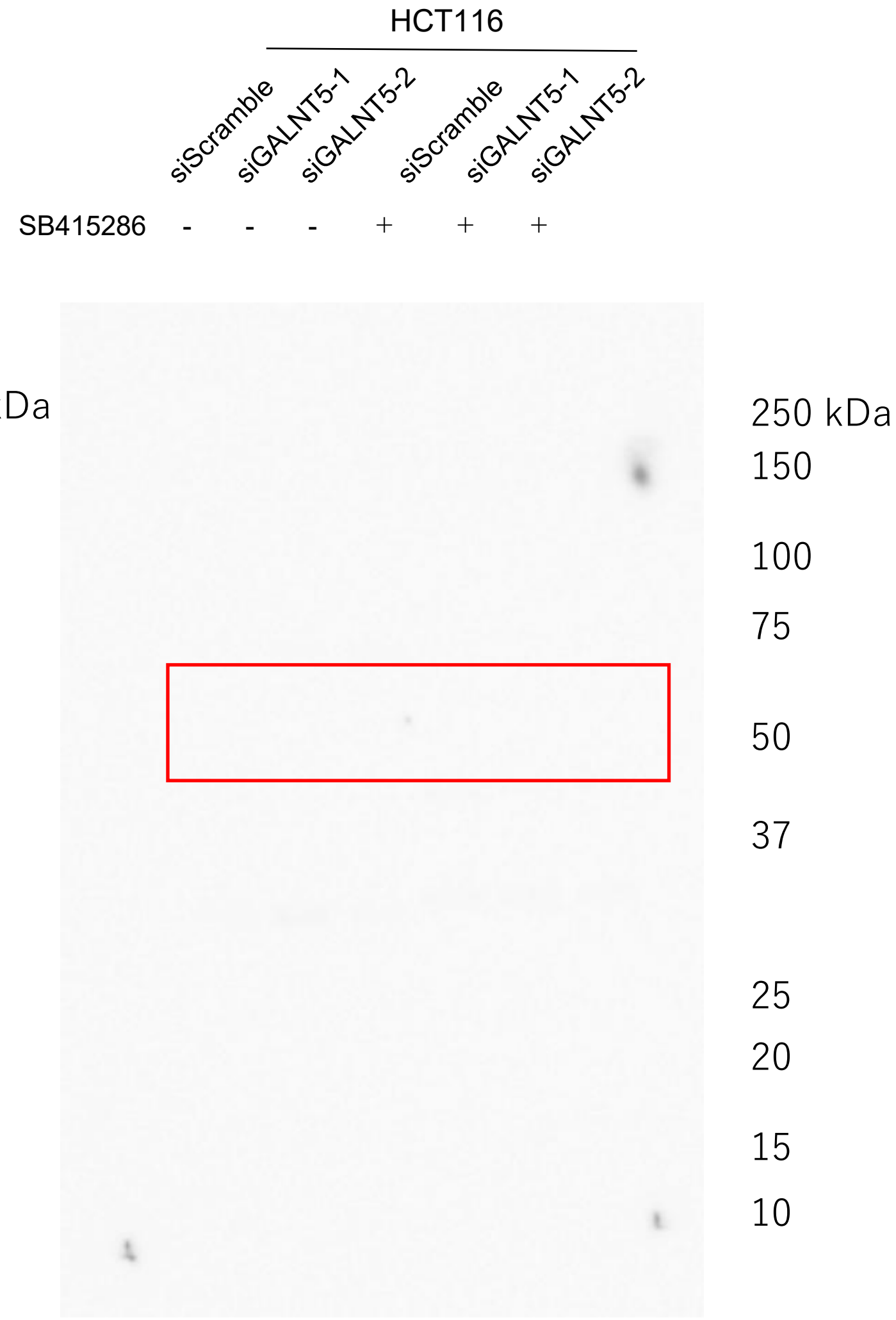

Supplementary Figure S11 Continued

Original images for figure S5
SW480Snail

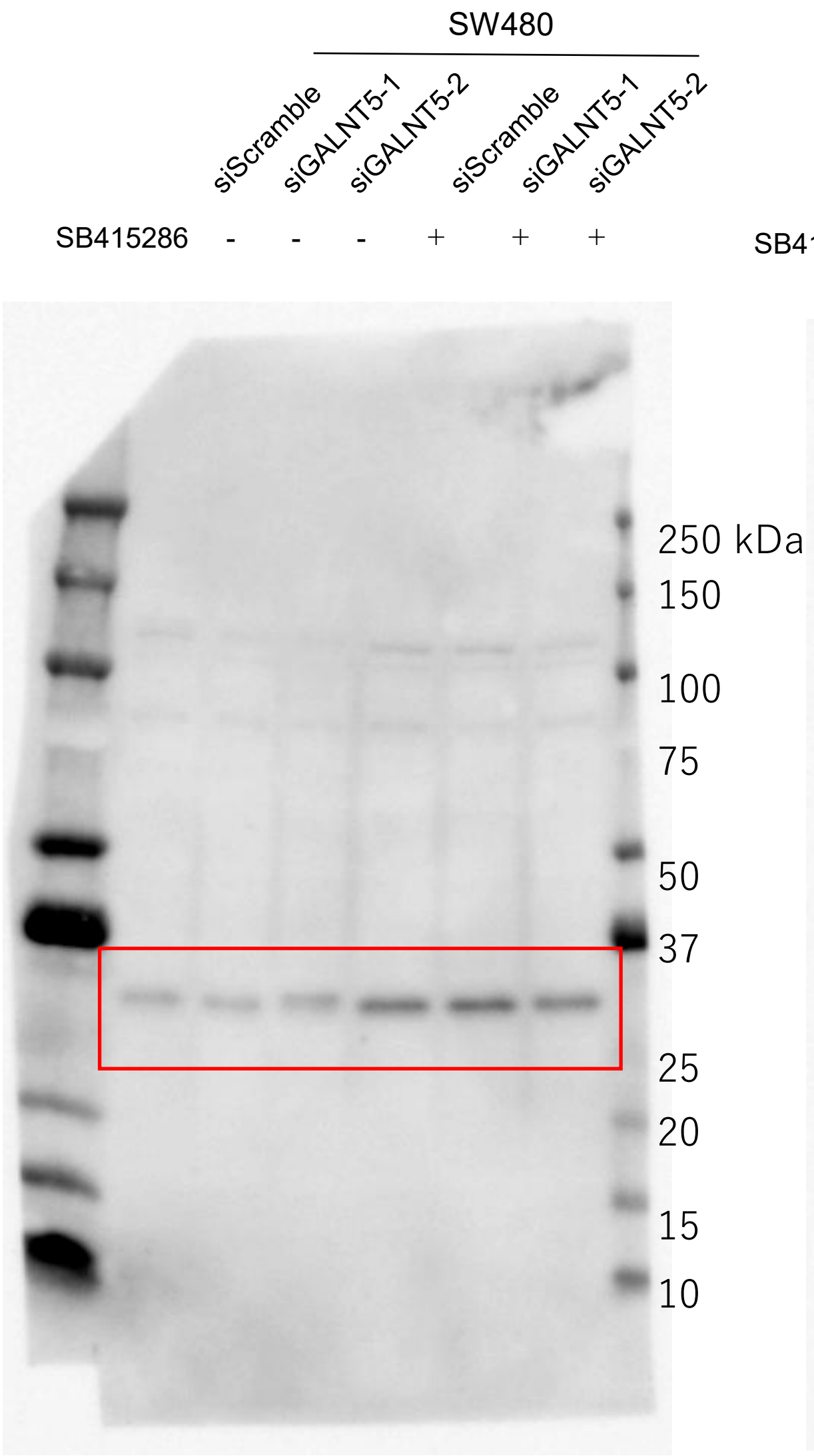

Original images for figure S5
HCT116 Snail

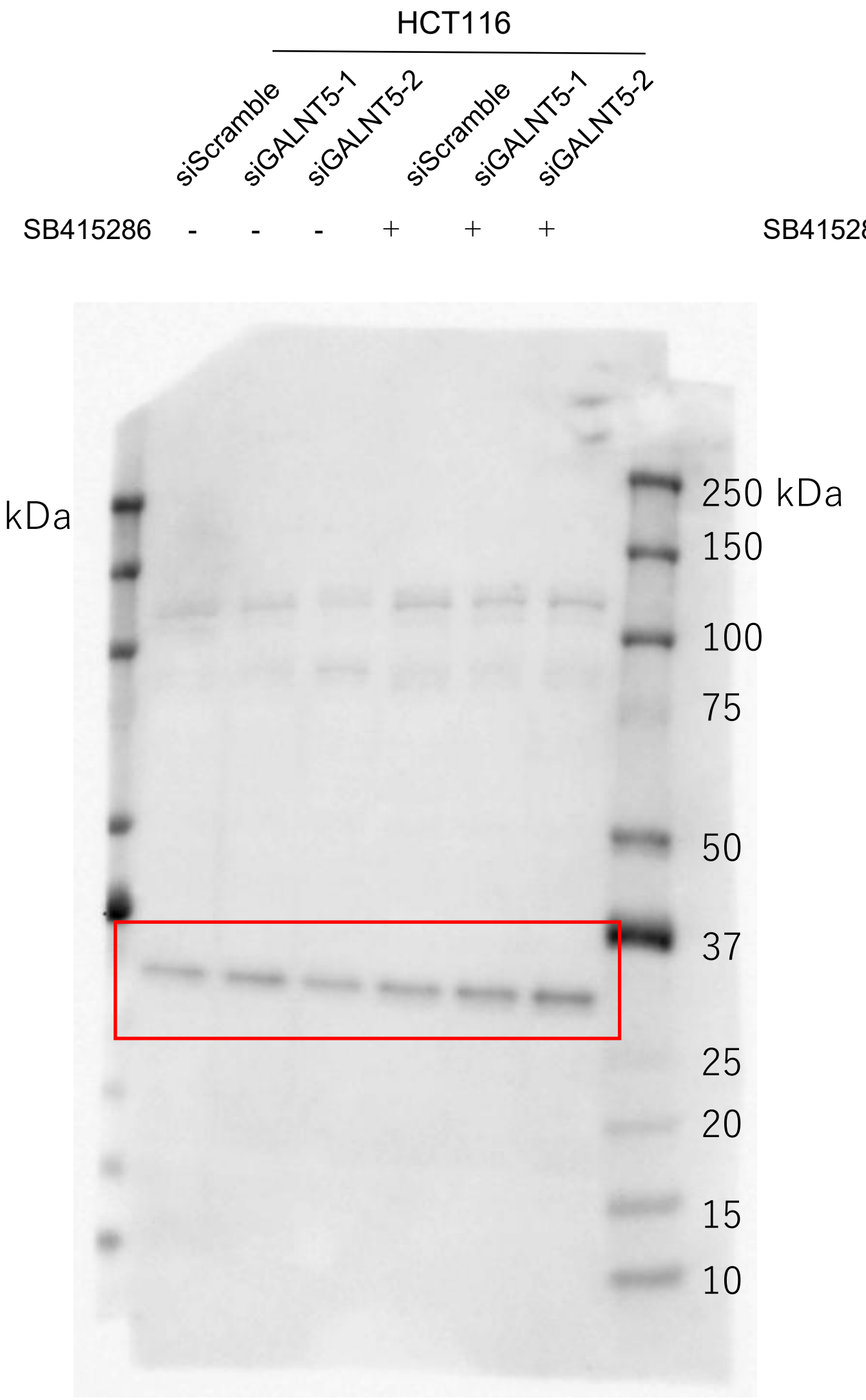

Original images for figure S5
SW480 β-actin

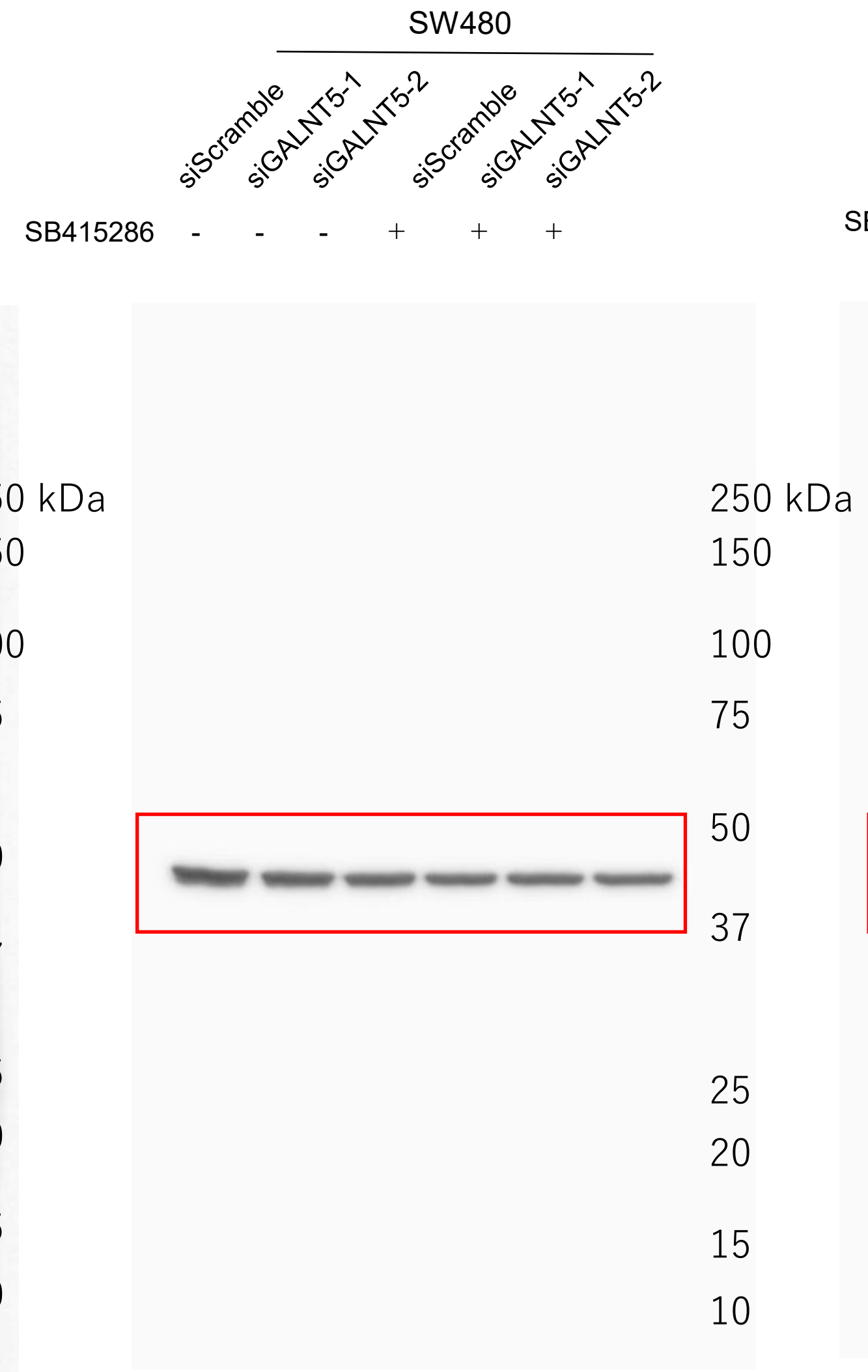

Original images for figure S5
HCT116 β-actin

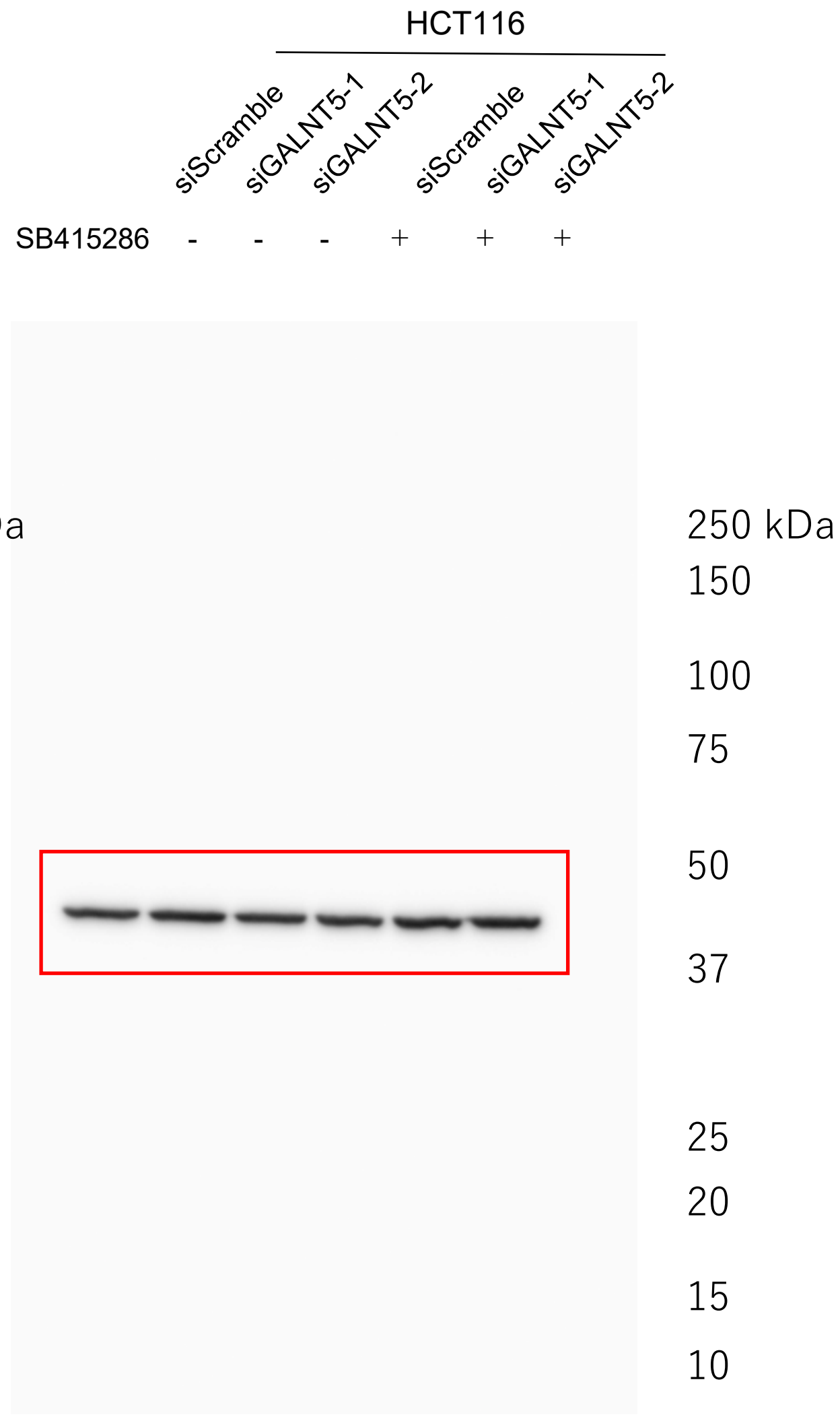

Supplement: Supplementary file 1 [file 41598_2026_57104_MOESM1_ESM.pdf]
