# Supplementary material for: CMS4 epithelial-intrinsic glycosyltransferase GALNT5 promotes tumor aggressiveness and correlates with poor survival in colorectal cancer
Source: Sci Rep. 2026 Jun 8;16:25985. doi: 10.1038/s41598-026-57104-1 (PMC13486498; doi:10.1038/s41598-026-57104-1)
Supplement: Supplementary file 2 [file 41598_2026_57104_MOESM2_ESM.pdf]

Supplementary Table S1. Two-step filtering of the 188-gene glycosyltransferase panel to identify CMS4 epithelial-enriched candidates (GSE132465)

Gene	Step-1: epithelial CMS4 enrichment			Step-2: non-epithelial depletion	
	log_2FC (CMS4 vs CMS1–3 tumor epithelial cells)	log_2FC (CMS4 tumor epithelial vs normal epithelial cells)	% expresing cells (tumor epithelium)	% expresing cells (stroma)	% expresing cells (immune)
Final CMS4 epithelial/glyco signature genes (n = 9)					
GALNT5	**1.36**	**1.85**	**24.65**	**6.25**	**0.23**
GCNT3	**0.97**	**0.87**	**24.65**	**1.17**	**0.52**
FUT2	**0.82**	**0.71**	**30.28**	**1.21**	**0.47**
B3GNT5	**0.74**	**0.75**	**43.85**	**10.96**	**6.75**
FUT6	**0.71**	**0.42**	**18.05**	**0.33**	**0.17**
MGAT5	**0.65**	**2.74**	**29.65**	**13.78**	**4.72**
FUT3	**0.58**	**0.57**	**45.22**	**2.45**	**1.01**
GALNT3	**0.55**	**1.27**	**52.89**	**1.90**	**2.26**
C1GALT1	**0.51**	**2.03**	**38.90**	**11.73**	**8.82**
Step-1 candidates excluded from the signature at Step-2 (n = 10)					
B4GALT4	**0.71**	**0.64**	**27.47**	16.74	**3.86**
CHPF2	**0.69**	**1.14**	**18.83**	26.46	**4.67**
GBA1	**0.82**	**0.40**	**15.29**	20.87	**5.92**
FUT8	**0.58**	**2.23**	**22.32**	20.43	**6.12**
CHPF	**0.41**	**2.76**	**50.38**	44.19	**6.56**
GNS	**0.46**	**1.76**	**26.72**	32.16	**11.17**
HGSNAT	**1.06**	**1.63**	**21.29**	29.71	**11.40**
MGAT2	**0.40**	**0.61**	**24.36**	26.97	**13.70**
B4GALT1	**0.88**	**0.95**	**39.39**	45.18	22.89
IDS	**0.58**	**1.83**	**37.19**	38.49	40.01
Glycosyltransferase genes excluded from the signature at Step-1 (n = 169)					
DAD1	0.13	**0.53**	**80.2**	85.1	44.9
RPN2	-0.11	**1.04**	**77.6**	63.1	27.1
DDOST	-0.09	**0.65**	**70.8**	51.7	21.2
RPN1	0.38	**0.95**	**67.2**	55.5	20.3
HEXB	-0.49	**0.62**	**66.9**	54.2	21.6
DPM1	-0.36	**1.09**	**65.9**	47.1	17.5
ALG5	-0.33	**0.94**	**61.2**	44.0	17.2
DPM3	**0.43**	0.33	**58.3**	43.9	**13.0**
STT3B	0.09	**1.08**	**57.9**	35.4	16.5
MGAT4B	-0.01	-0.01	**56.7**	20.8	**3.1**
DPM2	-0.04	**1.24**	**53.3**	40.1	**9.4**
NEU1	-0.12	**0.66**	**52.1**	46.7	21.9
B4GALT5	0.14	**1.48**	**52.0**	24.6	**8.7**
ALG3	-0.13	**1.21**	**51.4**	33.0	**8.8**
GANAB	-0.18	**0.73**	**49.9**	46.7	**9.8**
GALNT1	-0.10	**0.87**	**49.6**	33.4	**9.0**
FUCA2	-0.48	**0.49**	**47.8**	30.7	**9.2**
ALG13	0.14	**0.71**	**44.8**	29.3	21.1
GUSB	-0.07	0.22	**43.6**	30.2	**12.2**
B3GNT3	-0.05	**0.74**	**42.8**	**1.2**	**0.3**
FUCA1	0.04	-0.65	**39.6**	**8.9**	**6.2**
MGAT1	0.19	0.40	**39.0**	58.2	24.4
B4GALT3	0.12	**1.14**	**38.5**	26.6	**9.2**
HEXA	-0.37	-0.04	**37.9**	44.3	**14.2**
HYAL2	-0.21	**1.56**	**37.5**	42.8	**1.9**
GLCE	-0.59	**0.43**	**37.3**	**11.4**	**1.9**
GLB1	-0.24	**0.65**	**37.2**	22.7	**7.1**
ST6GALNAC1	**0.98**	0.14	**36.1**	**3.5**	**0.5**
MAN1A2	0.06	**0.81**	**35.5**	31.0	**8.1**
B3GAT3	-0.38	0.20	**35.5**	38.1	**12.8**
GALNT6	0.11	**1.86**	**34.5**	**3.1**	**3.1**
STT3A	0.03	**0.94**	**34.0**	24.5	**6.1**
GALNT7	0.35	**0.79**	**33.5**	**8.0**	**2.1**
C1GALT1C1	0.08	**1.14**	**32.5**	22.6	**5.6**
ALG14	0.04	0.31	**30.5**	17.4	**3.3**
MAN1B1	-0.06	**0.72**	**30.2**	32.9	**6.1**
MGAT4A	0.23	-0.11	**30.0**	**14.3**	23.1
GALNT2	0.29	**1.49**	**28.6**	30.2	**9.7**
GLA	0.03	0.30	**28.5**	24.5	**13.3**

FUT4	-0.48	0.11	**27.8**	**3.1**	**1.2**
B3GNT2	0.26	**0.81**	**27.3**	28.5	**10.6**
B4GALT7	0.11	**0.73**	**26.4**	28.7	**6.3**
ALG2	0.16	**1.60**	**25.9**	22.0	**6.9**
MAN2A1	-0.38	0.40	**25.8**	20.2	**6.6**
DPAGT1	-0.53	**0.49**	**24.7**	15.5	**4.0**
GALNT12	0.17	-0.03	**24.0**	**4.1**	**0.5**
MOGS	0.11	**0.78**	**23.6**	**12.1**	**3.7**
EXT2	-0.02	**0.83**	**23.3**	28.6	**3.0**
ABO	0.04	-0.36	**23.1**	**3.2**	**0.3**
ALG1	0.03	**0.66**	**22.9**	**13.0**	**3.1**
GALNT11	-0.03	**1.43**	**22.4**	24.2	**5.8**
ENGASE	-0.77	0.16	**21.8**	**6.3**	**2.3**
HS2ST1	0.11	**1.08**	**21.2**	21.8	**3.0**
GCNT1	-0.42	0.23	**21.2**	**3.5**	**3.0**
ST3GAL4	**0.71**	-0.01	**21.0**	32.2	**2.3**
B4GALT2	0.33	**1.30**	**20.6**	18.2	**0.6**
ALG8	-0.41	0.39	**20.6**	**14.0**	**4.3**
RFT1	-0.15	**1.14**	**19.8**	**11.3**	**3.0**
ST6GAL1	0.02	0.11	**18.5**	**5.8**	**6.5**
ST6GALNAC4	0.39	**1.06**	**18.1**	18.0	**6.9**
SLC33A1	0.36	**1.32**	**18.0**	**12.0**	**3.5**
B3GALNT2	0.33	**1.56**	**18.0**	**8.2**	**2.0**
EXT1	0.31	**0.40**	**17.7**	28.4	**1.6**
B3GALT6	0.12	**1.86**	**17.1**	15.9	**3.2**
AGA	-0.55	0.24	**16.8**	**12.9**	**6.6**
GALNT10	-0.14	**0.94**	**16.7**	17.4	**4.9**
MAN2C1	0.17	-0.25	**16.0**	19.4	**4.8**
GALNS	-0.59	**0.43**	**15.5**	23.0	**2.9**
CHST12	-0.01	0.25	14.5	35.9	**13.1**
ALG12	-0.19	-0.09	14.3	**13.1**	**2.4**
HS3ST1	**1.69**	**2.63**	14.0	**2.4**	**3.6**
MAN1A1	0.17	-0.28	13.9	29.2	**8.2**
MANBA	-0.13	0.25	13.9	**12.0**	**6.9**
NAGA	0.07	-0.16	13.4	**14.0**	**7.1**
B3GNT7	**1.78**	-0.89	13.1	**2.2**	**3.5**
ALG6	0.12	**0.57**	13.0	**6.6**	**2.4**
MAN2B2	0.12	**0.46**	12.7	**12.7**	**3.4**
MAN2A2	-0.04	**0.62**	12.4	**5.0**	**2.2**
NAGLU	0.39	**0.59**	12.2	**13.3**	**2.0**
HS6ST1	0.04	0.19	10.6	**11.2**	**1.3**
ST3GAL2	-0.12	**2.72**	10.2	20.3	**3.4**
DOLPP1	0.21	-0.30	9.9	**4.8**	**1.1**
EXTL2	-0.31	**0.66**	9.9	20.6	**0.9**
B3GALT4	**0.71**	0.12	9.8	**11.4**	**3.1**
CHSY1	-0.16	**1.11**	9.6	32.7	**3.5**
HYAL3	-0.67	**0.47**	9.2	**4.0**	**0.8**
NEU4	**0.76**	-1.42	8.4	**0.5**	**1.4**
ST3GAL1	**0.76**	**4.14**	7.3	24.0	**11.3**
HS6ST2	-1.02	**3.39**	7.0	**1.4**	**0.0**
ST6GALNAC6	**1.33**	-2.25	6.8	32.3	**5.8**
NDST1	0.33	**0.61**	6.6	22.5	**1.0**
B3GAT2	0.39	-0.56	6.6	**6.3**	**3.3**
XYLT2	**0.55**	**0.80**	6.6	**9.7**	**0.9**
SGSH	-0.21	0.23	6.3	**9.3**	**1.8**
EXTL3	**0.84**	**2.15**	6.3	**9.5**	**0.8**
B4GAT1	-0.86	**0.49**	6.0	**11.1**	**0.9**
GALNT18	-1.04	**2.19**	6.0	**13.2**	**0.0**
HPSE	-0.45	-0.21	5.6	**1.7**	**5.9**
XYLT1	**0.48**	-0.18	5.3	**7.4**	**1.7**
FUT1	0.10	**4.06**	5.2	**1.8**	**0.1**
B3GALT5	**2.34**	-0.06	5.1	**1.0**	**0.1**
NEU3	-0.92	**1.28**	4.4	**1.7**	**1.1**
IDUA	**1.13**	**0.74**	4.4	**9.5**	**1.8**
ALG11	0.06	**0.87**	3.9	**2.3**	**0.8**
GALNT8	**2.83**	**2.52**	3.7	**0.6**	**0.1**
ALG9	-0.34	0.37	3.7	**3.4**	**0.6**
ALG10	-0.38	**1.25**	3.7	**1.6**	**0.7**

DSE	**0.46**	**1.72**	3.4	25.3	**11.2**
CHST14	-0.09	**1.01**	3.4	20.6	**0.8**
CHST13	-1.14	-0.66	3.3	**0.1**	**0.9**
CHST7	**0.48**	-0.44	3.1	**9.4**	**4.5**
NDST2	**0.42**	0.01	2.9	**1.7**	**1.8**
ARSB	0.32	0.03	2.8	**9.6**	**2.6**
ALG10B	**0.48**	**1.20**	2.7	**3.2**	**0.6**
HYAL1	**1.76**	**1.00**	2.6	**4.1**	**0.1**
GBGT1	-0.17	**0.79**	2.5	**6.4**	**2.3**
MAN2B1	**0.76**	**0.52**	2.5	**3.8**	**2.9**
CHST15	-0.54	NA	2.3	15.8	**3.8**
B3GALNT1	**0.82**	NA	2.1	**9.2**	**0.5**
A4GALT	**2.72**	**2.61**	1.7	34.6	**0.4**
MGAT3	**1.84**	**5.95**	1.7	**2.4**	**0.1**
CSGALNACT1	**1.73**	**4.11**	1.5	21.0	**2.3**
CHST3	0.05	**3.69**	1.5	**9.2**	**0.1**
ST3GAL3	**0.93**	0.28	1.5	**7.1**	**0.6**
B3GNT6	**3.87**	**0.94**	1.3	**0.1**	**0.0**
B3GNT4	-0.89	**0.44**	1.2	**0.3**	**0.1**
ST3GAL5	0.15	**1.23**	1.2	**6.0**	**5.9**
FUT7	-1.60	**0.61**	1.2	**0.3**	**1.0**
B3GALT1	-1.05	-3.15	1.1	**0.0**	**0.0**
CHST4	**2.65**	NA	1.0	**0.0**	**0.0**
TUSC3	-1.18	-4.40	1.0	38.5	**0.1**
CHST11	**1.33**	**3.77**	0.9	26.0	18.1
GALNT9	-3.85	NA	0.8	**0.9**	**0.0**
CHST6	**2.29**	-0.06	0.6	**2.7**	**0.1**
MAN1C1	**0.54**	**2.85**	0.6	**8.4**	**1.6**
HS3ST3B1	**1.07**	0.16	0.5	**9.2**	**6.2**
ST6GALNAC3	-0.93	-3.08	0.5	**4.3**	**0.3**
ST8SIA1	**2.75**	NA	0.4	**2.7**	**1.1**
GALNT14	-3.23	NA	0.4	**1.5**	**0.0**
CHST2	**1.13**	0.13	0.3	**10.9**	**7.4**
ST3GAL6	**0.65**	-1.26	0.3	**5.8**	**3.5**
GALNT4	-1.15	0.32	0.3	**0.0**	**0.0**
GALNT13	**2.81**	**1.43**	0.2	**0.6**	**0.0**
GALNT16	-2.31	-1.07	0.2	**1.1**	**0.0**
UST	**1.10**	-0.20	0.2	**2.7**	**0.5**
GCNT4	**0.44**	**1.95**	0.2	**1.8**	**0.8**
HS3ST5	-0.03	-0.90	0.2	**0.1**	**0.0**
B3GALT2	-0.07	NA	0.2	**0.1**	**0.7**
GCNT2	**1.64**	-3.09	0.2	**1.0**	**0.7**
B4GALNT1	**0.99**	NA	0.2	**4.4**	**0.0**
EXTL1	-1.17	NA	0.1	**0.2**	**0.0**
CHSY3	**1.72**	NA	0.1	**6.1**	**0.0**
GALNT15	**1.86**	0.37	0.1	**5.6**	**0.0**
HS3ST3A1	**4.71**	NA	0.1	**8.9**	**0.0**
ST8SIA5	0.10	NA	0.1	**0.0**	**0.0**
CHST1	**1.75**	NA	0.0	**9.0**	**0.1**
ST6GALNAC5	NA	**2.81**	0.0	**9.0**	**0.0**
NDST4	NA	NA	0.0	**0.0**	**0.0**
B3GAT1	NA	NA	0.0	**0.0**	**0.3**
MGAT5B	**3.20**	**0.91**	0.0	**0.2**	**0.0**
HS3ST2	NA	-0.80	0.0	**0.1**	**0.5**
SPAM1	NA	NA	0.0	**0.0**	**0.0**
NDST3	**2.95**	-1.27	0.0	**1.1**	**0.0**
HYAL4	NA	NA	0.0	**0.0**	**0.0**
NEU2	NA	NA	0.0	**0.0**	**0.0**
HS6ST3	NA	NA	0.0	**0.0**	**0.0**
HPSE2	NA	NA	0.0	**1.0**	**0.0**
FUT9	NA	NA	0.0	**0.0**	**0.0**
FUT5	NA	NA	0.0	**0.0**	**0.0**

log_2FC, log_2 fold change. Step-1 required % expressing cells >15% in tumor epithelium and log_2FC >0.4 for both epithelial comparisons. Step-2 retained genes with % expressing cells <15% in both stromal and immune compartments.
